# SREBP2-dependent lipid droplet formation enhances viral replication and deteriorates lung injury in mice following IAV infection

**DOI:** 10.1080/22221751.2025.2470371

**Published:** 2025-02-19

**Authors:** Xinsen Li, Lu Li, Jijing Tian, Ruijing Su, Jiali Sun, Yuli Li, Lige Wang, Hongye Zhou, Shuhan Sha, Jin Xiao, Hong Dong, Caiyun Huo, Yanxin Hu, Hanchun Yang

**Affiliations:** a National Key Laboratory of Veterinary Public Health and Safety, Key Laboratory of Animal Epidemiology of Ministry of Agriculture and Rural Affairs, College of Veterinary Medicine, China Agricultural University, Beijing, People’s Republic of China; b Infectious Disease Department, Peking University Third Hospital, Beijing, People’s Republic of China; c Key Laboratory of Veterinary Bioproduction and Chemical Medicine of the Ministry of Agriculture, Zhongmu Institutes of China Animal Husbandry Industry Co., Ltd, Beijing, People’s Republic of China; d Beijing Key Laboratory of Traditional Chinese Veterinary Medicine, Beijing University of Agriculture, Beijing, People’s Republic of China

**Keywords:** Influenza A virus, lipid droplets, SREBP2, viral replication, lung injury

## Abstract

Influenza A virus (IAV) is a significant zoonotic pathogen that poses a considerable challenge to public health due to its continuous mutations. Lipid droplets (LDs) have been shown to play an important role in the process of several viral infections. However, their role in IAV infection remains unclear. Here, we found that IAV infection altered the lipid metabolism and increased the content of LDs in the lungs of mice. In vitro, IAV infection also mediated the formation of LDs in A549 cells. Besides, inhibition of the formation of lipid droplets can significantly suppress IAV replication and the release of inflammatory factors, indicating that LDs could facilitate the virus replication and inflammatory response. Furthermore, we discovered that IAV infection could activate the SREBP2, a crucial lipid-regulating transcription factor that regulates the expressions of downstream proteins named HMGCR and HMGCS. HMGCR and HMGCS involved in the process of cholesterol synthesis, which further promoted the formation of LDs. Additionally, the use of fatostatin that specifically inhibits the maturation of SREBP2 was able to significantly suppress the viral replication of H5N1 in cells and effectively ameliorated IAV-induced lung injury in mice, which eventually promoted the survival rate of infected mice. Taken together, we demonstrate the essential roles of lipid metabolism and LD formation in IAV replication and pathogenesis, which may better facilitate the advancement of new strategies against IAV infection, especially the highly pathogenic H5N1 virus.

## Introduction

Influenza A virus (IAV) is a significant public health concern, causing severe respiratory diseases with a wide host range including humans, poultry and mammals [1]. According to World Health Organization (WHO) data, influenza is responsible for an estimated 290,000–650,000 deaths annually [2]. From 2009 onwards, the pandemic influenza virus (PDM/09 H1N1) has extended its reach from Mexico and North America to more than 215 countries, exhibiting an exceptionally effective capacity for human-to-human transmission [3]. Concurrently, H5N1, known for its high pathogenicity as an avian influenza (HPAI) virus, is capable of crossing the species barrier, posing a threat of triggering a pandemic [4]. On March 25, 2024, a fatal human infection with the HPAI A (H5N1) virus was reported in Vietnam [5]. This was followed by the identification of the HPAI A (H5N1) subtype 2.3.4.4b in dairy cattle and unpasteurized milk in several U.S. states in April 2024, with a confirmed case of a dairy worker in Texas contacting the virus [6,7]. Vaccination remains a primary strategy for preventing influenza virus infection; however, the effectiveness of vaccines is being challenged by the emergence of new variants resulting from antigenic drift and shift [8,9]. The clinical utility of certain antiviral drugs such as zanamivir (Relenza) and oseltamivir (Tamiflu), is also being compromised due to the increasing prevalence of resistant strains [10,11]. Given these challenges, there is an urgent need to develop the innovative and broad-spectrum antiviral drugs to combat IAV effectively.

Host lipids and cholesterol play crucial roles in the viral replication cycle, influencing the processes such as viral entry, uncoating, assembly and release [12–14]. As an enveloped virus, IAV's replication mechanism is intricately linked to the host's lipid metabolism, which in turn significantly modulates the host's immune response to the infection [15,16]. Lipid droplets (LDs) are organelles widely present in eukaryotic cells, which are primarily responsible for the storage of neutral lipids such as triglycerides and cholesterol. LDs are dynamic and can interact with other cellular components like the endoplasmic reticulum, Golgi apparatus, mitochondria and peroxisomes, playing crucial roles in lipid synthesis, storage, degradation and recycling [17]. Recent research has indicated that LDs can serve as platforms to facilitate viral replication, and IAV infection leads to the accumulation of LDs. Nevertheless, the underlying mechanisms have not been yet fully understood [18,19].

Sterol Regulatory Element-Binding Proteins (SREBPs) are transcription factors characterized by a basic helix–loop–helix-leucine zipper domain and are crucial for the regulation of lipid homeostasis [20]. Such transcription factors play important roles in the metabolism of fatty acids and cholesterol [21]. For example, SREBP1, encompassing its isoforms SREBP1-a and SREBP1-c, is mainly involved in the regulation of fatty acid and triglyceride synthesis [22]. SREBP2 maintains the cholesterol balance through a negative feedback loop, where the elevated cholesterol levels within the cell impede the SREBP2 maturation, thereby suppressing the gene expressions related to cholesterol synthesis and uptake [23]. Elevated SREBP2 levels have been observed during infections with various viruses, including Hepatitis C virus, Dengue virus, SARS-CoV-2 and Zika virus [24–27]. Moreover, SREBP2 activation can regulate the formation of inflammatory complexes and promote the expressions of inflammatory factors [28]. Given these insights, previous studies have suggested that targeting lipid metabolism regulated by SREBPs might serve as a comprehensive antiviral strategy to fight against virus infection [29]. However, it is still unclear whether SREBPs are involved in the pathogenesis of IAV infection and affect the lipid metabolism.

Hence, the correlation between IAV infection and lipid metabolism was deeply investigated in this study. We initially embarked on a statistical analysis of 163 blood test results from influenza virus-infected patients at Peking University Third Hospital between 2017 and 2024, discovering a significantly higher prevalence of dyslipidemia in influenza-infected patients compared to the general population. Then, we conducted a non-targeted metabolomics analysis of the lung tissues in mice infected with H1N1 or H5N1, revealing a profound disruption of the host's lipid metabolism due to IAV infection. Additionally, we elucidated the pivotal role of SREBP2 signalling pathway on the dysregulation of lipid metabolism induced by IAV infection, demonstrating that attenuating SREBP2 activation can impede viral replication in A549 cells in vitro. We furtherly leveraged a mouse infection model to assess the therapeutic potential of fatostatin (an inhibitor of SREBP2) in mice against IAV infection in vivo. Intraperitoneal administration of fatostatin within the mouse model led to a notable increase in survival rates, a reduction in viral load and mitigation of lung injury. The overarching aim of our research was to investigate an innovative antiviral approach by targeting the host's lipid metabolism, offering a novel perspective in the combat against IAV.

## Materials and methods

### Dyslipidemia analysis of blood in patients with influenza

The dataset of 163 blood test outcomes in patients with influenza from 2017 to 2024 was furnished by the Department of Internal Medicine at Peking University Third Hospital, ensuring the exclusion of any identifiable private patient information. Detailed data were presented in Supplementary Table 1 and Supplementary Table 2. The study was approved by institutional ethics committees of Peking University Third Hospital, and all recruits provided written informed consent.

### Viruses and cell lines

The H1N1 (A/WSN/33) virus was provided by Dr. George F. Gao of the Institute of Microbiology, CAS, China, and the working stocks were generated in Madin-Darby canine kidney cells (MDCK) [30]. The avian influenza virus H5N1 (A/Chicken/Henan/1/04) was isolated from infected chicken flocks. Virus titres were determined by standard plaque assay. The 50% lethal dose (LD50) in mice was determined as previously described [31]. All experiments with the H5N1 virus were conducted in a Biosafety Level 3 (BSL-3) containment laboratory approved by the Ministry of Agriculture of China.

The human lung adenocarcinoma cell line A549 was provided by the Cell Resource Center of Peking Union Medical College (Beijing, China), cultured in DMEM medium supplemented with fetal bovine serum (10%), penicillin (100 U / mL), and streptomycin (100 mg / mL), and incubated in a sterile incubator at 37 °C with 5% CO2.

### Mice and treatments

*Viral challenge and sample collection*: Forty BALB/c mice aged 6–7 weeks were purchased from Beijing Vital River Laboratory Animal Technology Co., Ltd (Beijing, China). Mice were raised in independently ventilated cages and received pathogen-free food and water. For the viral challenge, mice were anesthetized with Zoletil (1:7 in saline, Virbac, Carros, France) by intramuscular injection, and then were intranasally infected with a dose of single LD50 H5N1 or triple LD50 H1N1, respectively. Mice in the mock group received the same volume of sterile saline. At days 3 and 6 post-infection, eight mice in each group were sacrificed and lung tissue samples were collected, half of which were fixed in 4% paraformaldehyde solution and half frozen at – 80 °C.

*Treatments*: Forty-two SPF BALB/c mice were randomly divided into six groups (n = 7 for each group): the mock group, IAV group, IAV + Fatostatin 5, 10, 20, and 30 mg / kg, intraperitoneally (i.p.) groups. The mice from IAV + Fatostatin groups were given 100 μL fatostatin solution for three times in total, two days before IAV infection, on the day of infection and two days after infection. The mice in the mock and IAV groups alone were only received the same volume of sterile saline rather than fatostatin. Then, mice were anesthetized with Zoletil (1:7) and infected intranasally with H5N1 virus or PBS. The clinical manifestations, body weight and survival rate were monitored daily for at least 14 days after the viral challenge. Similarly, lung tissues were collected at day 3 and 6 post-infection, respectively.

All animal experiments were approved by the Animal Ethics Committee of China Agricultural University following the Regulations of Experimental Animals of Beijing Authority.

### Plasmid and small interfering RNA (siRNA) transfection

For the overexpression of SREBP2 protein, a plasmid encoding human SREBP2 (SREBP2-pcDNA3.1(+)-myc-His A) was purchased from GenScript Biotech **(**Nanjing, China**)**. The siRNAs used for knocking down SREBP2 in A549 cells in this study were synthesized by Gene Pharma Co., Ltd (Beijing, China). The siRNA-NC served as a negative control. The transfection processes were taken according to the manufacturer’s instructions. Three siRNAs targeting SREBP2 were applied in the experiment. The specific target sequence for siNC was 5’-UUCUCCGAACGUGUCACGUTT-3’, the siSREBP2-1 sequence was 5’-GGACCAUUCUGACCACAAUTT-3’, the siSREBP2-2 sequence was 5’-GCCAAGGAGAGUCUAUACUTT-3’, and the siSREBP2-3 sequence was 5’-CGAGGACUUUAAUCAGAAUTT-3’.

### Cell culture and treatments

A549 cells were seeded in 6-well plates at 1 × 10^6^ cells mL^−1^ and allowed to attach overnight. H1N1 or H5N1 were inoculated into cells at MOI = 1 (multiplicity of infection) for 1 h. After washing, DMEM supplemented with 1% bovine serum albumin was added and cultured for the indicated periods at 37 °C, 5% CO2 incubator. The infected cells were further cultured for 12 and 24 h, respectively.

To investigate the effects of atorvastatin (MCE, Shanghai, China), A922500 (Sigma-Aldrich, Shanghai, China), oleic acid (MCE, Shanghai, China) and fatostatin (MCE, Shanghai, China) on IAV-infected A549 cells, the appropriate concentration of reagents was preliminarily determined using the MTT (methyl thiazolyl tetrazolium) assay according to the manufacturer's protocol (Solarbio, Beijing, China). A549 cells were pre-treated with atorvastatin (5, 10 μM), A922500 (5 μM) and oleic acid (10 μM) for 2 h and then were infected with H5N1 at MOI = 1 for 1 h at 37 ℃. Subsequently, DMEM supplemented with 1% bovine serum albumin was added and cultured at 37 °C, 5% CO2 incubator for 12 and 24 h, respectively. For fatostatin, A549 cells were initially infected with H5N1 at MOI = 1 for 1 h and then were treated with fatostatin (2 μM), which were further cultured for 24 h.

### Immunofluorescence staining

The LDs and expression of IAV nucleoprotein (NP) were detected through immunofluorescence staining. Cells or tissues were fixed with 4% formaldehyde and permeabilized with 0.1% Triton X-100 for 15 min, and incubated with a blocking solution for 30 min, followed by the anti-influenza NP mAb (1:1000, Abcam, Cambridge, MA) in PBS at 4 °C for 12 h and a suitable secondary antibody at 1:1000 dilution for 1 h at 37 °C. LDs were stained with BODIPY 493/503 dye (2 μM) for 5 min, and cell nucleus was stained with DAPI (1 μg/mL) for 5 min (Sigma-Aldrich, Shanghai, China). Fluorescence images were captured using a Confocal Microscopy (FV10-ASM, Tokyo, Japan).

### Oil red O staining

A549 cells were seeded in coverslips. The cells infected or not were fixed using 4% formaldehyde and the LDs were stained with Oil Red O (Solarbio, Beijing, China) for 15 min at room temperature. The coverslips were mounted in slides using an antifade mounting medium (Solarbio, Beijing, China). Nuclear recognition was based on DAPI staining (1 μg/mL) for 5 min. Fluorescence was analysed by fluorescence microscopy with a 100 x objective lens (Olympus, Tokyo, Japan).

### Total cholesterol (TCHO) and triglyceride (TG) assay

A549 cells were sampled after infection with the influenza virus for 12 or 24 h as mentioned above. Then, the cells were centrifuged at 1000 g for 10 min and lysed by 2% TritonX-100. The TCHO and TG levels were measured by the TCHO assay kit and TG assay kit (Nanjing Jiancheng Bioengineering Institute, Jiangsu, China) according to the manufacturer's instructions, respectively.

### ELISA for IL-6 and TNF-α measurement

To measure the levels of interleukin-6 (IL-6) and tumor necrosis factor-alpha (TNF-α) in cell culture supernatants, commercially available ELISA kits for IL-6 (E-EL-H6156) and TNF-α (E-EL-H0109) from Elabscience Biotech (China) were used. The assays were performed according to the manufacturer's instructions.

### RNA extraction and qPCR

Total RNA from cells and lung tissues was extracted using Trizol reagent (Invitrogen, Carlsbad, CA) and cDNA was reverse transcribed using the EasyScript First-Strand cDNA Synthesis SuperMix (TransGen Biotech, Beijing, China) according to the manufacturer's instructions. The gene expression was quantified with SYBR Premix Ex Taq (Tiangen Biotech Co. LTD, Beijing, China) on a 7500 Real-Time PCR system (Thermo Fisher Scientific, Waltham, Massachusetts, USA). The primer sequences for all genes are listed in Supplementary Table 3.

### Histopathological changes

Tissues were removed from euthanized mice and fixed in 4% neutral formalin at room temperature for 7 days. Serial tissue sections at 4 μm were obtained after embedding in the paraffin. Each slide was stained with hematoxylin and eosin (H&E) and then examined under light microscopy (Olympus BX41, Tokyo, Japan). The histopathological scores of lungs were as follows: 0  =  no microscopic lesions; 1  =  extremely mild, characterized by mild desquamation of rare bronchial epithelial cells and slight hyperemia of alveolar walls; 2  =  mild, characterized by desquamation of rare bronchial epithelial cells, hyperemia and slight thickness of alveolar walls; 3  =  moderate, characterized by desquamation of bronchial epithelial cells, dilated alveoli, severe hyperemia, loosen and obvious edema of blood vessel walls and slight infiltration of inflammatory cells; 4  =  severe, characterized by hyperemia, hemorrhage, loosen and edema of blood vessel wall, greater infiltration of inflammatory cells and desquamation of many epithelial cells.

### Immunohistochemistry

Paraffin sections were dewaxed, dehydrated, and immersed in distilled water, and then were incubated in a 3% hydrogen peroxide solution at room temperature for 15 min, followed by incubation in 10% normal goat serum in PBS for 30 min to block non-specific binding sites before reacting with the anti-influenza nucleoprotein mAb (1:1000, Abcam, Shanghai, China) in PBS at 4 °C for 12 h. After rinsing with PBS, the slides were further incubated with goat anti-mouse immunoglobin G (IgG) conjugated with horseradish peroxidase (Zhongshan Golden Bridge Biotechnology, Beijing, China) at 37 °C for 1 h. Staining was visualized by adding chromogen diaminobenzidine (Zhongshan Golden Bridge Biotechnology, Beijing, China) at room temperature for 10 min in the dark, followed by counterstaining with hematoxylin and then examined by light microscopy (Olympus BX41, Tokyo, Japan). The Image-Pro Plus 6.0 software was used to measure the integrated optical density (IOD) of influenza virus NP in the lungs within 3 high-power fields for each sample, and the average values were calculated. The sampling of these sections was unbiased; all samples were coded, and the examinations were conducted by a single investigator.

### Western blot

A549 cells were sampled after infection with the influenza virus for 12 or 24 h, and mouse lung tissues were ground with magnetic beads at −20 °C. The total proteins were extracted from the samples using RIPA lysis buffer containing 1 mM PMSF (Beyotime, Beijing, China), and were centrifuged at 12000rpm for 10 min at 4 °C. The supernatants were collected, and the protein concentrations were measured using the BCA Protein Assay Kit (Beyotime, Beijing, China). Denatured protein samples were separated by 12% SDS-PAGE gel electrophoresis and transferred onto a polyvinylidene fluoride (PVDF) membrane (Millipore, Beijing, China). The membranes were then placed in 5% skim milk (BD Biosciences) at room temperature for 1.5 h to block the non-specific binding sites. After washing, the membranes were incubated with the specified antibodies overnight at 4 °C with gentle shaking and the related secondary antibodies for 1 h at room temperature. Protein bands were visualized using Western Lightning Plus-ECL (Perkin Elmer, MA, U.S.) and detected using the chemiluminescence gel imaging system (Tanon-5200Multi). The gray values of the protein bands were analysed using ImageJ software. GAPDH and β-actin were used as an internal reference protein to compare the expression levels of the target proteins. The rabbit anti-SREBP1 antibody and mouse anti-SREBP2 antibody were obtained from Abcam Company and Santa Cruz Biotechnology, respectively. The anti-GAPDH and β-actin monoclonal antibody were obtained from Solarbio, and the corresponding horseradish-peroxide-conjugated secondary antibodies were obtained from the Beyotime Institute of Biotechnology.

### TCID50 assay

To determine the 50% tissue culture infectious dose (TCID50), the cell supernatants were collected and sequentially diluted 10-fold in DMEM from 10^−1^–10^−6^, and the dilutions were inoculated into MDCK cells at 37 °C. At 1 h post-inoculation, the monolayers were rinsed with PBS and incubated with medium (1% FBS in DMEM) for 72 h at 37 °C. The TCID50 was calculated by using the Reed-Muench method.

### Untargeted metabolomics analysis by LC−MS

The metabolomics of lungs in mice were analysed by OE Biotech (Shanghai, China). Briefly, approximately 15 mg of each lung sample was weighed and transferred to a new 1.5 mL tube. Next, 20 μL methanol containing the L-2-chlorophenyl alanine and 400 μL mixture of methanol and water (4:1) were added to each sample. The samples were then precooled, grinded and extracted by ultrasonic for 10 min in the ice-water bath. After centrifugation at 13,000 rpm for 10 min at 4 ℃, the supernatants were filtered through 0.22 μm microfilters and stored in LC glass vials at – 80 ℃ until the LC-MS analysis. QC samples were prepared by mixing aliquot of the all samples to be a pooled sample to evaluate the stability of the system’s mass spectrum platform throughout the experiment. A Dionex Ultimate 3000 RS UHPLC fitted with Q-Exactive plus quadrupole-Orbitrap mass spectrometer equipped with heated electrospray ionization (ESI) source (Thermo Fisher Scientific, Waltham, MA, USA) was used to analyse the metabolic profiling in both ESI positive and ESI negative ion modes.

For data preprocessing and identification of differential metabolites, Original LC-MS data were processed by the software Progenesis QI V2.3 (Nonlinear, Dynamics, Newcastle, UK) for baseline filtering, peak identification, integral, retention time correction, peak alignment, and normalization. Compound identification was based on the precise mass-to-charge ratio (M/z), secondary fragments, and isotopic distribution using the Human Metabolome Database (HMDB), Lipidmaps (V2.3), Metlin, EMDB, PMDB, and self-built databases to do qualitative analysis. The extracted data were then further processed by removing any peaks with a missing value (ion intensity = 0) in more than 50% of groups, replacing the zero value with half of the minimum value, and screening according to the qualitative results compound. Compounds with scores below 36 (out of 60) points were also deemed inaccurate and removed. A data matrix was combined from the positive and negative ion data. The matrix was imported in R to carry out Principle Component Analysis (PCA) to observe the overall distribution among the samples and the stability of the whole analysis process. Orthogonal Partial Least-Squares-Discriminant Analysis (OPLS-DA) and Partial Least-Squares-Discriminant Analysis (PLS-DA) were utilized to distinguish the metabolites that differ between groups. To prevent overfitting, 7-fold cross-validation and 200 Response Permutation Testing (RPT) were used to evaluate the quality of the model. Variable Importance of Projection (VIP) values obtained from the OPLS-DA model were used to rank the overall contribution of each variable to group discrimination.

### Statistical analysis

Data were presented as mean ± SEM, and differences were determined to be statistically significant by performing unpaired two-tailed t-tests or one-way/two-way analysis of variance (ANOVA) using GraphPad Prism 9.5.1. *P* < 0.05 was considered as statistical significance. In the analysis of metabolomic results, a two-tailed student’s t-test was used to verify whether the metabolites of difference between groups were significant. Differential metabolites were selected with VIP values greater than 1.0 and *P* < 0.05.

## Results

### Increased dyslipidemia prevalence in patients with influenza

**Figure 1.**
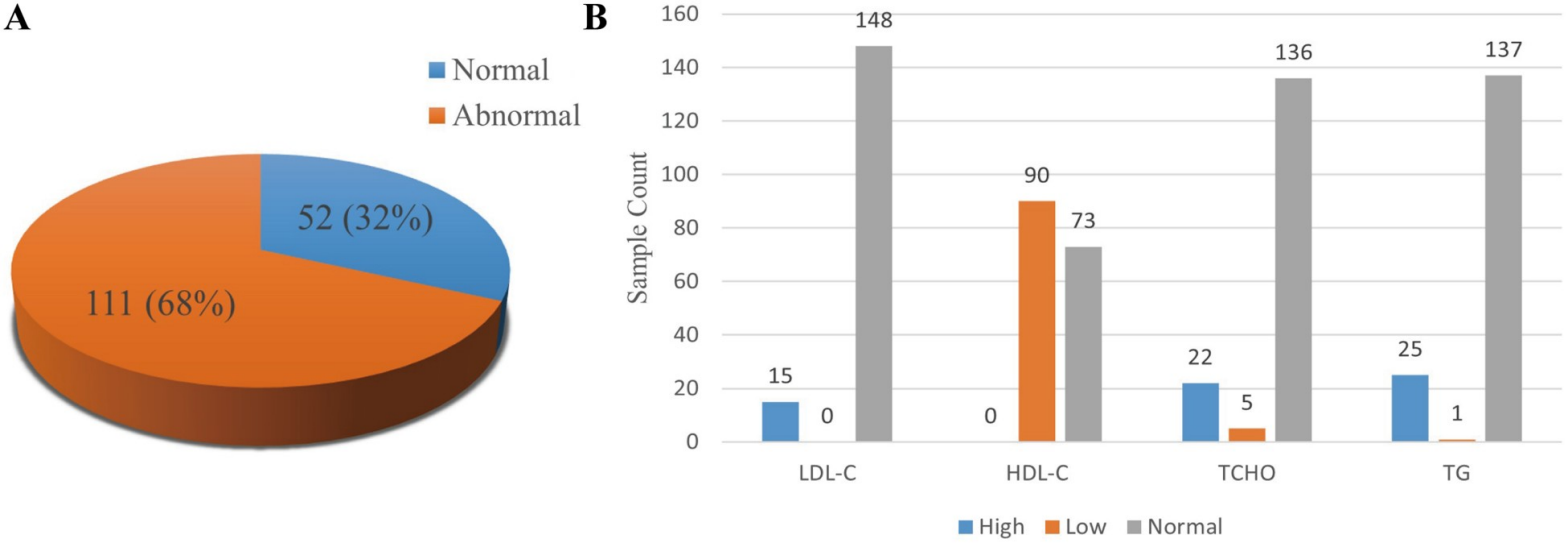
Analysis of dyslipidemia in blood test results of patients with influenza. The 163 blood test results of patients with influenza from the Department of Internal Medicine at Peking University Third Hospital from 2017 to 2024. (A) The proportion of samples with dyslipidemia. (B) Counts of samples with different types of dyslipidemia.

To investigate the correlation between IAV and lipid metabolism, we firstly analysed the 163blood test results of patients with influenza from the Department of Internal Medicine at Peking University Third Hospital from 2017 to 2024. In the blood of influenza patients, the data analysis revealed that 68% of patients exhibited the dyslipidemia, which was markedly higher than the national rate of 35.6% among the adult population in 2018 [32] (Figure 1A). Specifically, 90 cases (55.21%) had the decreased high-density lipoprotein cholesterol (HDL-C) levels, which were significantly higher than the 24.9% observed in the normal cases [33–35] (Figure 1B). Besides, 13.50% (22 cases) presented the elevated levels of total cholesterol (TCHO) levels, which were also significantly higher than the 5.8% found in the normal cases. Additionally, we collected 50 cases, which included blood test results during influenza infection, as well as pre – and post-infection. Among these, 36 cases exhibited abnormal lipid levels at the time of influenza diagnosis. Of these, 75% (27 cases) showed lipid changes attributable to the influenza infection, and 14 cases showed a worsening of lipid abnormalities following the diagnosis, while 9 cases demonstrated an improvement in lipid profiles after recovering from the infection. Together, these data suggest that influenza virus infection may dysregulated the lipid metabolism.

### IAV infection alters lipid metabolism in the lungs of mice

To gain a deeper understanding of the alterations in host lipid metabolism following IAV infection, mice were infected with IAV at a dose of single LD50 H5N1 or triple LD50 H1N1, and the indicators of lipid metabolism in the lungs were evaluated. As shown in Figure. S1A, IAV infection induced the serious lung damage in mice, characterized by congestion, hemorrhage and lymphocyte infiltration. Notably, significant pathological changes were observed at day 3 and day 6 post-infection as revealed by the gross histopathological analysis. Hematoxylin and eosin (H&E) staining further exposed the severe tissue damages, including the excessive congestion and inflammatory infiltration (Figure S1B). The IHC staining with an IAV NP antibody showed more positive cells in lungs of IAV-infected mice than that in the non-infected mice, which were mainly distributed in the bronchiolar epithelial cells and alveolar epithelial cells (Figure S1C). Thus, the in vivo mice model infected with IAVs were successfully established.

**Figure 2.**
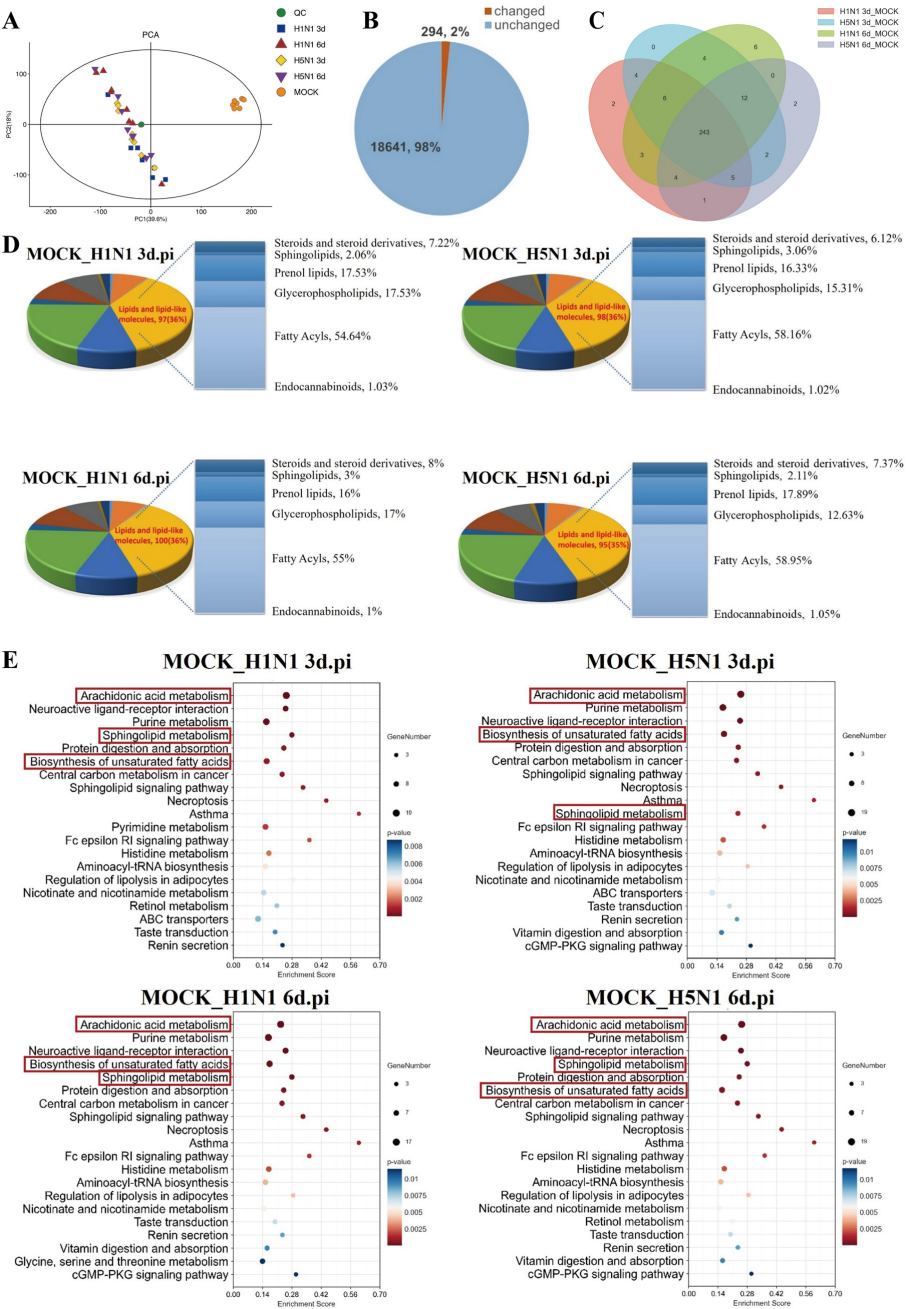
Multivariate data analysis of metabolic profiles induced by different IAV infections in mice. Mice were infected with a dose of single LD50 H5N1 or triple LD50 H1N1, and the lungs were collected at day 3 and 6 post-infection, respectively. Then, the lungs were subjected to non-targeted metabolomic analysis using the LC/MS. (A) PCA score chart for different IAV-infected groups. (B) Pie chart showing the numbers of altered metabolites in lungs of IAV-infected mice, which the significantly altered metabolites were in brown areas and non-significant metabolites in blue areas. (C) Venn diagram showing the overlaps among the altered metabolites in different IAV-infected groups. (D) Pie chart showing the numbers and proportions of different categories among all altered metabolites as well as the abundance of metabolites from different classes of altered lipids and lipid-like molecules. (E) The top 20 significant KEGG pathways associated with regulated metabolisms in IAV-infected groups when compared with the mock group.

Subsequently, the liquid chromatography-mass spectrometry (LC/MS)-based untargeted metabolomics was adapted for comprehensive detection and analysis of the metabolic alterations in the lungs of mice following IAV infection. Principal component analysis (PCA) score plots exposed a notable divergence in the metabolic profiles between the infected and control groups, underscoring the metabolic alterations in the lungs induced by IAV (Figure 2A). Rigorous data analysis, employing OPLS-DA with a variable influence on projection (VIP) threshold > 1 and student’s t-test at *P* < 0.05, identified 294 metabolites that were significantly diverged between infected and control groups (Figure 2B). Specifically, 268, 276, 278 and 269 distinct metabolites were identified in the H1N1 3 d.pi, H5N1 3 d.pi, H1N1 6 d.pi, and H5N1 6 d.pi groups, respectively (Figure 2C). Intriguingly, lipids and lipid-like molecules in infected groups were disproportionately affected, representing the 35-36% of the total altered metabolites, with their subtype distributions were statistically represented when compared with the non-infected mice (Figure 2D). The KEGG pathway enrichment analysis indicated the perturbation of pathways linked to the arachidonic acid metabolism, sphingolipid metabolism and the biosynthesis of unsaturated fatty acids occurred in both H1N1-infected and H5N1-infected mice at day 3 and 6 post-infection in comparison with the mock group (Figure 2E). Collectively, these findings demonstrate a significant alteration of pulmonary lipid metabolism in mice in response to IAV infection.

**Figure 3.**
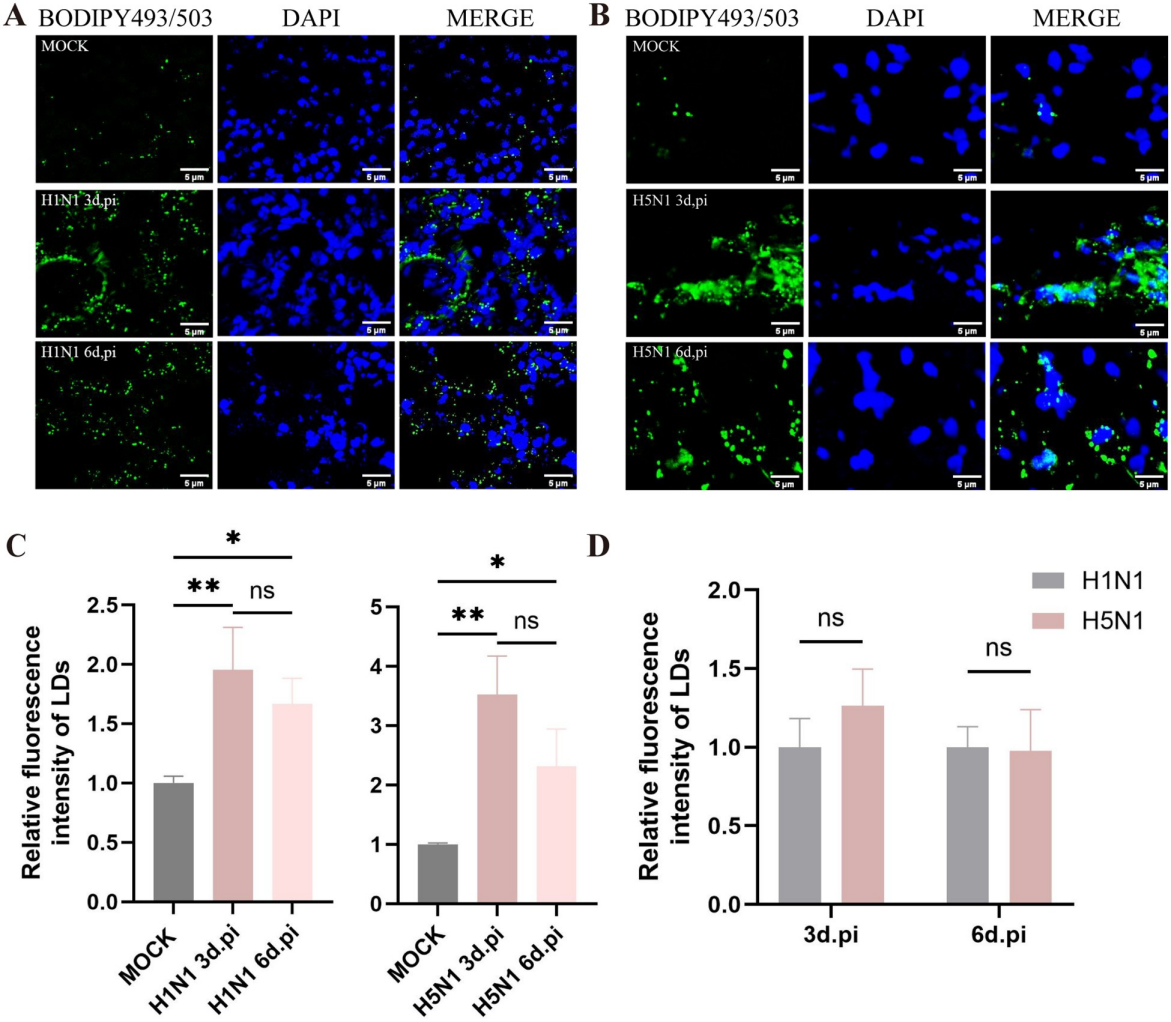
Fluorescent staining of LDs in lung tissues of mice infected with IAV. Mice were infected with a dose of single LD50 H5N1 or triple LD50 H1N1, and the lungs were collected at day 3 and 6 post-infection, respectively. Then, LDs in lung tissues were detected by the fluorescent staining. (A-B) LDs were stained with BODIPY 493/503 (green) and nucleus with DAPI (blue). (C-D) The quantification of relative fluorescence intensity of LDs was analysed using ImageJ software (n = 3). *P < 0.05, **P < 0.01. ns, no significance.

To further explore the lipid metabolic changes, the lung tissues were stained with the fluorescent dye BODIPY493/503, which had a high affinity for neutral lipids and LDs (ubiquitous organelles in most eukaryotic cells and serve as the storage sites for cholesterol and triglycerides). As displayed in Figure 3A–C, a significant increase in the levels of LDs was observed in lungs with H1N1 and H5N1 at both day 3 and day 6 post-infection. No significant difference of LDs levels could be seen between H1N1 and H5N1 groups (Figure 3D). Taken together, the results reveal that IAV infection can disrupt the lipid metabolism and increase the LDs formation in mice in vivo.

### IAV infection induces the LD formation in A549 cells to enhance viral replication

**Figure 4.**
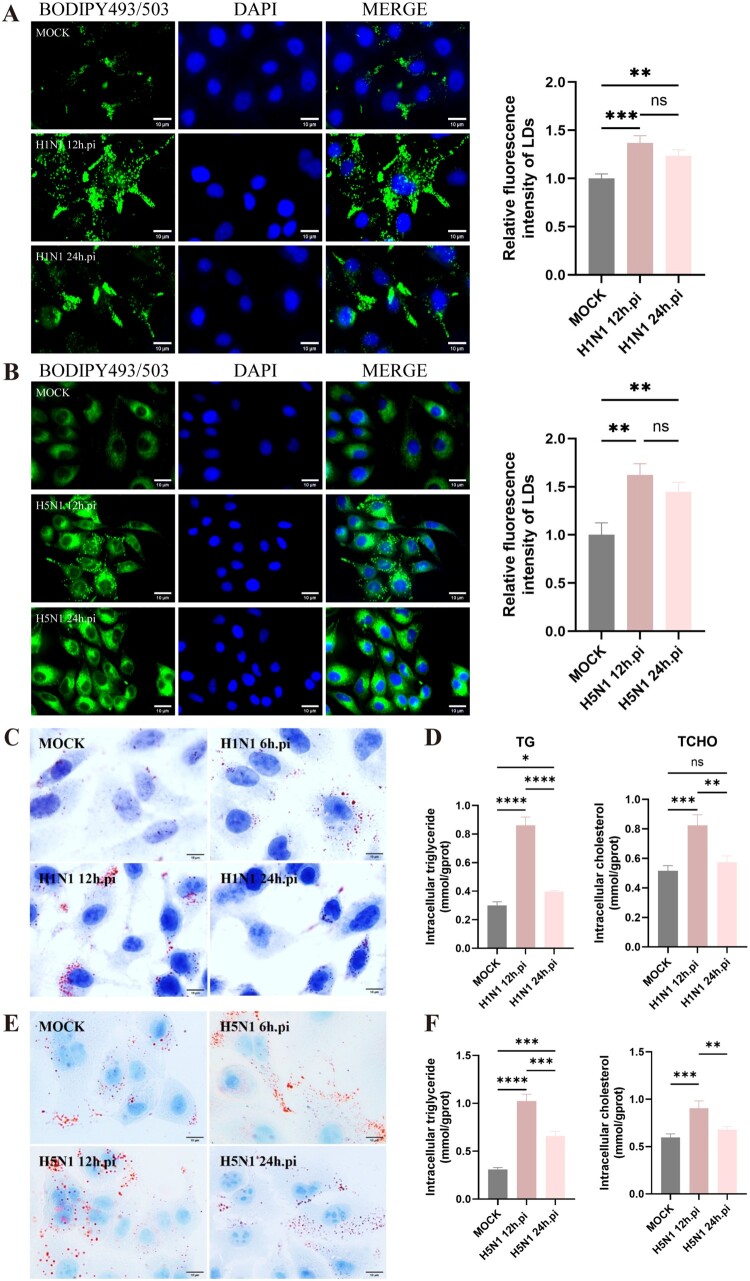
IAV infection induces LD formation in A549 cells. A549 cells were infected with H1N1 and H5N1 at MOI = 1, respectively. Then, the cells were collected at 12 and 24 h post-infection. (A-B) LDs were detected by the fluorescent staining. LDs were stained with BODIPY 493/503 (green) and nucleus with DAPI (blue). The quantification of relative fluorescence intensity of LDs was analysed using ImageJ software (n = 3). (C-D) LDs were detected by Oil Red O staining. (E-F) The amounts of TG and TCHO were quantified. *P < 0.05, **P < 0.01, ***P < 0.001, ****P < 0.0001. ns, no significance.

Previous experiments have demonstrated that IAV infection is able to disrupt the lipid metabolism in mouse lung tissue, leading to the accumulation of LDs. Consequently, we sought to explore whether this phenomenon would concurrently occur in cellular infections in vitro. As shown in Figure 4A and 4B, H1N1 and H5N1 infection at MOI = 1 could also result in the accumulation of LDs in A549 cells at 12 and 24 h post-infection, which the mean fluorescence intensity of LDs was significantly higher than that in the mock group. This phenomenon was also observed in A549 cells infected with H5N1 at a lower dose of MOI = 0.1 (Figure S2A). The Oil Red O staining further confirmed that both H1N1 and H5N1 infections upregulated the LD formation in A549 cells (Figure 4C–D). Moreover, biochemical assays revealed that IAV infections significantly elevated the TG and total TCHO levels in A549 cells when compared with non-infected cells, especially at 12 h post-infection (Figure 4E–F). Altogether, IAV infection can alter the cellular lipid metabolism to facilitate the formation of LDs.

**Figure 5.**
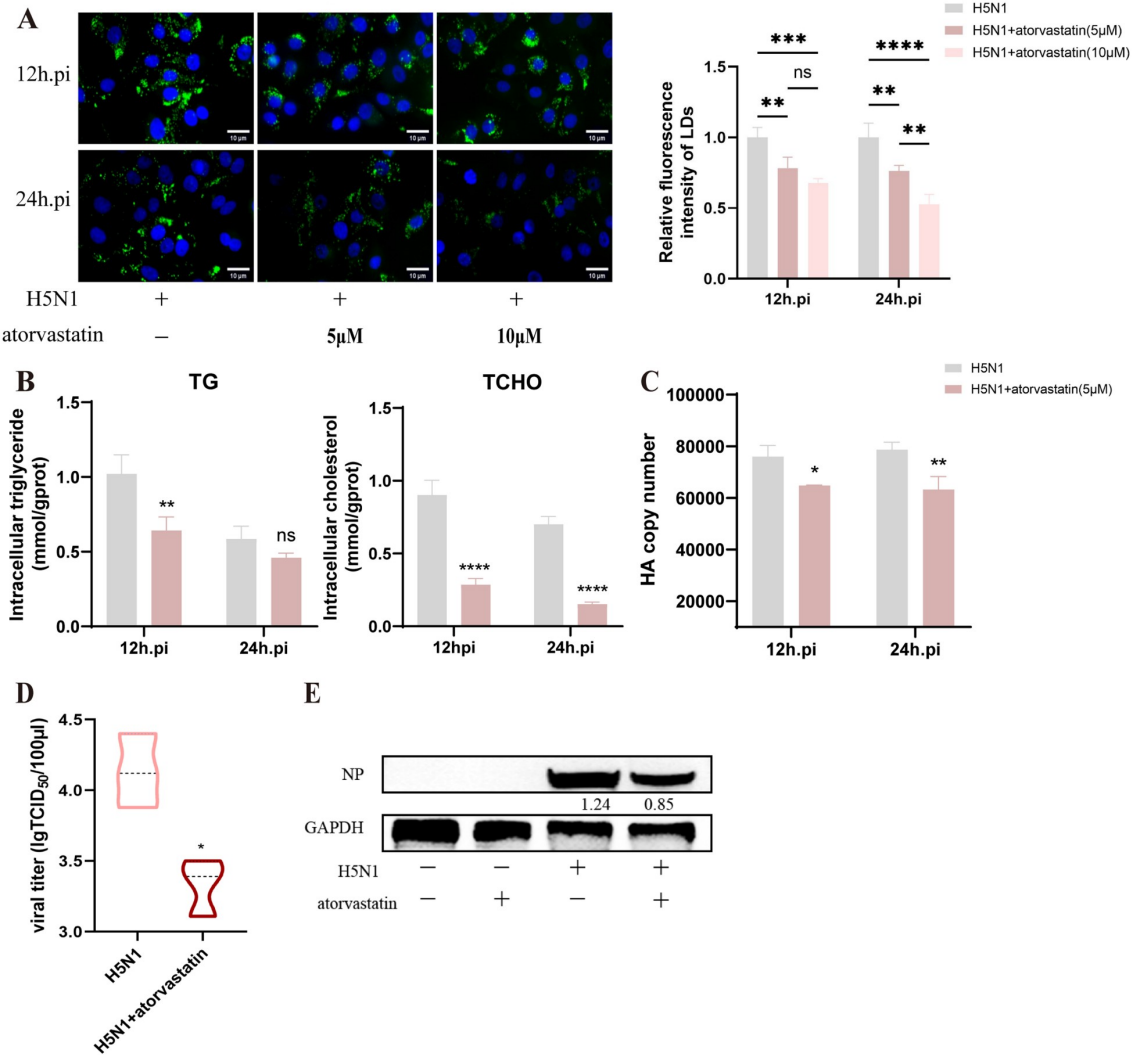
Inhibition of LDs suppresses IAV infection in A549 cells. (A) A549 cells were infected with H5N1 (MOI = 1) and then treated with atorvastatin (5,10 μM). At 12 and 24 h post-infection, LDs were detected by the fluorescent staining. LDs were stained with BODIPY 493/503 (green) and nucleus with DAPI (blue). The quantification of relative fluorescence intensity of LDs was analysed using ImageJ software (n = 3). A549 cells were infected with H5N1 (MOI = 1) and treated with atorvastatin (5 μM). (B-C) The amounts of TG, TCHO were quantified, and the HA copy number was detected by RT-qPCR (n = 3), respectively. (D) The viral titre was detected by TCID50 (n = 3). (E) The viral NP was detected by western blot. *P < 0.05, **P < 0.01, ***P < 0.001, ****P < 0.0001. ns, no significance.

Increasing evidence points out that LDs play an important role in the virus replication cycle, including as hubs for viral genome replication and viral particle assembling [36,37]. Since the above results that IAV infection could also induce LD formation in A549 cells, we further investigated the effect of LD formation on IAV replication. HMGCR is a key enzyme in the pathway of cholesterol biosynthesis, and atorvastatin, a specific inhibitor of HMGCR, can block LD synthesis [19,38]. Here, we pre-treated A549 cells with 5 μM or 10 μM atorvastatin for 2 h, followed by infection with H5N1 at MOI = 1, respectively. As seen in Figure 5A and 5B, atorvastatin significantly reduced the LD accumulation within the cells in a dose-dependent manner and also decreased the TCHO levels. Importantly, atorvastatin treatment significantly downregulated the mRNA levels of the viral HA gene, especially at 24 h post-infection (Figure 5C). Furthermore, the viral titre and viral NP protein also showed a significant decrease in H5N1 + atorvastatin group when compared with the H5N1 group (Figure 5D–E). In addition to this, we also utilized the A922500, an inhibitor of LD synthesis that targets the enzyme DGAT-1, a key enzyme involved in the final step of triacylglycerol synthesis and thus is central to remodel and finish the biogenesis of LDs. We found that treatment with A922500 sharply reduced both LD levels and the fluorescence of IAV-NP in H5N1-infected cells (Figure S3A). A922500 effectively inhibits the transcription of the IAV HA gene at the mRNA level, reduces the replication of the viral NP protein, and consequently lowers the viral titre (Figure S3B-D).On the contrary, supplementation with oleic acid, which enhances LD synthesis, was found to facilitate the viral replication. (Figure S3A-D). Collectively, these findings suggest that IAV infection can stimulate the LD biogenesis and upregulate the LD formation to promote the viral replication in A549 cells, and correspondingly, inhibition of the LD formation can suppress IAV infection.

### The SREBP2 signaling pathway mediates the LD formation in IAV-infected A549 cells

**Figure 6.**
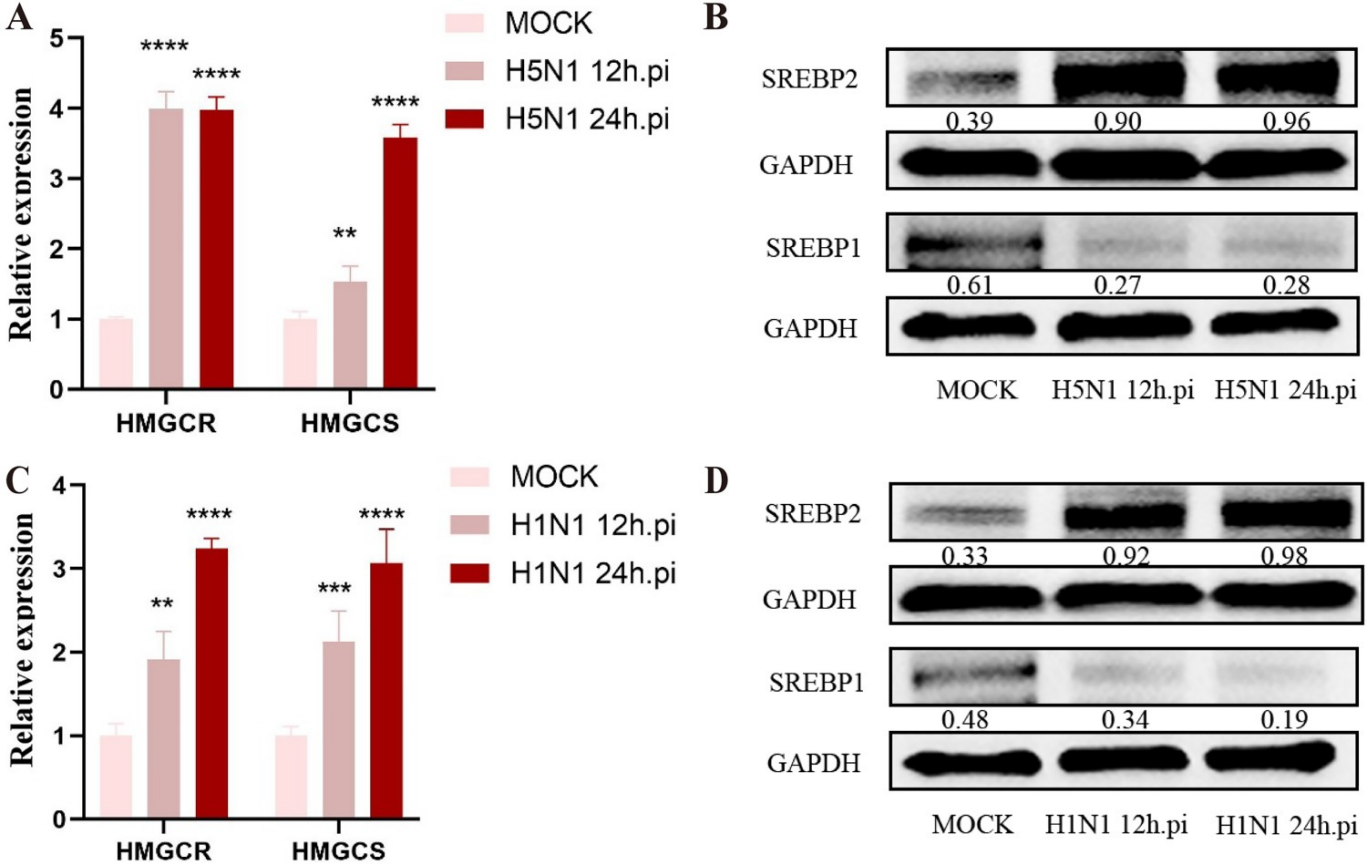
IAV infection modulates the lipid metabolism in A549 cells. (A-B) A549 cells were infected with H5N1 (MOI = 1), then the relative gene expressions of HMGCR and HMGCS were detected by RT-qPCR as well as the protein expressions of SREBP1 and SREBP2 were detected by western blot, respectively (n = 3). (C-D) A549 cells were infected with H1N1 (MOI = 1), then the relative gene expressions of HMGCR and HMGCS were detected by RT-qPCR as well as the protein expressions of SREBP1 and SREBP2 were detected by western blot, respectively (n = 3). **P < 0.01, ***P < 0.001, ****P < 0.0001.

LDs primarily store triglycerides and cholesterol. Since the aforementioned studies showed that IAV infection can reshape cellular lipid metabolism and increase the generation of LDs in cells, we further explored the related molecular mechanism behind this phenomenon. Initially, the expressions of HMGCR and HMGCS, two kinds of key enzymes in the cholesterol synthesis process, were assessed in IAV-infected A549 cells. As shown in Figure 6A, the mRNA levels of HMGCR and HMGCS were significantly increased in H5N1-infected cells at 12 and 24 h post-infection in comparison with those in non-infected cells, indicating that H5N1 could promote the cholesterol synthesis.

**Figure 7.**
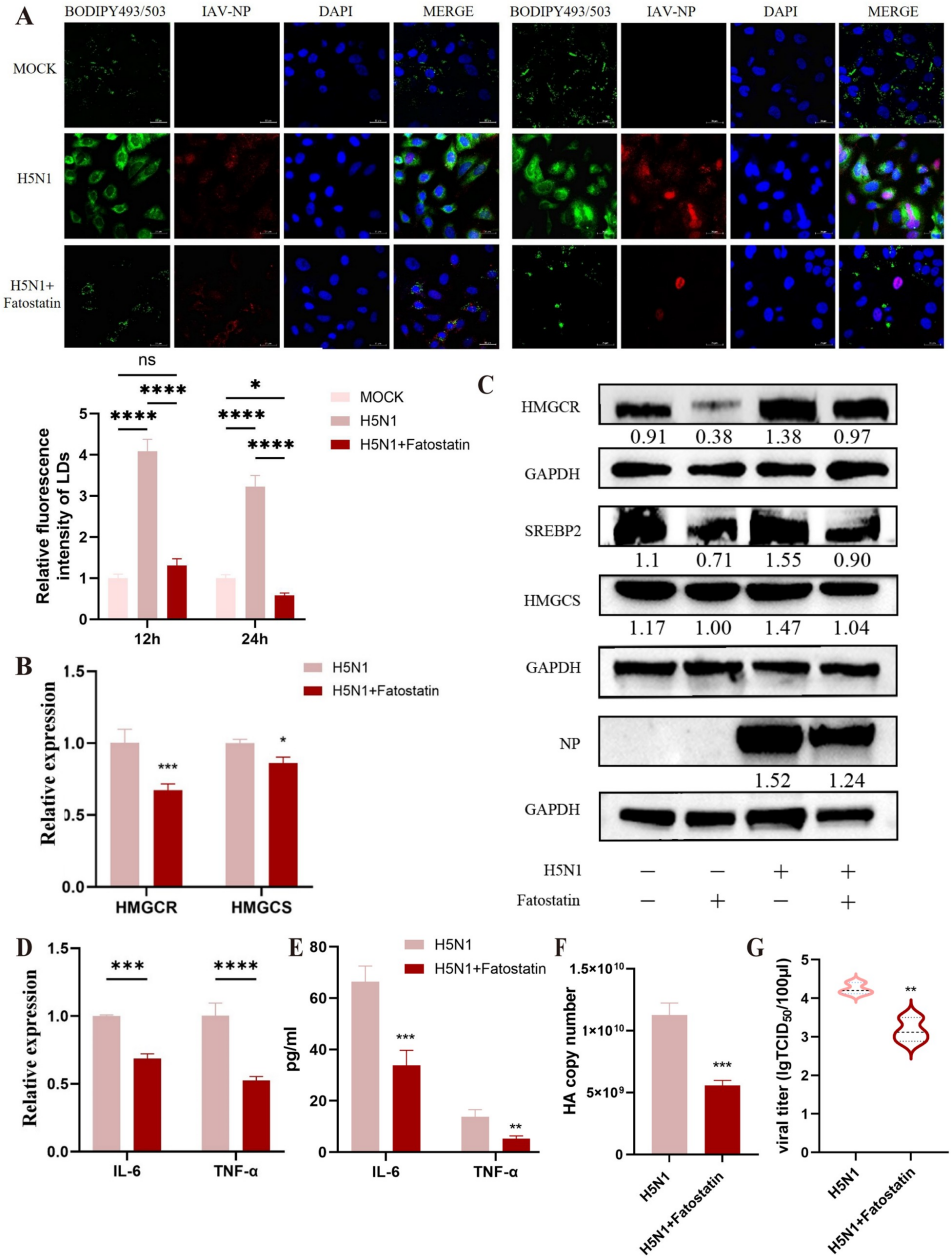
SREBP2 inhibitor fatostatin reduces virus-induced LDs formation and inhibits the H5N1 virus replication in A549 cells. A549 cells were infected with H5N1 (MOI = 1) and treated with fatostatin (2 μM). Then, the cells and supernatants were collected at 12 and 24 h post-infection. (A) LDs and NP expression were detected by the fluorescent staining. LDs were stained with BODIPY 493/503 (green), viral NP (red) and nucleus with DAPI (blue). The quantification of relative fluorescence intensity of LDs was analysed using ImageJ software (n = 3). (B) The relative gene expressions of HMGCR and HMGCS were detected by RT-qPCR (n = 3). (C) The protein expressions of SREBP2, HMGCR, HMGCS and viral NP in cells at 24 h post-infection were detected by western blot. (D-E) The expressions of IL-6 and TNF-α in cells at 24 h post-infection, respectively, determined using RT-qPCR and ELISA (n = 3). (F) The HA copy numbers were detected by RT-qPCR (n = 3). (G) The virus titres were assessed by TCID50 assay. *P < 0.05, **P < 0.01, ***P < 0.001, ****P < 0.0001.

**Figure 8.**
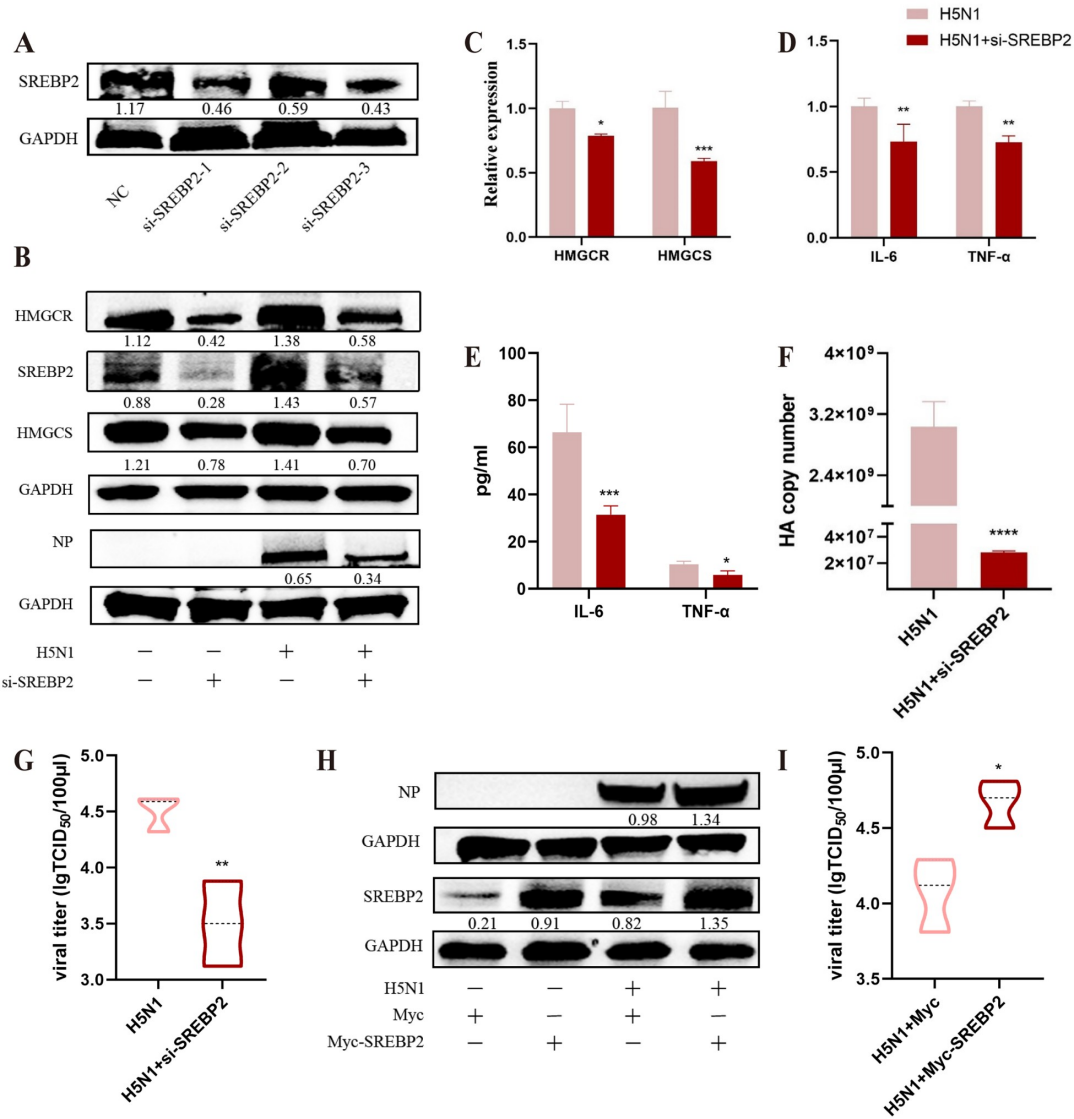
Knockdown or overexpression of SREBP2 affect the IAV-mediated lipid metabolism dysregulation and influenza H5N1 virus replication in A549 cells. A549 cells were transfected with siRNA targeting SREBP2 and infected with H5N1 (MOI = 1). (A) Western blot detected the protein expression of SREBP2 in A549 cells after siRNA transfection. (B) The protein expressions of SREBP2, HMGCR, HMGCS and viral NP in cells at 24 h post-infection were detected by western blot. (C) The relative gene expressions of HMGCR and HMGCS in cells at 24 h post-infection were detected by RT-qPCR (n = 3). (D-E) The expressions of IL-6 and TNF-α in cells at 24 h post-infection, respectively, determined using RT-qPCR and ELISA (n = 3). (F-G) The replication of the virus HA gene and the viral titre were detected by qPCR and TCID50, respectively (n = 3). In addition, A549 cells were transfected with plasmid to overexpress SREBP2 and infected with H5N1 (MOI = 1). (H) The viral NP were detected by western blot. (I) The viral titre were detected by TCID50 (n = 3). *P < 0.05, **P < 0.01, ***P < 0.001, ****P < 0.0001.

Furthermore, we also evaluated the cellular expressions of SREBPs that belong to the lipid synthesis regulatory factors. As seen in Figure 6B, H5N1 infection could upregulate the protein expression of SREBP2 rather than SREBP1 in A549 cells, indicating the transcription and activation of SREBP2 induced by H5N1 infection. Simultaneously, we also measured the impact of H1N1 on the lipid generation pathway, and the results were similar to those of H5N1, showing the increased levels of SREBP2, HMGCS and HMGCR (Figure 6C–D). Moreover, in order to deeply investigate the involvement of SREBP2 signalling pathway on IAV infection in cells, fatostatin, a pharmacological inhibitor known to impede the maturation of SREBP2 precursors, was used in this study. After treatment with fatostatin in A549 cells at 1 h post-IAV infection, the fluorescence staining revealed that fatostatin could significantly mitigated the IAV-induced LD accumulation and the fluorescence of IAV-NP in cells (Figure 7A). Additionally, the inhibitor also suppressed the IAV-mediated upregulation of SREBP2, leading to a significant reduction in the levels of both HMGCR and HMGCS in cells when compared to the H5N1 alone group (Figure 7B–C). In tandem, fatostatin treatment could also substantially decrease the expressions of pro-inflammatory cytokines IL-6 and TNF-α, IAV HA gene transcription and NP protein expression as well as viral titres (Figure 7C–F). At the same time, siRNAs for knocking down SREBP2 in A549 cells was used in this study to further substantiate our findings. As shown in Figure 8A, a significant downregulation of SREBP2 expression was shown in A549 cells following siRNA treatment (three siRNAs targeting SREBP2 were applied in the experiment; si-SREBP2-3 had the best interference effect and thus was used for interference tests in the later experiments). Likewise, knockdown of SREBP2 also led to a pronounced reduction in the expressions of HMGCR and HMGCS as well as the dramatical decrease in the expressions of IL-6 and TNF-α, IAV HA gene transcription and NP protein in IAV-infected A549 cells, and consequently lowers the viral titre (Figure 8B–G). Conversely, when we overexpressed SREBP2 into cells by transfection of the plasmid, we found that it increased the transcription of the HA gene and elevated the virus titres. Taken together, these results suggest that SREBP2 signalling pathway can be activated in A549 cells following IAV infection and plays important roles in the accumulation of LDs within cells, thereby aggravated the inflammatory responses and viral replication.

### Fatostatin improves the survival and ameliorates lung injury in mice following IAV infection

**Figure 9.**
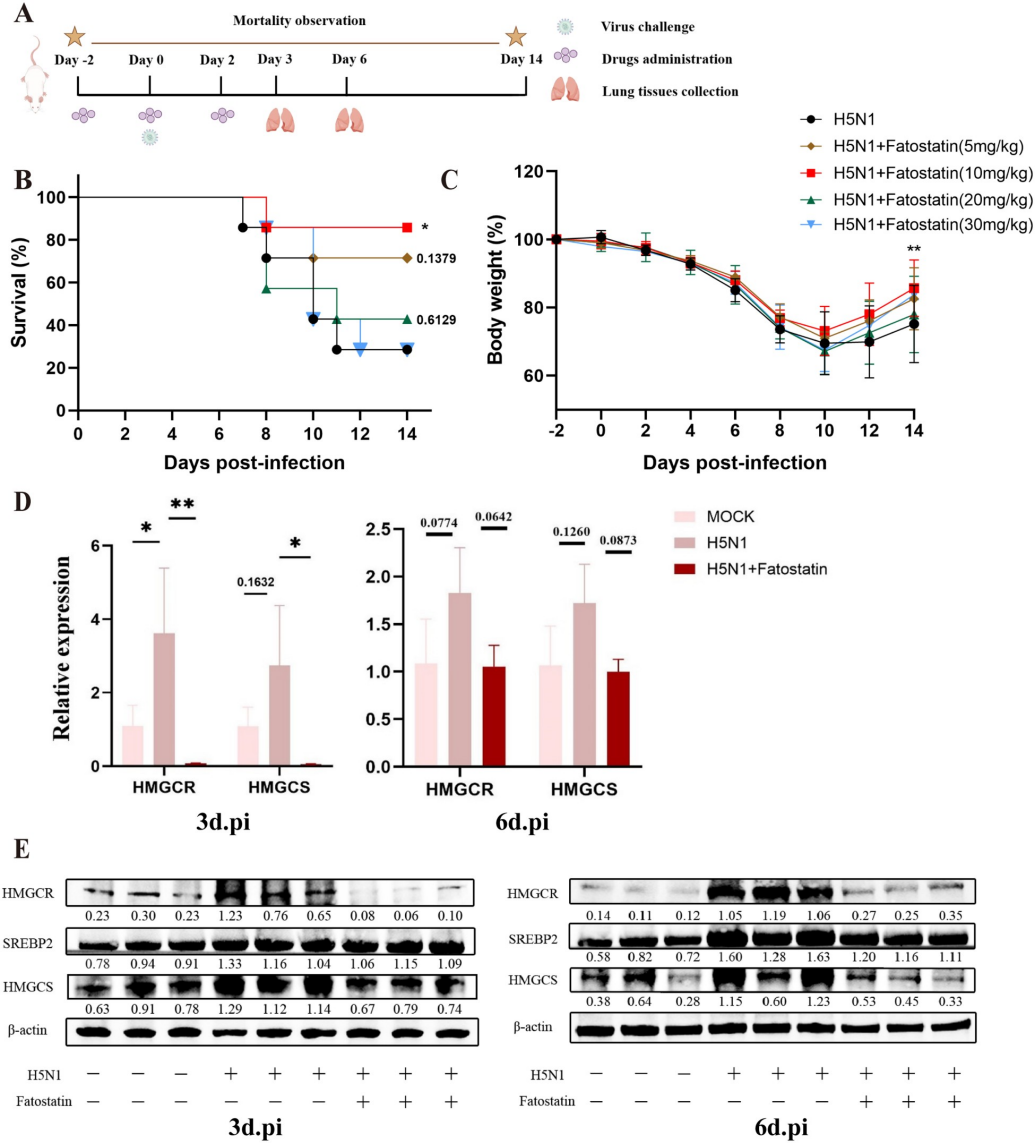
Fatostatin enhances the survival rate and alleviates the IAV-induced lipid metabolism dysregulation in mice. (A) The schematic of experimental design with the time points of fatostatin treatment (5, 10, 20 and 30 mg/kg, respectively), H5N1 virus challenge and lung tissue collection. Figures were created by Figdraw (www.figdraw.com). (B) Survival rates of H5N1-infected BALB/c mice treated with different doses of fatostatin (n = 7) and (C) percentage decrease relative to the initial body weight of mice. (D)The relative gene expressions of HMGCR and HMGCS in lung tissues of infected mice at day 3 (left) and 6 (right) post-infection were detected by RT-qPCR (n = 3). *P < 0.05, **P < 0.01. (E) The protein expressions of SREBP2, HMGCR and HMGCS in lung tissues of infected mice at day 3 (left) and 6 (right) post-infection were detected by western blot.

As mentioned above, inhibiting the activation of SREBP2 can reduce the influenza replication in A549 cells, we were then interested in determining whether administration of fatostatin could shield the mice from IAV infection. Mice received the intraperitoneal injections of fatostatin at varying doses (5, 10, 20, and 30 mg/kg) for three times in total, two days before IAV infection, on the day of infection, and two days after infection (Figure 9A). We found that mice received fatostatin treatment (5, 10 mg/kg) showed a significant increase in survival rates when compared to the infected control group (Figure 9B). Among the four different doses of fatostatin treatment, the mice receiving 10 mg/kg of fatostatin had the best survival rate and the lowest rate of weight loss (Figure 9C). Thus, the optimal protective dose of fatostatin (10 mg/kg) was used for the rest of the studies to further investigate its protective effects. Similar to cellular experiments in vitro, the treatment of mice with fatostatin also significantly inhibited the lipid metabolism in the lungs of mice infected with H5N1 at day 3 and 6 post-infection, which suppressed the gene and protein levels of HMGCR, HMGCS and SREBP2 (Figure 9D–E).

**Figure 10.**
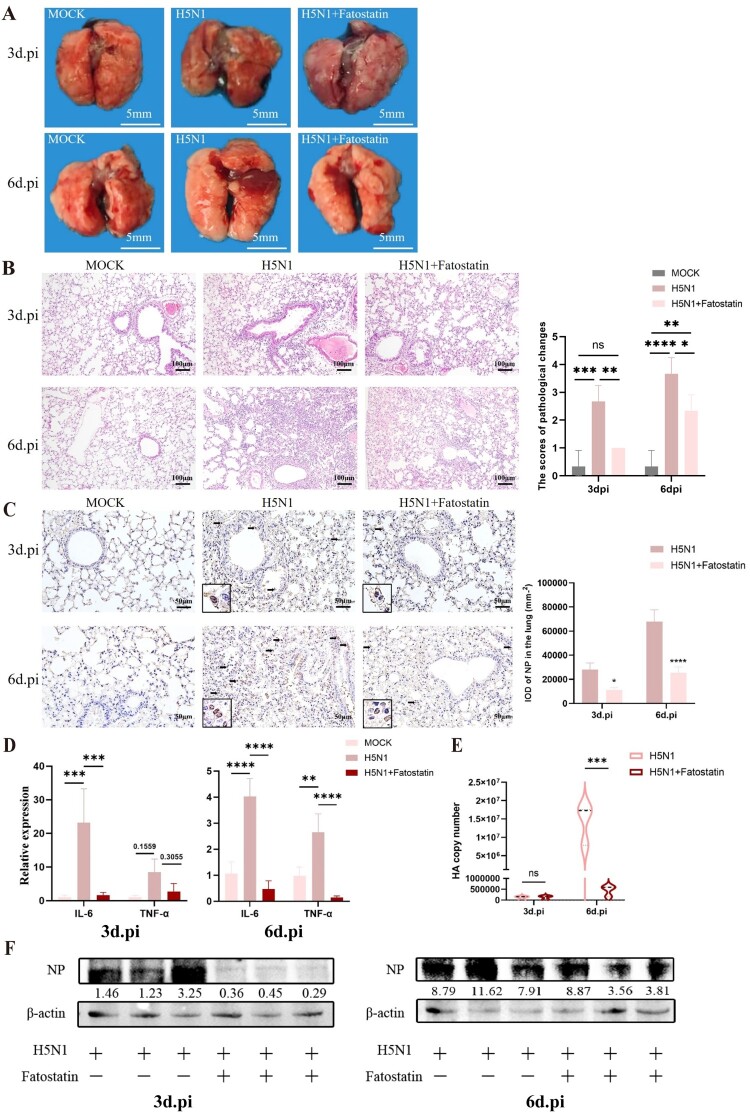
Fatostatin treatment relieves the acute lung damages caused by H5N1 infection. Mice were infected with a dose of single LD50 H5N1 and treated with fatostatin (10 mg/kg). (A) Clinical characteristics of lung tissue lesions at day 3 and 6 post-infection. (B) The histopathological changes of lung tissues at day 3 and 6 post-infection were assessed by H&E staining and scored by an examiner blinded to the study (n = 3). (C) The expression of viral NP in lung tissues at day 3 and 6 post-infection were assessed by IHC staining and scored by an examiner blinded to the study (n = 3). Black arrows indicate the positive signals. (D) The relative gene expressions of IL-6 and TNF-α in lung tissues at day 3 (left) and 6 (right) post-infection were detected RT-qPCR (n = 3). (E) The HA copy numbers were detected by RT-qPCR (n = 3). (F) The protein expressions of viral NP in lung tissues at day 3 (left) and 6 (right) post-infection were detected by western blot. *P < 0.05, **P < 0.01, ***P < 0.001, ****P < 0.0001. ns, no significance.

Furthermore, the effects of fatostatin treatment on the lung damages in infected mice were also assessed. As shown in Figure 10A, the gross lesions in the lungs of mice were found to be less severe in H5N1 + fatostatin group compared to the H5N1 group. Histopathological analysis revealed that H5N1 infection could cause the serious histopathological injuries, characterized by the detachment of bronchial epithelial cells, loose edema of perivascular connective tissue, congestion and hemorrhage in the alveolar walls, infiltration of large amounts of lymphocytes and inflammatory cells (Figure 10B). Immunohistochemical staining showed that the more positive signals were primarily present in macrophages, alveolar epithelial cells and bronchial epithelial cells in H5N1 group (Figure 10C). However, the less positive signals were found in the lungs in H5N1 + fatostatin group. Moreover, mRNA levels of TNF-α and IL-6 in the lungs were also lower in H5N1 + fatostatin group than those in H5N1 group (Figure 10D). Furthermore, the lungs of mice treated with fatostatin had the considerably decreased mRNA levels of viral HA gene at day 6 post-infection according to RT-qPCR results (Figure 10E). The western blot results also ensured that treatment with fatostatin could effectively inhibit the expression of the viral NP protein in the lungs of infected mice than that of untreated infected mice (Figure 10F). Collectively, fatostatin has a great protective effect on mice against IAV infection that can alleviate the lung injury and inhibition of viral replication as well as the inflammatory responses.

## Discussion

Influenza A virus (IAV) is a perennial public health challenge, with its seasonal epidemics causing significant disruptions to both public health systems and economic stability [39,40]. The detection of H5N1 in cattle herds and its transmission to farm workers, with fatal outcomes, further amplifies the threat to global health [5–7]. The continuous antigenic evolution of IAV through drift and shift results in new mutations that can undermine the efficacy of existing vaccines. Concurrently, the emergence of drug-resistant strains poses a substantial obstacle to the effectiveness of current antiviral therapies. These dynamics highlight an urgent demand for innovative pharmaceutical interventions to counter these evolving challenges. In this context, host-targeted antiviral strategies emerge as a promising avenue for exploration [41]. Lipid droplets, once considered mere storage organelles, are now recognized as multifunctional players in cellular physiology, and a growing body of evidence implicates their roles in viral replication processes. Previous research has demonstrated that infections caused by the Hepatitis C virus, Dengue virus, SARS-CoV-2, and Zika virus are associated with significant perturbations in the host lipid metabolism, culminating in the abnormal increase of LDs within the host cells [24–27]. Moreover, emerging evidences suggest that targeting the biogenesis of LDs presents a promising strategy to attenuate the replication across a range of viral pathogens, including Hepatitis C virus, Dengue virus, SARS-CoV-2 and Rabies virus [42–45]. Our study uncovers a robust correlation between LDs and IAV infection, adding a new dimension to our understanding of IAV pathogenesis and potential therapeutic targets.

As an enveloped virus, IAV relies on the host cell's lipid components for its membrane structure [46]. After infection, it has been shown to alter the host's lipid metabolism [47]. In our study, the analysis of blood test results from patients with influenza also revealed a certain correlation between influenza infection and dysregulation of lipid metabolism. The untargeted metabolomic profiling of lungs in mice following IAV infection validated the significant perturbations in pulmonary lipid metabolism, particularly in arachidonic acid metabolism, sphingolipid metabolism and the biosynthesis of unsaturated fatty acids. Concurrently, similar to the pathogens mentioned earlier, IAV infection also led to the accumulation of LDs in the lungs of mice. Additionally, cellular models of IAV infection corroborated these findings, showing a notable increase in intracellular LD content. This was in concordance with the observations made by Donna Episcopio et al., who reported a significant upregulation of LD levels in MDCK cells upon infection with H1N1 influenza virus at MOI = 5 [19]. Concurrently, we observed that during the replication of the influenza virus, lipid droplets initially accumulate but subsequently diminish. In the early stages of viral infection, the host cell's lipid metabolism is altered, which promotes the lipid production. As the virus replicates, it may utilize lipid droplet hydrolysis or lipid droplet autophagy to provide the energy or lipid materials for viral replication. This phenomenon has been confirmed during infections with viruses such as SARS-CoV-2, dengue virus, and hepatitis C virus [48–50].

To dissect the role of LDs in IAV infection, we employed the inhibitors to target LD biosynthesis, thereby reducing the intracellular LD levels. Atorvastatin, an inhibitor of HMGCR, the rate-limiting enzyme in cholesterol synthesis, effectively reversed the IAV-mediated accumulation of LDs and significantly curtailed viral replication. Similarly, an inhibitor of DGAT-1, the rate-limiting enzyme in LD synthesis, could suppress the viral replication and downregulated the expression of the viral NP. Thus, IAV-mediated LD accumulation has a promotingeffect on the viral replication. Interestingly, a previous study has demonstrated that the accumulation of LDs at the early stage of infection may actually be helpful for host to combat virus infection, potentially through their involvement in the early interferon response [51]. This observation presents a complex dichotomy in the role of LDs during viral infection, where their presence may be both beneficial and detrimental depending on the stage of infection and the perspective of the study.

Sterol Regulatory Element-Binding Proteins (SREBPs), pivotal transcriptional regulators of lipid metabolism, have been characterized for their involvement in the lipid metabolic dysregulation associated with a multitude of viral infections [26,27,52]. Our study explored the hypothesis that SREBPs may contribute to LD accumulation in IAV infection, a link that has yet to be firmly established in IAV pathophysiology. We identified a novel role of SREBP2 in IAV infection, indicating that IAV infection significantly upregulated the activation and expression of SREBP2. This upregulation modulated the downstream targets critical for cholesterol biosynthesis, including HMGCR and HMGCS [18], leading to the enhanced sterol production and intracellular accumulation of neutral lipids, and consequently, an increase in LD formation. These findings not only deepen our understanding of the metabolic reprogramming induced by IAV, but also highlight the potential therapeutic targets within the lipid metabolic pathways.

SREBPs could potentially act as a pan-antiviral target [53], which prompt our investigation into the possibility of leveraging SREBP2 as a host protein to combat IAV infection. By employing siRNA against SREBP2 and the SREBP2 inhibitor fatostatin, the IAV-mediated activation of SREBP2 can be effectively inhibited, and then reduced the expression of HMGCR and HMGCS. In line with our hypotheses, inhibition of SREBP2 activation mitigated the IAV-induced aggregation of LDs, significantly reducing the viral replication, NP expression and viral titre in cells. Additionally, the upregulation of pro-inflammatory cytokines IL-6 and TNF-α induced by IAV was also significantly attenuated, a result that may be associated with the ability of LDs to modulate immune and inflammatory responses [54]. In mouse experiments, fatostatin treatment at a dose of 10 mg/kg effectively mitigated the mortality and weight loss caused by IAV infection, inhibited the viral replication and alleviated the lung damages, thereby protecting mice from IAV infection. Here, we also found that fatostatin at high doses (> 10 mg/kg) has fewer effects on the survival rate of IAV-infected mice. Notably, previous researches have reported that fatostatin at high doses (eg: 30 mg/kg) can cause cell damages and even cell death such as inhibition of cell proliferation and ferroptosis in host cells [55–57]. Based on our results, we speculate that high doses of fatostatin may also result in the side effects in the viability of cells of IAV-infected mice, which then reduce the ability to protect mice against influenza virus infection.

**Figure 11.**
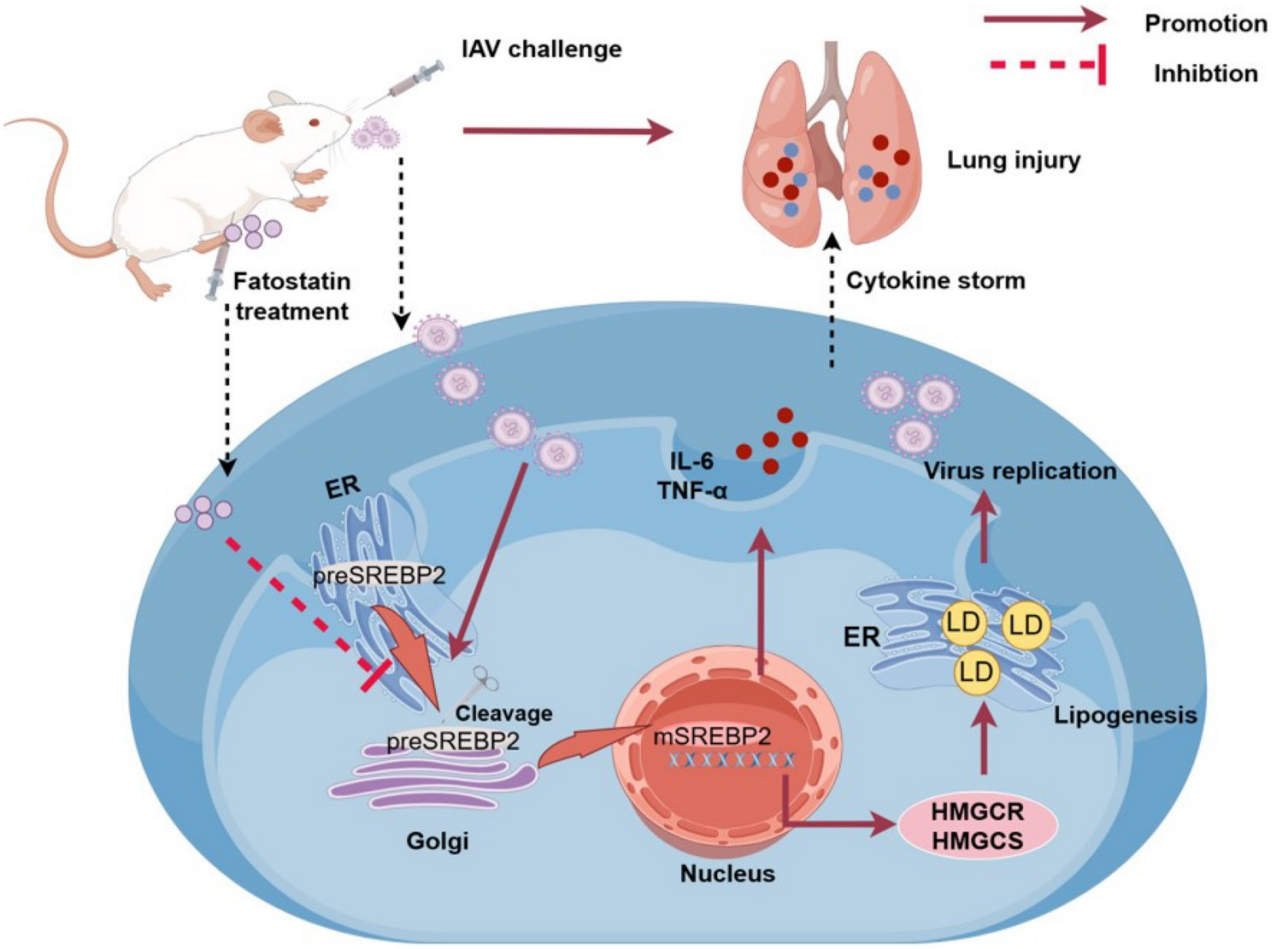
The summary illustration of potential mechanisms of the correlation between IAV and lipid metabolism. Figures were created by Figdraw (www.figdraw.com).

In conclusion, the findings underscore the pivotal role of LDs in IAV infection and, for the first time, demonstrate that targeted inhibition of SREBP2 can attenuate H5N1 replication and the inflammatory response in both cellular model and mice model. The targeted approach to inhibit the host protein SREBP2 can successfully reverse the activation induced by IAV infection and negate the overexpressions of its downstream targets, HMGCR and HMGCS. The inhibition of SREBP2 can also lead to a notable reduction in the lipid accumulation and a concomitant decrease in the levels of inflammatory cytokines. Moreover, fatostatin can effectively protect mice from H5N1 virus infection and alleviate the lung damages (Figure 11). The study will contribute to the development of novel strategies to combat H5N1 and other IAV infections.

## Author contributions

LL and YXH designed and conceived the study. XSL, RJS, JLS, YLL, SHS and LGW performed the in vivo experiment. XSL and HYZ performed the in vitro experiments. XSL, LL, CYH, YXH and HCY directed the data analysis. JJT, JX and HD helped the data analysis. XSL, CYH and YXH wrote the manuscript. All authors reviewed the manuscript and consented to the description of author contribution.

## Supplementary Material

Supplement Fig 2.tif

Supplement Fig 1.tif

Supplement Fig 4.tif

Supplementary Materials.pdf

Supplement Fig 3.tif

## Data Availability

All data generated or analysed during the study period are included in the paper. Additional data relevant to this study are available upon request.
