# Supplementary figures and images for: SREBP2-dependent lipid droplet formation enhances viral replication and deteriorates lung injury in mice following IAV infection
Source: Emerg Microbes Infect. 2025 Feb 19;14(1):2470371. doi: 10.1080/22221751.2025.2470371 (PMC11873989; doi:10.1080/22221751.2025.2470371)

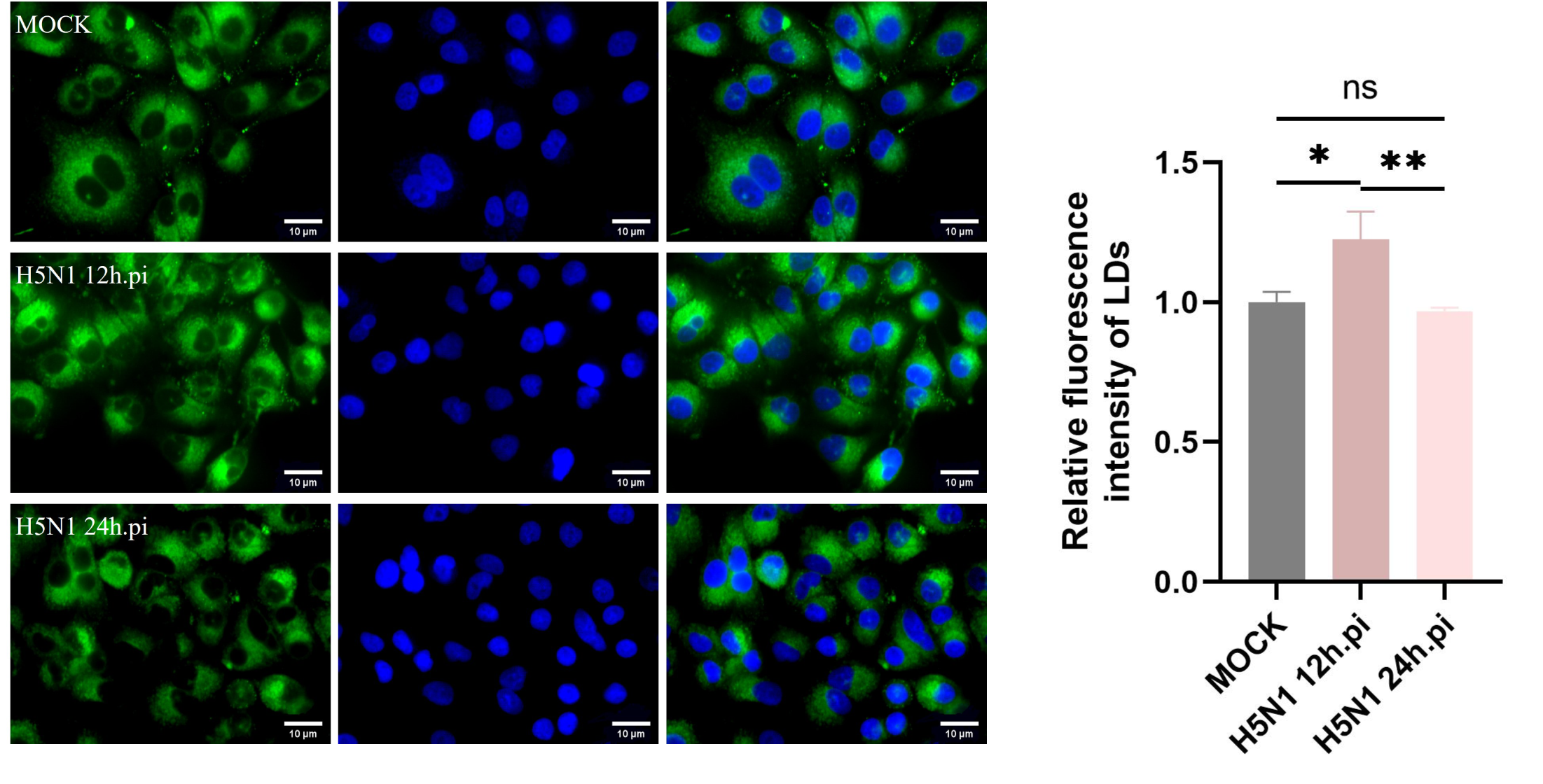

Supplement: Supplement Fig 2.tif [file TEMI_A_2470371_SM4238.tif]

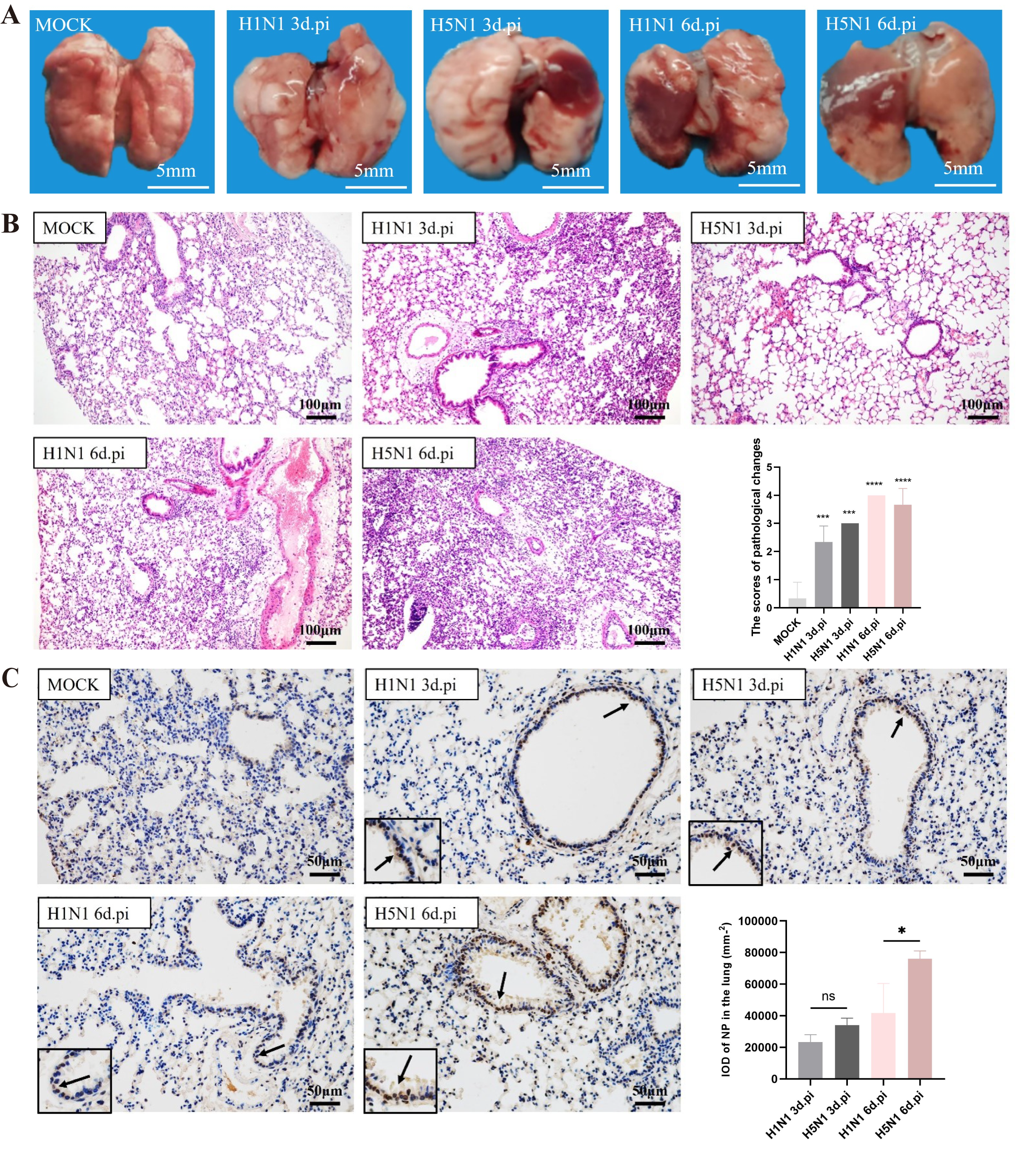

Supplement: Supplement Fig 1.tif [file TEMI_A_2470371_SM4237.tif]

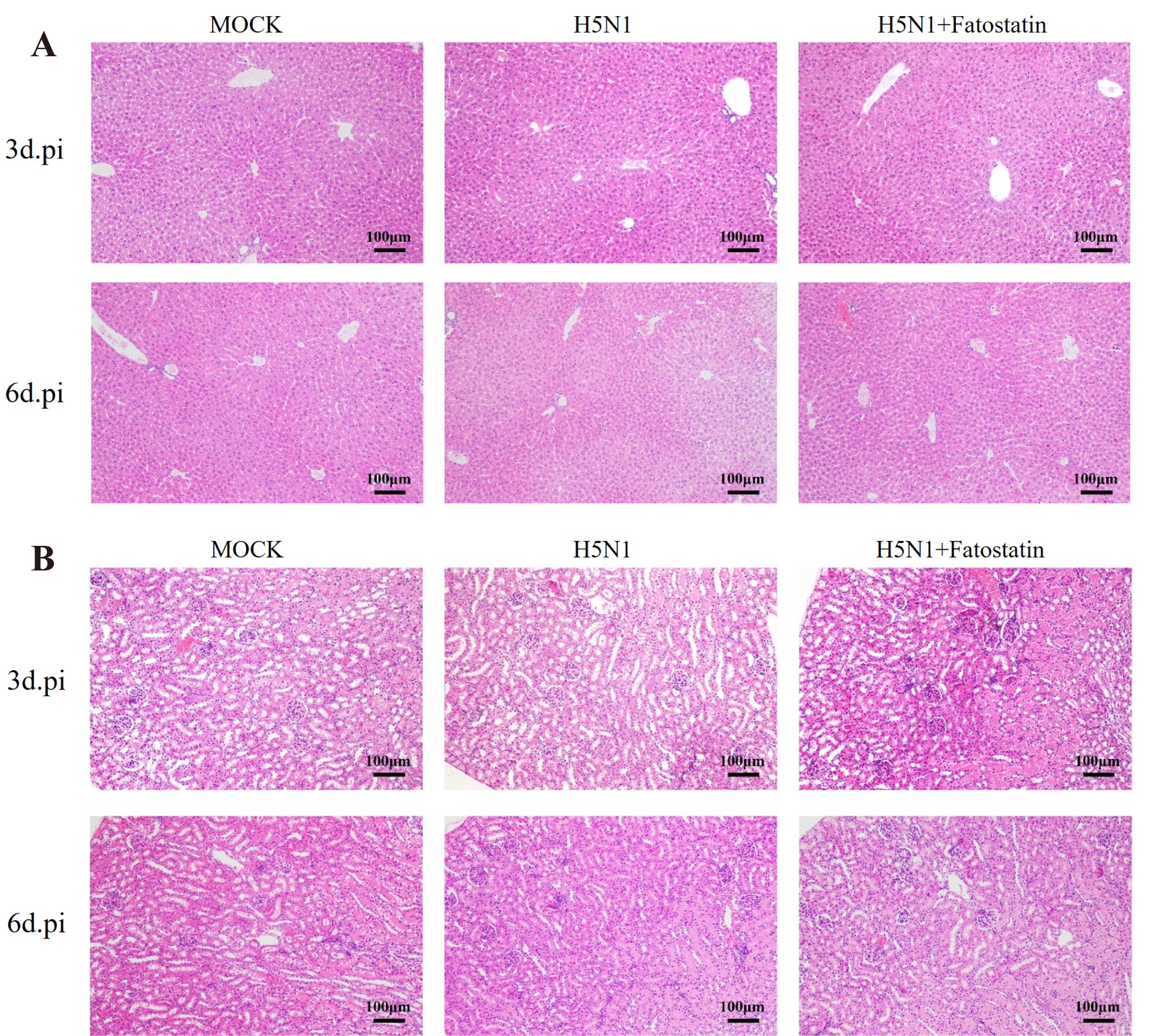

Supplement: Supplement Fig 4.tif [file TEMI_A_2470371_SM4236.tif]

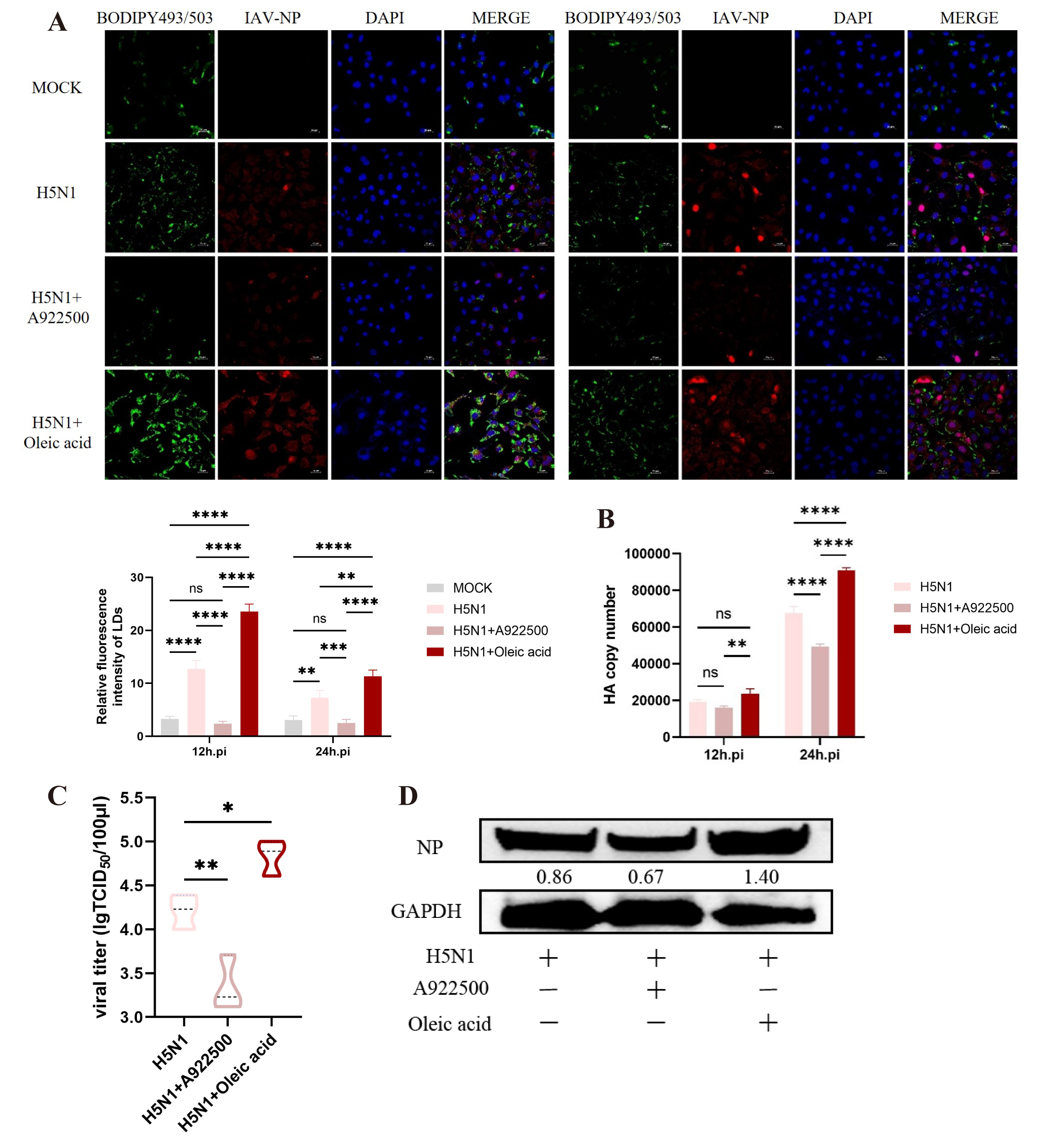

Supplement: Supplement Fig 3.tif [file TEMI_A_2470371_SM4234.tif]
