# Supplementary material for: SREBP2-dependent lipid droplet formation enhances viral replication and deteriorates lung injury in mice following IAV infection
Source: Emerg Microbes Infect. 2025 Feb 19;14(1):2470371. doi: 10.1080/22221751.2025.2470371 (PMC11873989; doi:10.1080/22221751.2025.2470371)
Supplement: Supplementary Materials.pdf [file TEMI_A_2470371_SM4235.pdf]

Supplementary Materials for

SREBP2-dependent lipid droplet formation enhances viral replication and deteriorates lung injury in mice following IAV infection

Xinsen Li[1#], Lu Li[2#], Jijing Tian[1], Ruijing Su[1], Jiali Sun[1], Yuli Li[1], Lige Wang[1], Hongye Zhou[1], Jin Xiao[3], Hong Dong[4], Caiyun Huo[1*], Yanxin Hu[1*], Hanchun Yang[1]

[1] National Key Laboratory of Veterinary Public Health and Safety, Key Laboratory of Animal Epidemiology of Ministry of Agriculture and Rural Affairs, College of Veterinary Medicine, China Agricultural University, Beijing, China.

[2] Infectious Disease Department, Peking University Third Hospital, Beijing, China.

[3] Key Laboratory of Veterinary Bioproduction and Chemical Medicine of the Ministry of Agriculture, Zhongmu Institutes of China Animal Husbandry Industry Co., Ltd, Beijing, China.

[4] Beijing Key Laboratory of Traditional Chinese Veterinary Medicine, Beijing University of Agriculture, Beijing, China.

# Contributed to the work equally.

*** Corresponding authors:**

Dr. Yanxin Hu, National Key Laboratory of Veterinary Public Health and Safety, Key Laboratory of Animal Epidemiology of Ministry of Agriculture and Rural Affairs, College of Veterinary Medicine, China Agricultural University, No.2 Yuanmingyuan West Road, Beijing, China. Email: huyx@cau.edu.cn

Dr. Caiyun Huo, National Key Laboratory of Veterinary Public Health and Safety, Key Laboratory of Animal Epidemiology of Ministry of Agriculture and Rural Affairs, College of Veterinary Medicine, China Agricultural University, No.2 Yuanmingyuan West Road, Beijing, China. Email: 1476893197@qq.com

This PDF file includes:

Figures. S1 to S4

Table S1 to S3

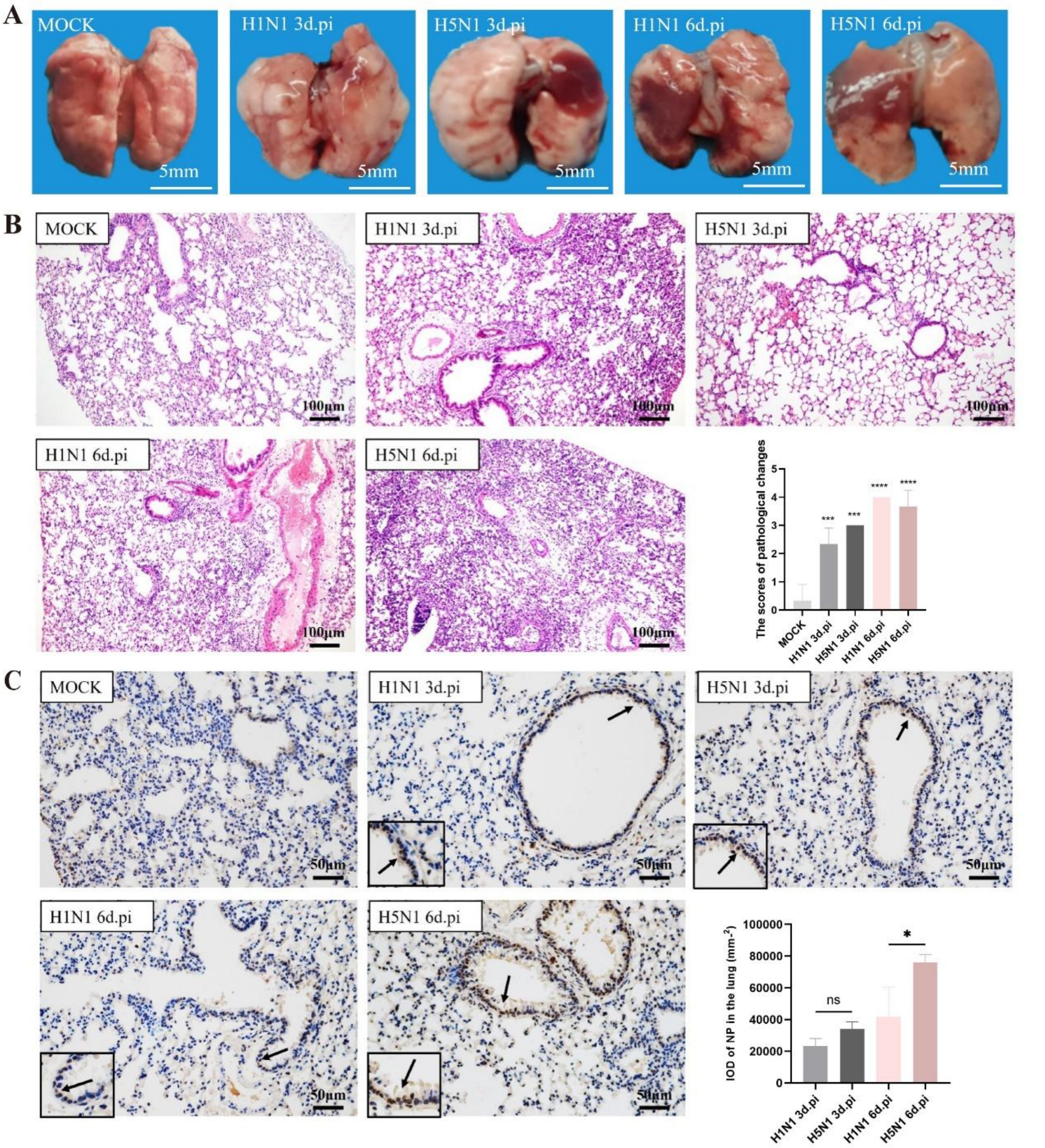

Fig. S1: IAV infection induces lung damage in mice. Mice were infected with H1N1 and H5N1, respectively. (A) Clinical characteristics of lung tissue lesions at day 3 and 6 post-infection. (B) The histopathological changes of lung tissues at day 3 and 6 post-infection were assessed by H&E staining and scored by an examiner blinded to the study (n=3). Black triangles indicate the lymphocyte infiltration. Black pentagons indicate the hemorrhage and hyperemia. Black arrows indicate the edema. (C) The expression of viral NP in lung tissues at day 3 and 6 post-infection were assessed by IHC staining and scored by an examiner blinded to the study (n=3). Black arrows indicate positive signals. *$P < 0.05$, ***$P < 0.001$, ****$P < 0.0001$. ns, no significance.

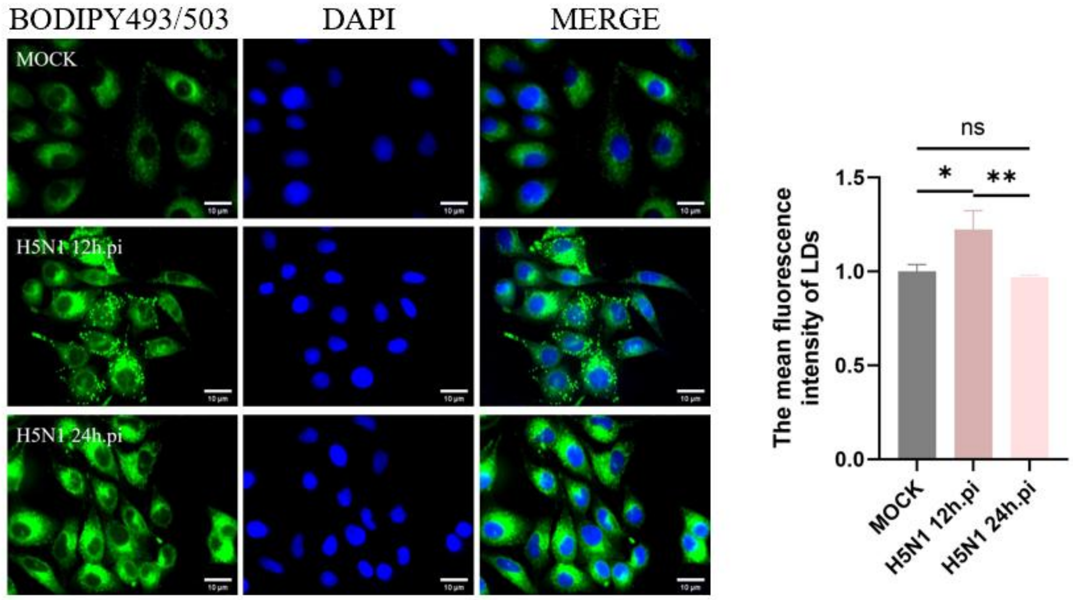

Fig. S2: IAV infection induces LD biogenesis in A549 cells. A549 cells were infected with H5N1 at MOI=0.1, then LDs in cells at 12 h and 24 h post-infection were detected by the fluorescent staining. LDs were stained with BODIPY 493/503 (green) and nucleus with DAPI (blue). The quantification of relative fluorescence intensity of LDs was analyzed using ImageJ software (n=3). *P < 0.05, **P < 0.01. ns, no significance.

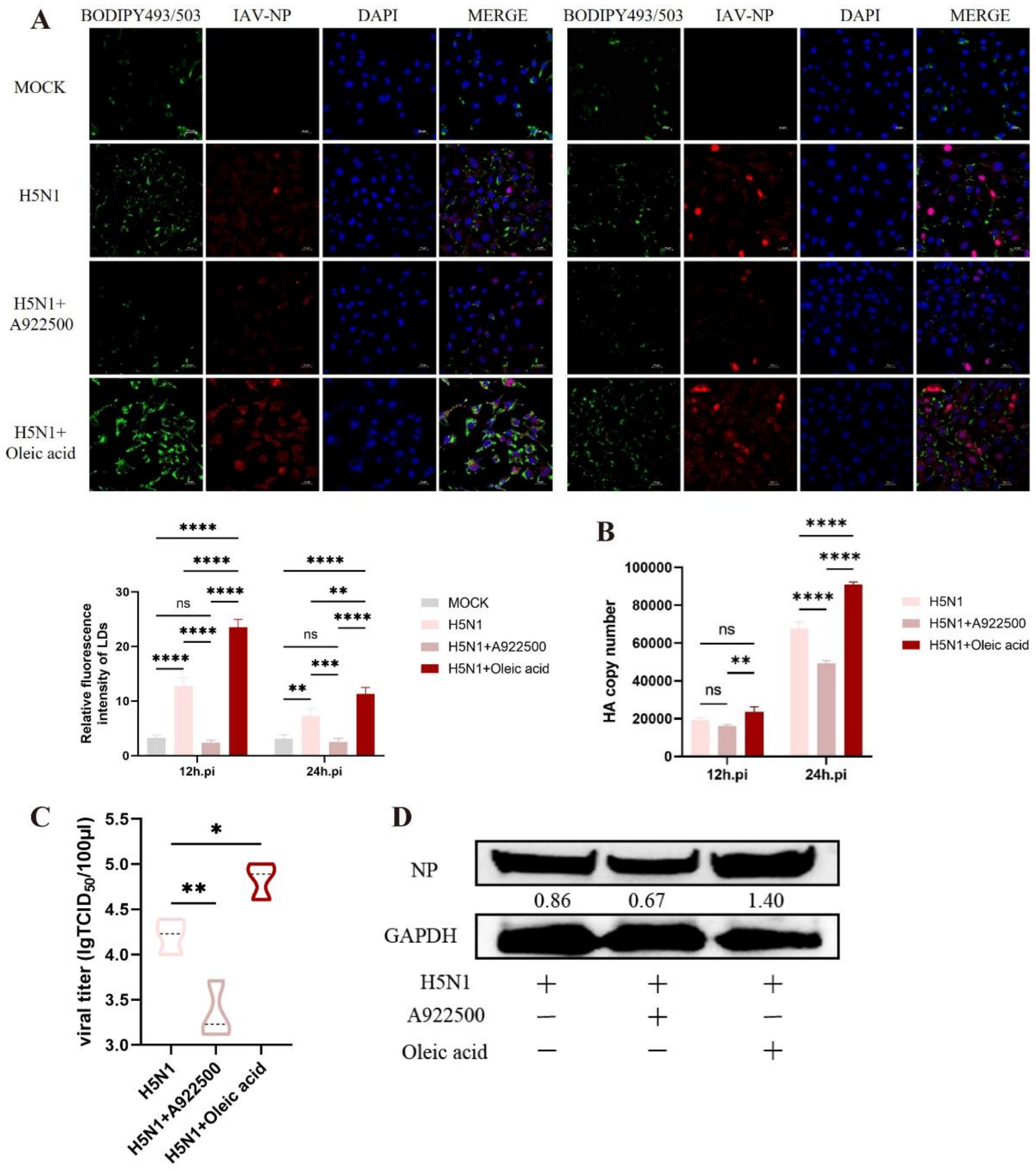

Fig. S3: The effects of A922500 and oleic acid on LDs in A549 cells. A549 cells were infected with H5N1 (MOI = 1) and treated with A922500 (2 µM) and oleic acid (10 µM), respectively. (A) LDs in cells at 12 h (left) and 24 h (right) post-infection were detected by the fluorescent staining. LDs were stained with BODIPY 493/503 (green), viral NP (red) and nucleus with DAPI (blue). The quantification of relative fluorescence intensity of LDs was analyzed using ImageJ software (n=3). (B) The HA copy numbers were detected by RT-qPCR (n=3). (C) The viral titer was detected by TCID50 (n = 3). (D) The viral NP was detected by western blot. **P < 0.01, ***P < 0.001, ****P < 0.0001. ns, no significance.

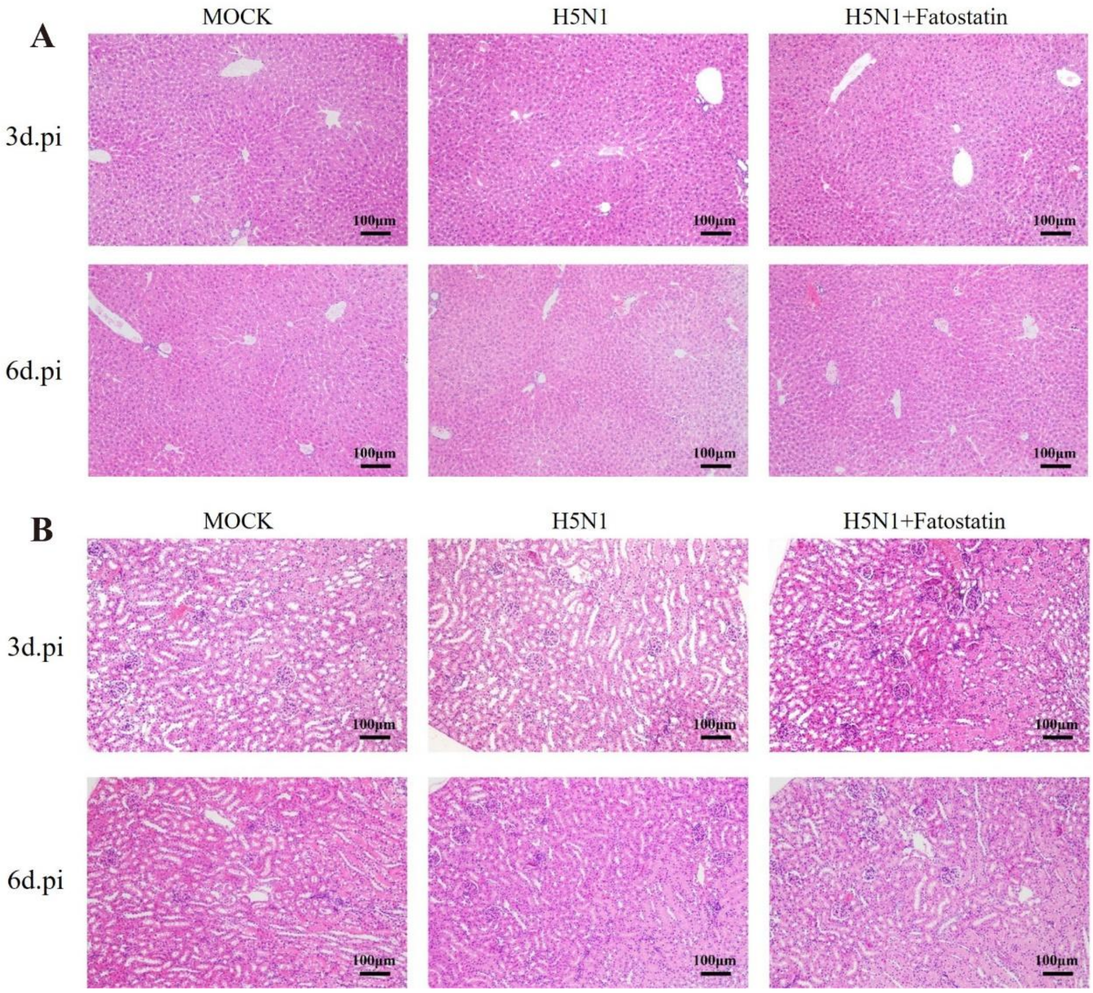

Fig. S4: The histopathologic changes of organ tissues in mice after treatment with fatostatin and infection with H5N1 virus. The histopathologic changes of (A) liver and (B) kidney tissues in mice at day 3 and 6 post-infection were assessed by H&E.

Table S1. Blood lipid test results

Case Number	Test Data	Procedure	Test Result	Unit	Reference range	High/Low
1	2017/01/03	TCHO	5.17	mmol/L	<5.18	
	2017/01/03	TG	1.74	mmol/L	<1.7	H
	2017/01/03	HDL-C	1.14	mmol/L	>1.04	
	2017/01/03	LDL-C	3.85	mmol/L	<3.64	H
2	2017/02/01	TCHO	3.68	mmol/L	<5.18	
	2017/02/01	TG	0.92	mmol/L	<1.7	
	2017/02/01	HDL-C	1.02	mmol/L	>1.04	L
	2017/02/01	LDL-C	2.04	mmol/L	<3.64	
3	2017/02/12	TCHO	4.37	mmol/L	<5.18	
	2017/02/12	TG	1	mmol/L	<1.7	
	2017/02/12	HDL-C	0.92	mmol/L	>1.04	L
	2017/02/12	LDL-C	2.65	mmol/L	<3.64	
4	2017/06/29	TCHO	3.87	mmol/L	<5.18	
	2017/06/29	TG	0.85	mmol/L	<1.7	
	2017/06/29	HDL-C	1.34	mmol/L	>1.04	
	2017/06/29	LDL-C	1.7	mmol/L	<3.64	
5	2017/12/18	TCHO	3.52	mmol/L	<5.18	
	2017/12/18	TG	0.67	mmol/L	<1.7	
	2017/12/18	HDL-C	1.31	mmol/L	>1.04	
	2017/12/18	LDL-C	2.09	mmol/L	<3.64	
6	2017/12/16	TCHO	4.78	mmol/L	<5.18	
	2017/12/16	TG	0.91	mmol/L	<1.7	
	2017/12/16	HDL-C	1.52	mmol/L	>1.04	
	2017/12/16	LDL-C	3.01	mmol/L	<3.64	
7	2017/12/27	TCHO	3.42	mmol/L	<5.18	
	2017/12/27	TG	0.82	mmol/L	<1.7	
	2017/12/27	HDL-C	0.74	mmol/L	>1.04	L
	2017/12/27	LDL-C	2.43	mmol/L	<3.64	
8	2017/12/29	TCHO	3.33	mmol/L	<5.18	
	2017/12/29	TG	0.71	mmol/L	<1.7	
	2017/12/29	HDL-C	0.98	mmol/L	>1.04	L
	2017/12/29	LDL-C	2.04	mmol/L	<3.64	
9	2018/01/03	TCHO	4.24	mmol/L	<5.18	
	2018/01/03	TG	0.77	mmol/L	<1.7	
	2018/01/03	HDL-C	1.32	mmol/L	>1.04	
	2018/01/03	LDL-C	2.6	mmol/L	<3.64	
10	2018/01/09	TCHO	5.52	mmol/L	<5.18	H
	2018/01/09	TG	12.25	mmol/L	<1.7	H
	2018/01/09	HDL-C	0.6	mmol/L	>1.04	L
	2018/01/09	LDL-C	4.66	mmol/L	<3.64	H
11	2018/01/12	TCHO	3.83	mmol/L	<5.18	
	2018/01/12	TG	1.48	mmol/L	<1.7	
	2018/01/12	HDL-C	0.68	mmol/L	>1.04	L
	2018/01/12	LDL-C	2.51	mmol/L	<3.64	
12	2018/01/15	TCHO	2.77	mmol/L	<5.18	L
	2018/01/15	TG	0.64	mmol/L	<1.7	
	2018/01/15	HDL-C	1.4	mmol/L	>1.04	
	2018/01/15	LDL-C	1.03	mmol/L	<3.64	
13	2018/01/19	TCHO	2.01	mmol/L	<5.18	L
	2018/01/19	TG	0.44	mmol/L	<1.7	L
	2018/01/19	HDL-C	0.57	mmol/L	>1.04	L
	2018/01/19	LDL-C	1.32	mmol/L	<3.64	
14	2018/01/16	TCHO	3.91	mmol/L	<5.18	
	2018/01/16	TG	1.52	mmol/L	<1.7	

	2018/01/16	HDL-C	0.84	mmol/L	>1.04	L
	2018/01/16	LDL-C	2.61	mmol/L	<3.64	
15	2018/01/20	TCHO	5.81	mmol/L	<5.18	H
	2018/01/20	TG	1.27	mmol/L	<1.7	
	2018/01/20	HDL-C	1.33	mmol/L	>1.04	
	2018/01/20	LDL-C	3.81	mmol/L	<3.64	H
16	2018/01/25	TCHO	3.81	mmol/L	<5.18	
	2018/01/25	TG	0.8	mmol/L	<1.7	
	2018/01/25	HDL-C	1.21	mmol/L	≥1.04	
	2018/01/25	LDL-C	2.42	mmol/L	<3.64	
17	2018/01/27	TCHO	3.77	mmol/L	<5.18	
	2018/01/27	TG	0.71	mmol/L	<1.7	
	2018/01/27	HDL-C	1.05	mmol/L	≥1.04	
	2018/01/27	LDL-C	2.56	mmol/L	<3.64	
18	2018/02/02	TCHO	3.47	mmol/L	<5.18	
	2018/02/02	TG	0.51	mmol/L	<1.7	
	2018/02/02	HDL-C	1.43	mmol/L	≥1.04	
	2018/02/02	LDL-C	1.81	mmol/L	<3.64	
19	2018/02/06	TCHO	4.45	mmol/L	<5.18	
	2018/02/06	TG	0.92	mmol/L	<1.7	
	2018/02/06	HDL-C	1.16	mmol/L	>1.04	
	2018/02/06	LDL-C	2.98	mmol/L	<3.64	
20	2018/01/30	TCHO	2.38	mmol/L	<5.18	L
	2018/01/30	TG	1.68	mmol/L	<1.7	
	2018/01/30	HDL-C	0.49	mmol/L	>1.04	L
	2018/01/30	LDL-C	1.5	mmol/L	<3.64	
21	2018/02/01	TCHO	2.94	mmol/L	<5.18	
	2018/02/01	TG	0.81	mmol/L	<1.7	
	2018/02/01	HDL-C	1.3	mmol/L	≥1.04	
	2018/02/01	LDL-C	1.36	mmol/L	<3.64	
22	2018/02/14	TCHO	4.68	mmol/L	<5.18	
	2018/02/14	TG	1.16	mmol/L	<1.7	
	2018/02/14	HDL-C	1.56	mmol/L	>1.04	
	2018/02/14	LDL-C	2.44	mmol/L	<3.64	
23	2018/02/14	TCHO	3.02	mmol/L	<5.18	
	2018/02/14	TG	0.9	mmol/L	<1.7	
	2018/02/14	HDL-C	0.92	mmol/L	>1.04	L
	2018/02/14	LDL-C	1.65	mmol/L	<3.64	
24	2018/02/09	TCHO	2.37	mmol/L	<5.18	L
	2018/02/09	TG	1.19	mmol/L	<1.7	
	2018/02/09	HDL-C	0.75	mmol/L	>1.04	L
	2018/02/09	LDL-C	1.35	mmol/L	<3.64	
25	2018/02/22	TCHO	3.39	mmol/L	<5.18	
	2018/02/22	TG	1.29	mmol/L	<1.7	
	2018/02/22	HDL-C	0.96	mmol/L	>1.04	L
	2018/02/22	LDL-C	2.2	mmol/L	<3.64	
26	2018/03/07	TCHO	7.37	mmol/L	<5.18	H
	2018/03/07	TG	1	mmol/L	<1.7	
	2018/03/07	HDL-C	3.2	mmol/L	>1.04	
	2018/03/07	LDL-C	3.37	mmol/L	<3.64	
27	2018/03/13	TCHO	3.88	mmol/L	<5.18	
	2018/03/13	TG	1.58	mmol/L	<1.7	
	2018/03/13	HDL-C	1.27	mmol/L	>1.04	
	2018/03/13	LDL-C	2.07	mmol/L	<3.64	
28	2018/03/21	TCHO	2.16	mmol/L	<5.18	L
	2018/03/21	TG	0.93	mmol/L	<1.7	

	2018/03/21	HDL-C	0.63	mmol/L	>1.04	L
	2018/03/21	LDL-C	1.14	mmol/L	<3.64	
29	2018/03/19	TCHO	5.58	mmol/L	<5.18	H
	2018/03/19	TG	1.82	mmol/L	<1.7	H
	2018/03/19	HDL-C	1.05	mmol/L	>1.04	
	2018/03/19	LDL-C	4.23	mmol/L	<3.64	H
30	2018/04/19	TCHO	4.6	mmol/L	<5.18	
	2018/04/19	TG	1.45	mmol/L	<1.7	
	2018/04/19	HDL-C	1.08	mmol/L	>1.04	
	2018/04/19	LDL-C	2.9	mmol/L	<3.64	
31	2018/11/23	TCHO	3.81	mmol/L	<5.18	
	2018/11/23	TG	3.03	mmol/L	<1.7	H
	2018/11/23	HDL-C	0.65	mmol/L	>1.04	L
	2018/11/23	LDL-C	2.06	mmol/L	<3.64	
32	2018/12/16	TCHO	8.9	mmol/L	<5.18	H
	2018/12/16	TG	5.63	mmol/L	<1.7	H
	2018/12/16	HDL-C	1.1	mmol/L	>1.04	
	2018/12/16	LDL-C	5.39	mmol/L	<3.64	H
33	2018/12/28	TCHO	2.86	mmol/L	<5.18	
	2018/12/28	TG	1.44	mmol/L	<1.7	
	2018/12/28	HDL-C	0.77	mmol/L	>1.04	L
	2018/12/28	LDL-C	1.47	mmol/L	<3.64	
34	2019/01/03	TCHO	2.93	mmol/L	<5.18	
	2019/01/03	TG	1.06	mmol/L	<1.7	
	2019/01/03	HDL-C	0.93	mmol/L	>1.04	L
	2019/01/03	LDL-C	1.69	mmol/L	<3.64	
35	2019/01/03	TCHO	3.19	mmol/L	<5.18	
	2019/01/03	TG	0.66	mmol/L	<1.7	
	2019/01/03	HDL-C	0.84	mmol/L	>1.04	L
	2019/01/03	LDL-C	2.09	mmol/L	<3.64	
36	2019/01/08	TCHO	2.93	mmol/L	<5.18	
	2019/01/08	TG	0.98	mmol/L	<1.7	
	2019/01/08	HDL-C	0.87	mmol/L	>1.04	L
	2019/01/08	LDL-C	1.75	mmol/L	<3.64	
37	2019/01/03	TCHO	3.75	mmol/L	<5.18	
	2019/01/03	TG	1.09	mmol/L	<1.7	
	2019/01/03	HDL-C	0.93	mmol/L	≥1.04	L
	2019/01/03	LDL-C	2.67	mmol/L	<3.64	
38	2019/01/05	TCHO	3.16	mmol/L	<5.18	
	2019/01/05	TG	1.24	mmol/L	<1.7	
	2019/01/05	HDL-C	0.81	mmol/L	≥1.04	L
	2019/01/05	LDL-C	1.88	mmol/L	<3.64	
39	2019/01/09	TCHO	1.85	mmol/L	<5.18	
	2019/01/09	TG	0.96	mmol/L	<1.7	
	2019/01/09	HDL-C	0.3	mmol/L	≥1.04	L
	2019/01/09	LDL-C	1.19	mmol/L	<3.64	
40	2019/01/09	TCHO	2.65	mmol/L	<5.18	
	2019/01/09	TG	0.66	mmol/L	<1.7	
	2019/01/09	HDL-C	1.1	mmol/L	>1.04	
	2019/01/09	LDL-C	1.27	mmol/L	<3.64	
41	2019/01/12	TCHO	4.02	mmol/L	<5.18	
	2019/01/12	TG	2.07	mmol/L	<1.7	H
	2019/01/12	HDL-C	0.8	mmol/L	>1.04	L
	2019/01/12	LDL-C	2.61	mmol/L	<3.64	
42	2019/01/10	TCHO	3.18	mmol/L	<5.18	
	2019/01/10	TG	2.12	mmol/L	<1.7	H

	2019/01/10	HDL-C	0.71	mmol/L	>1.04	L
	2019/01/10	LDL-C	1.98	mmol/L	<3.64	
43	2019/01/15	TCHO	2.9	mmol/L	<5.18	
	2019/01/15	TG	1.53	mmol/L	<1.7	
	2019/01/15	HDL-C	0.84	mmol/L	>1.04	L
	2019/01/15	LDL-C	1.53	mmol/L	<3.64	
44	2019/01/11	TCHO	3.37	mmol/L	<5.18	
	2019/01/11	TG	1.3	mmol/L	<1.7	
	2019/01/11	HDL-C	1.03	mmol/L	>1.04	L
	2019/01/11	LDL-C	2.03	mmol/L	<3.64	
45	2019/01/11	TCHO	3.87	mmol/L	<5.18	
	2019/01/11	TG	1.34	mmol/L	<1.7	
	2019/01/11	HDL-C	1.26	mmol/L	>1.04	
	2019/01/11	LDL-C	2.27	mmol/L	<3.64	
46	2019/01/10	TCHO	3.58	mmol/L	<5.18	
	2019/01/10	TG	0.78	mmol/L	<1.7	
	2019/01/10	HDL-C	1.14	mmol/L	≥1.04	
	2019/01/10	LDL-C	2.3	mmol/L	<3.64	
47	2019/01/10	TCHO	3.32	mmol/L	<5.18	
	2019/01/10	TG	0.78	mmol/L	<1.7	
	2019/01/10	HDL-C	1.22	mmol/L	≥1.04	
	2019/01/10	LDL-C	1.81	mmol/L	<3.64	
48	2019/01/15	TCHO	3.9	mmol/L	<5.18	
	2019/01/15	TG	0.87	mmol/L	<1.7	
	2019/01/15	HDL-C	1.98	mmol/L	≥1.04	
	2019/01/15	LDL-C	1.21	mmol/L	<3.64	
49	2019/01/11	TCHO	5.31	mmol/L	<5.18	H
	2019/01/11	TG	0.96	mmol/L	<1.7	
	2019/01/11	HDL-C	1.09	mmol/L	>1.04	
	2019/01/11	LDL-C	3.76	mmol/L	<3.64	H
50	2019/01/16	TCHO	3.38	mmol/L	<5.18	
	2019/01/16	TG	0.68	mmol/L	<1.7	
	2019/01/16	HDL-C	1.82	mmol/L	>1.04	
	2019/01/16	LDL-C	1.22	mmol/L	<3.64	
51	2019/01/14	TCHO	5.27	mmol/L	<5.18	H
	2019/01/14	TG	0.67	mmol/L	<1.7	
	2019/01/14	HDL-C	2.02	mmol/L	>1.04	
	2019/01/14	LDL-C	2.79	mmol/L	<3.64	
52	2019/01/16	TCHO	4.3	mmol/L	<5.18	
	2019/01/16	TG	1.47	mmol/L	<1.7	
	2019/01/16	HDL-C	1.21	mmol/L	>1.04	
	2019/01/16	LDL-C	2.58	mmol/L	<3.64	
53	2019/01/19	TCHO	3.92	mmol/L	<5.18	
	2019/01/19	TG	1.02	mmol/L	<1.7	
	2019/01/19	HDL-C	1.02	mmol/L	>1.04	L
	2019/01/19	LDL-C	2.57	mmol/L	<3.64	
54	2019/01/19	TCHO	2.91	mmol/L	<5.18	
	2019/01/19	TG	0.97	mmol/L	<1.7	
	2019/01/19	HDL-C	0.84	mmol/L	>1.04	L
	2019/01/19	LDL-C	1.53	mmol/L	<3.64	
55	2019/01/18	TCHO	4.87	mmol/L	<5.18	
	2019/01/18	TG	1.22	mmol/L	<1.7	
	2019/01/18	HDL-C	1.02	mmol/L	>1.04	L
	2019/01/18	LDL-C	3.63	mmol/L	<3.64	
56	2019/01/23	TCHO	5.47	mmol/L	<5.18	H
	2019/01/23	TG	1.49	mmol/L	<1.7	

	2019/01/23	HDL-C	1.25	mmol/L	>1.04	
	2019/01/23	LDL-C	3.41	mmol/L	<3.64	
57	2019/01/21	TCHO	5.82	mmol/L	<5.18	H
	2019/01/21	TG	0.99	mmol/L	<1.7	
	2019/01/21	HDL-C	1.65	mmol/L	>1.04	
	2019/01/21	LDL-C	3.67	mmol/L	<3.64	H
58	2019/01/23	TCHO	4.48	mmol/L	<5.18	
	2019/01/23	TG	0.81	mmol/L	<1.7	
	2019/01/23	HDL-C	1.43	mmol/L	>1.04	
	2019/01/23	LDL-C	2.69	mmol/L	<3.64	
59	2019/01/24	TCHO	4.95	mmol/L	<5.18	
	2019/01/24	TG	0.53	mmol/L	<1.7	
	2019/01/24	HDL-C	2.11	mmol/L	>1.04	
	2019/01/24	LDL-C	2.29	mmol/L	<3.64	
60	2019/01/25	TCHO	4.32	mmol/L	<5.18	
	2019/01/25	TG	2.49	mmol/L	<1.7	H
	2019/01/25	HDL-C	0.97	mmol/L	>1.04	L
	2019/01/25	LDL-C	2.54	mmol/L	<3.64	
61	2019/01/25	TCHO	2.86	mmol/L	<5.18	
	2019/01/25	TG	0.57	mmol/L	<1.7	
	2019/01/25	HDL-C	1.48	mmol/L	>1.04	
	2019/01/25	LDL-C	1	mmol/L	<3.64	
62	2019/01/29	TCHO	3.92	mmol/L	<5.18	
	2019/01/29	TG	0.94	mmol/L	<1.7	
	2019/01/29	HDL-C	1.48	mmol/L	>1.04	
	2019/01/29	LDL-C	1.99	mmol/L	<3.64	
63	2019/02/09	TCHO	4.09	mmol/L	<5.18	
	2019/02/09	TG	1.17	mmol/L	<1.7	
	2019/02/09	HDL-C	0.99	mmol/L	>1.04	L
	2019/02/09	LDL-C	2.51	mmol/L	<3.64	
64	2019/02/19	TCHO	3	mmol/L	<5.18	
	2019/02/19	TG	0.96	mmol/L	<1.7	
	2019/02/19	HDL-C	1.1	mmol/L	>1.04	
	2019/02/19	LDL-C	1.57	mmol/L	<3.64	
65	2019/03/19	TCHO	3.81	mmol/L	<5.18	
	2019/03/19	TG	1.67	mmol/L	<1.7	
	2019/03/19	HDL-C	1	mmol/L	>1.04	L
	2019/03/19	LDL-C	2.1	mmol/L	<3.64	
66	2019/03/21	TCHO	4.51	mmol/L	<5.18	
	2019/03/21	TG	0.83	mmol/L	<1.7	
	2019/03/21	HDL-C	1.37	mmol/L	>1.04	
	2019/03/21	LDL-C	2.77	mmol/L	<3.64	
67	2019/04/01	TCHO	2.64	mmol/L	<5.18	
	2019/04/01	TG	0.88	mmol/L	<1.7	
	2019/04/01	HDL-C	0.53	mmol/L	>1.04	L
	2019/04/01	LDL-C	1.8	mmol/L	<3.64	
68	2019/04/02	TCHO	2.54	mmol/L	<5.18	
	2019/04/02	TG	0.77	mmol/L	<1.7	
	2019/04/02	HDL-C	0.95	mmol/L	>1.04	L
	2019/04/02	LDL-C	1.31	mmol/L	<3.64	
69	2019/04/09	TCHO	3.16	mmol/L	<5.18	
	2019/04/09	TG	0.67	mmol/L	<1.7	
	2019/04/09	HDL-C	1.45	mmol/L	>1.04	
	2019/04/09	LDL-C	1.25	mmol/L	<3.64	
70	2019/04/12	TCHO	2.56	mmol/L	<5.18	
	2019/04/12	TG	1.38	mmol/L	<1.7	

	2019/04/12	HDL-C	0.26	mmol/L	>1.04	L
	2019/04/12	LDL-C	1.56	mmol/L	<3.64	
71	2019/05/02	TCHO	3.38	mmol/L	<5.18	
	2019/05/02	TG	1.79	mmol/L	<1.7	H
	2019/05/02	HDL-C	0.95	mmol/L	>1.04	L
	2019/05/02	LDL-C	2.26	mmol/L	<3.64	
72	2019/04/30	TCHO	3.41	mmol/L	<5.18	
	2019/04/30	TG	1.51	mmol/L	<1.7	
	2019/04/30	HDL-C	0.61	mmol/L	>1.04	L
	2019/04/30	LDL-C	2.28	mmol/L	<3.64	
73	2019/05/03	TCHO	3.76	mmol/L	<5.18	
	2019/05/03	TG	1.48	mmol/L	<1.7	
	2019/05/03	HDL-C	0.74	mmol/L	>1.04	L
	2019/05/03	LDL-C	2.7	mmol/L	<3.64	
74	2019/05/16	TCHO	3.3	mmol/L	<5.18	
	2019/05/16	TG	0.79	mmol/L	<1.7	
	2019/05/16	HDL-C	1.34	mmol/L	>1.04	
	2019/05/16	LDL-C	1.73	mmol/L	<3.64	
75	2019/05/22	TCHO	3.39	mmol/L	<5.18	
	2019/05/22	TG	0.84	mmol/L	<1.7	
	2019/05/22	HDL-C	0.78	mmol/L	>1.04	L
	2019/05/22	LDL-C	2.56	mmol/L	<3.64	
76	2019/09/24	TCHO	6.1	mmol/L	<5.18	H
	2019/09/24	TG	1.98	mmol/L	<1.7	H
	2019/09/24	HDL-C	1.69	mmol/L	>1.04	
	2019/09/24	LDL-C	3.62	mmol/L	<3.64	
77	2019/11/26	TCHO	2.87	mmol/L	<5.18	
	2019/11/26	TG	0.42	mmol/L	<1.7	
	2019/11/26	HDL-C	0.94	mmol/L	>1.04	L
	2019/11/26	LDL-C	1.69	mmol/L	<3.64	
78	2019/12/04	TCHO	4.71	mmol/L	<5.18	
	2019/12/04	TG	1.33	mmol/L	<1.7	
	2019/12/04	HDL-C	0.63	mmol/L	>1.04	L
	2019/12/04	LDL-C	3.56	mmol/L	<3.64	
79	2019/12/24	TCHO	4.35	mmol/L	<5.18	
	2019/12/24	TG	2.1	mmol/L	<1.7	H
	2019/12/24	HDL-C	0.74	mmol/L	>1.04	L
	2019/12/24	LDL-C	3.11	mmol/L	<3.64	
80	2019/11/23	TCHO	3.33	mmol/L	<5.18	
	2019/11/23	TG	0.73	mmol/L	<1.7	
	2019/11/23	HDL-C	0.6	mmol/L	>1.04	L
	2019/11/23	LDL-C	2.55	mmol/L	<3.64	
81	2019/12/27	TCHO	3.36	mmol/L	<5.18	
	2019/12/27	TG	1.29	mmol/L	<1.7	
	2019/12/27	HDL-C	0.85	mmol/L	>1.04	L
	2019/12/27	LDL-C	2.06	mmol/L	<3.64	
82	2019/12/24	TCHO	6.31	mmol/L	<5.18	H
	2019/12/24	TG	8.23	mmol/L	<1.7	H
	2019/12/24	HDL-C	1.15	mmol/L	>1.04	
	2019/12/24	LDL-C	2.34	mmol/L	<3.64	
83	2019/12/25	TCHO	4.69	mmol/L	<5.18	
	2019/12/25	TG	1.25	mmol/L	<1.7	
	2019/12/25	HDL-C	1.16	mmol/L	>1.04	
	2019/12/25	LDL-C	3.15	mmol/L	<3.64	
84	2019/12/26	TCHO	3.24	mmol/L	<5.18	
	2019/12/26	TG	1.19	mmol/L	<1.7	

	2019/12/26	HDL-C	0.97	mmol/L	>1.04	L
	2019/12/26	LDL-C	1.84	mmol/L	<3.64	
85	2019/12/31	TCHO	3.42	mmol/L	<5.18	
	2019/12/31	TG	1.42	mmol/L	<1.7	
	2019/12/31	HDL-C	0.52	mmol/L	>1.04	L
	2019/12/31	LDL-C	2.41	mmol/L	<3.64	
86	2019/12/31	TCHO	4.79	mmol/L	<5.18	
	2019/12/31	TG	0.7	mmol/L	<1.7	
	2019/12/31	HDL-C	1.31	mmol/L	>1.04	
	2019/12/31	LDL-C	3.02	mmol/L	<3.64	
87	2020/01/04	TCHO	2.93	mmol/L	<5.18	
	2020/01/04	TG	0.6	mmol/L	<1.7	
	2020/01/04	HDL-C	1.3	mmol/L	>1.04	
	2020/01/04	LDL-C	1.52	mmol/L	<3.64	
88	2020/01/04	TCHO	4.5	mmol/L	<5.18	
	2020/01/04	TG	1.73	mmol/L	<1.7	H
	2020/01/04	HDL-C	0.72	mmol/L	>1.04	L
	2020/01/04	LDL-C	3.35	mmol/L	<3.64	
89	2019/12/30	TCHO	3.07	mmol/L	<5.18	
	2019/12/30	TG	1.04	mmol/L	<1.7	
	2019/12/30	HDL-C	1.24	mmol/L	>1.04	
	2019/12/30	LDL-C	1.4	mmol/L	<3.64	
90	2020/01/02	TCHO	4.62	mmol/L	<5.18	
	2020/01/02	TG	1.38	mmol/L	<1.7	
	2020/01/02	HDL-C	1.25	mmol/L	>1.04	
	2020/01/02	LDL-C	2.8	mmol/L	<3.64	
91	2020/01/04	TCHO	2.92	mmol/L	<5.18	
	2020/01/04	TG	0.87	mmol/L	<1.7	
	2020/01/04	HDL-C	1.08	mmol/L	>1.04	
	2020/01/04	LDL-C	1.49	mmol/L	<3.64	
92	2020/01/07	TCHO	1.24	mmol/L	<5.18	
	2020/01/07	TG	0.46	mmol/L	<1.7	
	2020/01/07	HDL-C	0.43	mmol/L	>1.04	L
	2020/01/07	LDL-C	0.75	mmol/L	<3.64	
93	2020/01/04	TCHO	4.86	mmol/L	<5.18	
	2020/01/04	TG	0.47	mmol/L	<1.7	
	2020/01/04	HDL-C	1.5	mmol/L	>1.04	
	2020/01/04	LDL-C	3.28	mmol/L	<3.64	
94	2020/01/09	TCHO	3.89	mmol/L	<5.18	
	2020/01/09	TG	0.52	mmol/L	<1.7	
	2020/01/09	HDL-C	1.42	mmol/L	>1.04	
	2020/01/09	LDL-C	2.37	mmol/L	<3.64	
95	2020/01/09	TCHO	2.42	mmol/L	<5.18	
	2020/01/09	TG	1.29	mmol/L	<1.7	
	2020/01/09	HDL-C	0.82	mmol/L	>1.04	L
	2020/01/09	LDL-C	1.38	mmol/L	<3.64	
96	2020/01/13	TCHO	3.4	mmol/L	<5.18	
	2020/01/13	TG	0.85	mmol/L	<1.7	
	2020/01/13	HDL-C	1.08	mmol/L	≥1.04	
	2020/01/13	LDL-C	1.85	mmol/L	<3.64	
97	2020/01/14	TCHO	7.86	mmol/L	<5.18	H
	2020/01/14	TG	3.87	mmol/L	<1.7	H
	2020/01/14	HDL-C	1.5	mmol/L	>1.04	
	2020/01/14	LDL-C	4.96	mmol/L	<3.64	H
98	2020/01/15	TCHO	3.14	mmol/L	<5.18	
	2020/01/15	TG	1.02	mmol/L	<1.7	

	2020/01/15	HDL-C	0.97	mmol/L	>1.04	L
	2020/01/15	LDL-C	1.83	mmol/L	<3.64	
99	2020/12/09	TCHO	3.29	mmol/L	<5.18	
	2020/12/09	TG	1.59	mmol/L	<1.7	
	2020/12/09	HDL-C	0.73	mmol/L	≥1.04	L
	2020/12/09	LDL-C	1.95	mmol/L	<3.64	
100	2020/12/21	TCHO	3.85	mmol/L	<5.18	
	2020/12/21	TG	1.13	mmol/L	<1.7	
	2020/12/21	HDL-C	1.24	mmol/L	>1.04	
	2020/12/21	LDL-C	2.28	mmol/L	<3.64	
101	2021/04/09	TCHO	5.2	mmol/L	<5.18	H
	2021/04/09	TG	1.09	mmol/L	<1.7	
	2021/04/09	HDL-C	0.82	mmol/L	>1.04	L
	2021/04/09	LDL-C	3.71	mmol/L	<3.64	H
102	2021/09/15	TCHO	2.88	mmol/L	<5.18	
	2021/09/15	TG	1.11	mmol/L	<1.7	
	2021/09/15	HDL-C	0.69	mmol/L	>1.04	L
	2021/09/15	LDL-C	1.75	mmol/L	<3.64	
103	2021/09/17	TCHO	6.14	mmol/L	<5.18	H
	2021/09/17	TG	1.41	mmol/L	<1.7	
	2021/09/17	HDL-C	0.94	mmol/L	>1.04	L
	2021/09/17	LDL-C	4.45	mmol/L	<3.64	H
104	2021/11/04	TCHO	5.28	mmol/L	<5.18	H
	2021/11/04	TG	0.75	mmol/L	<1.7	
	2021/11/04	HDL-C	1.54	mmol/L	>1.04	
	2021/11/04	LDL-C	2.95	mmol/L	<3.64	
105	2021/12/07	TCHO	3.96	mmol/L	<5.18	
	2021/12/07	TG	0.48	mmol/L	<1.7	
	2021/12/07	HDL-C	0.72	mmol/L	>1.04	L
	2021/12/07	LDL-C	2.72	mmol/L	<3.64	
106	2021/12/16	TCHO	2.65	mmol/L	<5.18	
	2021/12/16	TG	0.48	mmol/L	<1.7	
	2021/12/16	HDL-C	0.9	mmol/L	>1.04	L
	2021/12/16	LDL-C	1.27	mmol/L	<3.64	
107	2022/07/19	TCHO	2.83	mmol/L	<5.18	
	2022/07/19	TG	0.7	mmol/L	<1.7	
	2022/07/19	HDL-C	1.12	mmol/L	>1.04	
	2022/07/19	LDL-C	1.14	mmol/L	<3.64	
108	2022/07/29	TCHO	3.37	mmol/L	<5.18	
	2022/07/29	TG	0.98	mmol/L	<1.7	
	2022/07/29	HDL-C	1.14	mmol/L	>1.04	
	2022/07/29	LDL-C	1.58	mmol/L	<3.64	
109	2022/08/13	TCHO	3.41	mmol/L	<5.18	
	2022/08/13	TG	1.18	mmol/L	<1.7	
	2022/08/13	HDL-C	0.69	mmol/L	>1.04	L
	2022/08/13	LDL-C	2.11	mmol/L	<3.64	
110	2022/07/02	TCHO	2.23	mmol/L	<5.18	
	2022/07/02	TG	1.11	mmol/L	<1.7	
	2022/07/02	HDL-C	0.86	mmol/L	>1.04	L
	2022/07/02	LDL-C	1.03	mmol/L	<3.64	
111	2022/08/27	TCHO	4.6	mmol/L	<5.18	
	2022/08/27	TG	0.79	mmol/L	<1.7	
	2022/08/27	HDL-C	0.79	mmol/L	>1.04	L
	2022/08/27	LDL-C	3.18	mmol/L	<3.64	
112	2022/09/09	TCHO	3.52	mmol/L	<5.18	
	2022/09/09	TG	1.42	mmol/L	<1.7	

	2022/09/09	HDL-C	0.94	mmol/L	>1.04	L
	2022/09/09	LDL-C	1.97	mmol/L	<3.64	
113	2022/03/10	TCHO	5.63	mmol/L	<5.18	H
	2022/03/10	TG	1.09	mmol/L	<1.7	
	2022/03/10	HDL-C	1.64	mmol/L	>1.04	
	2022/03/10	LDL-C	3.04	mmol/L	<3.64	
114	2022/04/23	TCHO	1.81	mmol/L	<5.18	
	2022/04/23	TG	1.16	mmol/L	<1.7	
	2022/04/23	HDL-C	0.41	mmol/L	>1.04	L
	2022/04/23	LDL-C	0.67	mmol/L	<3.64	
115	2022/07/25	TCHO	2.92	mmol/L	<5.18	
	2022/07/25	TG	0.71	mmol/L	<1.7	
	2022/07/25	HDL-C	0.56	mmol/L	>1.04	L
	2022/07/25	LDL-C	1.89	mmol/L	<3.64	
116	2022/08/25	TCHO	2.13	mmol/L	<5.18	
	2022/08/25	TG	1.2	mmol/L	<1.7	
	2022/08/25	HDL-C	0.69	mmol/L	>1.04	L
	2022/08/25	LDL-C	1.05	mmol/L	<3.64	
117	2022/09/16	TCHO	3.17	mmol/L	<5.18	
	2022/09/16	TG	1.48	mmol/L	<1.7	
	2022/09/16	HDL-C	0.55	mmol/L	>1.04	L
	2022/09/16	LDL-C	2.2	mmol/L	<3.64	
118	2022/07/04	TCHO	3.73	mmol/L	<5.18	
	2022/07/04	TG	0.65	mmol/L	<1.7	
	2022/07/04	HDL-C	1.11	mmol/L	>1.04	
	2022/07/04	LDL-C	2.22	mmol/L	<3.64	
119	2022/02/22	TCHO	4.21	mmol/L	<5.18	
	2022/02/22	TG	0.52	mmol/L	<1.7	
	2022/02/22	HDL-C	1	mmol/L	>1.04	L
	2022/02/22	LDL-C	3.01	mmol/L	<3.64	
120	2022/02/09	TCHO	4.11	mmol/L	<5.18	
	2022/02/09	TG	1.29	mmol/L	<1.7	
	2022/02/09	HDL-C	1.16	mmol/L	≥1.04	
	2022/02/09	LDL-C	2.01	mmol/L	0-3.4	
121	2022/06/23	TCHO	1.94	mmol/L	<5.18	
	2022/06/23	TG	0.72	mmol/L	<1.7	
	2022/06/23	HDL-C	0.53	mmol/L	>1.04	L
	2022/06/23	LDL-C	1.03	mmol/L	<3.64	
122	2022/09/06	TCHO	3.8	mmol/L	<5.18	
	2022/09/06	TG	0.85	mmol/L	<1.7	
	2022/09/06	HDL-C	0.84	mmol/L	>1.04	L
	2022/09/06	LDL-C	2.48	mmol/L	<3.64	
123	2022/08/06	TCHO	6.47	mmol/L	<1.7	H
	2022/08/06	TG	1.76	mmol/L	<5.18	H
	2022/08/06	HDL-C	1.22	mmol/L	<1.7	
	2022/08/06	LDL-C	4.26	mmol/L	>1.04	H
124	2022/01/08	TCHO	4.14	mmol/L	<5.18	
	2022/01/08	TG	0.92	mmol/L	<1.7	
	2022/01/08	HDL-C	1.08	mmol/L	>1.04	
	2022/01/08	LDL-C	2.42	mmol/L	<3.64	
125	2022/01/07	TCHO	4	mmol/L	<5.18	
	2022/01/07	TG	0.8	mmol/L	<1.7	
	2022/01/07	HDL-C	0.94	mmol/L	>1.04	L
	2022/01/07	LDL-C	2.36	mmol/L	<3.64	
126	2022/01/06	TCHO	3.99	mmol/L	<5.18	
	2022/01/06	TG	0.41	mmol/L	<1.7	

	2022/01/06	HDL-C	1.6		mmol/L	>1.04	
	2022/01/06	LDL-C	1.83		mmol/L	<3.64	
127	2022/01/21	TCHO	4.68		mmol/L	<5.18	
	2022/01/21	TG	1.09		mmol/L	<1.7	
	2022/01/21	HDL-C	1.68		mmol/L	>1.04	
	2022/01/21	LDL-C	2.06		mmol/L	<3.64	
128	2023/09/07	TCHO	7.21		mmol/L	<5.18	H
	2023/09/07	TG	3.43		mmol/L	<1.7	H
	2023/09/07	HDL-C	1.5		mmol/L	>1.04	
	2023/09/07	LDL-C	4.51		mmol/L	<3.64	H
129	2023/11/20	TCHO	4.11		mmol/L	<5.18	
	2023/11/20	TG	1.67		mmol/L	<1.7	
	2023/11/20	HDL-C	1.02		mmol/L	>1.04	L
	2023/11/20	LDL-C	2.17		mmol/L	<3.64	
130	2023/06/09	TCHO	2.35		mmol/L	<5.18	
	2023/06/09	TG	1		mmol/L	<1.7	
	2023/06/09	HDL-C	0.71		mmol/L	>1.04	L
	2023/06/09	LDL-C	1.4		mmol/L	<3.64	
131	2023/05/12	TCHO	3.6		mmol/L	<5.18	
	2023/05/12	TG	0.99		mmol/L	<1.7	
	2023/05/12	HDL-C	1.16		mmol/L	≥1.04	
	2023/05/12	LDL-C	2.16		mmol/L	<3.64	
132	2023/03/02	TCHO	4.52		mmol/L	<5.18	
	2023/03/02	TG	1.25		mmol/L	<1.7	
	2023/03/02	HDL-C	1.31		mmol/L	≥1.04	
	2023/03/02	LDL-C	2.36		mmol/L	<3.64	
133	2023/02/19	TCHO	5.19		mmol/L	<5.18	H
	2023/02/19	TG	1.4		mmol/L	<1.7	
	2023/02/19	HDL-C	1.36		mmol/L	>1.04	
	2023/02/19	LDL-C	3.02		mmol/L	<3.64	
134	2023/02/08	TCHO	4.45		mmol/L	<5.18	
	2023/02/08	TG	0.9		mmol/L	<1.7	
	2023/02/08	HDL-C	1.21		mmol/L	≥1.04	
	2023/02/08	LDL-C	2.58		mmol/L	<3.64	
135	2023/02/10	TCHO	3.4		mmol/L	<5.18	
	2023/02/10	TG	1.86		mmol/L	<1.7	H
	2023/02/10	HDL-C	0.63		mmol/L	≥1.04	L
	2023/02/10	LDL-C	2.11		mmol/L	<3.64	
136	2023/03/24	TCHO	2.3		mmol/L	<5.18	
	2023/03/24	TG	0.83		mmol/L	<1.7	
	2023/03/24	HDL-C	0.56		mmol/L	>1.04	L
	2023/03/24	LDL-C	1.53		mmol/L	<3.64	
137	2023/05/14	TCHO	3.32		mmol/L	<5.18	
	2023/05/14	TG		1.59	mmol/L	<1.7	
	2023/05/14	HDL-C		1.09	mmol/L	≥1.04	
	2023/05/14	LDL-C	1.35		mmol/L	<3.64	
138	2023/06/17	TCHO	2.14		mmol/L	<5.18	
	2023/06/17	TG	1.06		mmol/L	<1.7	
	2023/06/17	HDL-C	0.54		mmol/L	≥1.04	L
	2023/06/17	LDL-C	1.33		mmol/L	<3.64	
139	2023/02/27	TCHO	3.72		mmol/L	<5.18	
	2023/02/27	TG	1.1		mmol/L	<1.7	
	2023/02/27	HDL-C	1.37		mmol/L	≥1.04	
	2023/02/27	LDL-C	1.63		mmol/L	<3.64	
140	2023/03/23	TCHO	2.54		mmol/L	<5.18	
	2023/03/23	TG	1.06		mmol/L	<1.7	

	2023/03/23	HDL-C	0.66	mmol/L	≥1.04	L
	2023/03/23	LDL-C	1.55	mmol/L	<3.64	
141	2023/05/27	TCHO	4.87	mmol/L	<5.18	
	2023/05/27	TG	0.8	mmol/L	<1.7	
	2023/05/27	HDL-C	1.26	mmol/L	≥1.04	
	2023/05/27	LDL-C	2.98	mmol/L	<3.64	
142	2023/06/06	TCHO	1.92	mmol/L	<5.18	
	2023/06/06	TG	0.82	mmol/L	<1.7	
	2023/06/06	HDL-C	0.39	mmol/L	≥1.04	L
	2023/06/06	LDL-C	1.29	mmol/L	<3.64	
143	2023/06/01	TCHO	4.12	mmol/L	<5.18	
	2023/06/01	TG	0.7	mmol/L	<1.7	
	2023/06/01	HDL-C	1.41	mmol/L	≥1.04	
	2023/06/01	LDL-C	2.18	mmol/L	<3.64	
144	2023/03/30	TCHO	4.44	mmol/L	<5.18	
	2023/03/30	TG	2.73	mmol/L	<1.7	H
	2023/03/30	HDL-C	0.86	mmol/L	≥1.04	L
	2023/03/30	LDL-C	2.62	mmol/L	<3.64	
145	2023/06/08	TCHO	3.14	mmol/L	<5.18	
	2023/06/08	TG	1.31	mmol/L	<1.7	
	2023/06/08	HDL-C	1.02	mmol/L	≥1.04	L
	2023/06/08	LDL-C	1.33	mmol/L	<3.64	
146	2023/03/01	TCHO	7.65	mmol/L	<5.18	H
	2023/03/01	TG	2.72	mmol/L	<1.7	H
	2023/03/01	HDL-C	1.04	mmol/L	≥1.04	
	2023/03/01	LDL-C	5.03	mmol/L	<3.64	H
147	2023/04/11	TCHO	3.12	mmol/L	<5.18	
	2023/04/11	TG	1.8	mmol/L	<1.7	H
	2023/04/11	HDL-C	0.58	mmol/L	≥1.04	L
	2023/04/11	LDL-C	1.84	mmol/L	<3.64	
148	2023/03/06	TCHO	3.72	mmol/L	<5.18	
	2023/03/06	TG	1.69	mmol/L	<1.7	
	2023/03/06	HDL-C	0.95	mmol/L	>1.04	L
	2023/03/06	LDL-C	2.25	mmol/L	<3.64	
149	2024/03/25	TCHO	2.8	mmol/L	<5.18	
	2024/03/25	TG	1.27	mmol/L	<1.7	
	2024/03/25	HDL-C	0.79	mmol/L	≥1.04	L
	2024/03/25	LDL-C	1.56	mmol/L	<3.64	
150	2024/11/15	TCHO	1.84	mmol/L	<5.18	
	2024/11/15	TG	1.06	mmol/L	<1.7	
	2024/11/15	HDL-C	0.46	mmol/L	≥1.04	L
	2024/11/15	LDL-C	0.86	mmol/L	<3.64	
151	2024/01/24	TCHO	3.19	mmol/L		
	2024/01/24	TG	0.95	mmol/L		
	2024/01/24	HDL-C	0.8	mmol/L		L
	2024/01/24	LDL-C	1.94	mmol/L		
152	2024/03/10	TCHO	3.45	mmol/L		
	2024/03/10	TG	1.33	mmol/L		
	2024/03/10	HDL-C	0.7	mmol/L		L
	2024/03/10	LDL-C	2.21	mmol/L		
153	2024/03/29	TCHO	3.83	mmol/L		
	2024/03/29	TG	0.92	mmol/L		
	2024/03/29	HDL-C	0.78	mmol/L		L
	2024/03/29	LDL-C	2.61	mmol/L		
154	2024/04/13	TCHO	3.68	mmol/L		
	2024/04/13	TG	1.19	mmol/L		

	2024/04/13	HDL-C	0.93	mmol/L	L
	2024/04/13	LDL-C	2.17	mmol/L	
155	2024/06/04	TCHO	5.73	mmol/L	H
	2024/06/04	TG	1.59	mmol/L	
	2024/06/04	HDL-C	1.25	mmol/L	
	2024/06/04	LDL-C	3.42	mmol/L	
156	2024/06/28	TCHO	3.7	mmol/L	
	2024/06/28	TG	1.83	mmol/L	H
	2024/06/28	HDL-C	0.84	mmol/L	L
	2024/06/28	LDL-C	2.31	mmol/L	
157	2024/08/04	TCHO	4.3	mmol/L	
	2024/08/04	TG	1.34	mmol/L	
	2024/08/04	HDL-C	1.12	mmol/L	
	2024/08/04	LDL-C	2.39	mmol/L	
158	2024/12/19	TCHO	4.16	mmol/L	
	2024/12/19	TG	0.94	mmol/L	
	2024/12/19	HDL-C	1	mmol/L	L
	2024/12/19	LDL-C	2.69	mmol/L	
159	2024/03/17	TCHO	3.51	mmol/L	
	2024/03/17	TG	2.16	mmol/L	H
	2024/03/17	HDL-C	1.02	mmol/L	L
	2024/03/17	LDL-C	1.74	mmol/L	
160	2024/02/19	TCHO	4.11	mmol/L	
	2024/02/19	TG	2.86	mmol/L	H
	2024/02/19	HDL-C	0.69	mmol/L	L
	2024/02/19	LDL-C	2.13	mmol/L	
161	2024/04/09	TCHO	7.14	mmol/L	H
	2024/04/09	TG	4.97	mmol/L	H
	2024/04/09	HDL-C	1.17	mmol/L	
	2024/04/09	LDL-C	4.14	mmol/L	H
162	2024/06/26	TCHO	4.43	mmol/L	
	2024/06/26	TG	1.89	mmol/L	H
	2024/06/26	HDL-C	0.91	mmol/L	L
	2024/06/26	LDL-C	3.19	mmol/L	
163	2024/11/18	TCHO	5	mmol/L	
	2024/11/18	TG	1.09	mmol/L	
	2024/11/18	HDL-C	0.81	mmol/L	L
	2024/11/18	LDL-C	3.68	mmol/L	H

Table S2. Blood lipid test results of 50 cases during influenza infection, as well as pre- and post-infection

Case Number	Reporting time	Test Data	Procedure	Test Results	Unit	Reference range	High/Low
1	2017/01/05	2016/11/18	T-CHO	5.1	mmol/L	<5.18	
	2017/01/05	2016/11/18	TG	1.72	mmol/L	<1.7	H
	2017/01/05	2016/11/18	HDL-C	1.07	mmol/L	>1.04	
	2017/01/05	2016/11/18	LDL-C	3.61	mmol/L	<3.64	
	2017/01/05	2017/01/03	T-CHO	5.17	mmol/L	<5.18	
	2017/01/05	2017/01/03	TG	1.74	mmol/L	<1.7	H
	2017/01/05	2017/01/03	HDL-C	1.14	mmol/L	>1.04	
	2017/01/05	2017/01/03	LDL-C	3.85	mmol/L	<3.64	H
	2017/01/05	2017/10/25	T-CHO	4.9	mmol/L	<5.18	
	2017/01/05	2017/10/25	TG	1.43	mmol/L	<1.7	
	2017/01/05	2017/10/25	HDL-C	1.05	mmol/L	>1.04	
	2017/01/05	2017/10/25	LDL-C	3.46	mmol/L	<3.64	
3	2017/02/17	2016/05/06	T-CHO	3.11	mmol/L	<5.18	
	2017/02/17	2016/05/06	TG	1.4	mmol/L	<1.7	
	2017/02/17	2016/05/06	HDL-C	0.72	mmol/L	>1.04	L
	2017/02/17	2016/05/06	LDL-C	1.96	mmol/L	<3.64	
	2017/02/17	2017/02/12	T-CHO	4.37	mmol/L	<5.18	
	2017/02/17	2017/02/12	TG	1	mmol/L	<1.7	
	2017/02/17	2017/02/12	HDL-C	0.92	mmol/L	>1.04	L
	2017/02/17	2017/02/12	LDL-C	2.65	mmol/L	<3.64	
	2017/02/17	2017/07/09	T-CHO	3.95	mmol/L	<5.18	
	2017/02/17	2017/07/09	TG	0.79	mmol/L	<1.7	
	2017/02/17	2017/07/09	HDL-C	0.77	mmol/L	>1.04	L
	2017/02/17	2017/07/09	LDL-C	2.81	mmol/L	<3.64	
4	2017/06/28	2017/03/03	T-CHO	4.83	mmol/L	<5.18	
	2017/06/28	2017/03/03	TG	1.02	mmol/L	<1.7	
	2017/06/28	2017/03/03	HDL-C	1.82	mmol/L	>1.04	
	2017/06/28	2017/03/03	LDL-C	2.57	mmol/L	<3.64	
	2017/06/28	2017/06/29	T-CHO	3.87	mmol/L	<5.18	
	2017/06/28	2017/06/29	TG	0.85	mmol/L	<1.7	
	2017/06/28	2017/06/29	HDL-C	1.34	mmol/L	>1.04	
	2017/06/28	2017/06/29	LDL-C	1.7	mmol/L	<3.64	
	2017/06/28	2018/01/31	T-CHO	4.16	mmol/L	<5.18	
	2017/06/28	2018/01/31	TG	0.92	mmol/L	<1.7	
	2017/06/28	2018/01/31	HDL-C	1.74	mmol/L	>1.04	
	2017/06/28	2018/01/31	LDL-C	1.99	mmol/L	<3.64	
11	2018/01/15	2018/01/04	T-CHO	2.51	mmol/L	<5.18	L
	2018/01/15	2018/01/04	TG	1.01	mmol/L	<1.7	
	2018/01/15	2018/01/04	HDL-C	0.61	mmol/L	>1.04	L
	2018/01/15	2018/01/04	LDL-C	1.55	mmol/L	<3.64	
	2018/01/15	2018/01/12	T-CHO	3.83	mmol/L	<5.18	
	2018/01/15	2018/01/12	TG	1.48	mmol/L	<1.7	
	2018/01/15	2018/01/12	HDL-C	0.68	mmol/L	>1.04	L
	2018/01/15	2018/01/12	LDL-C	2.51	mmol/L	<3.64	
	2018/01/15	2019/12/20	T-CHO	2.72	mmol/L	<5.18	
	2018/01/15	2019/12/20	TG	1.23	mmol/L	<1.7	
	2018/01/15	2019/12/20	HDL-C	0.68	mmol/L	>1.04	L
	2018/01/15	2019/12/20	LDL-C	1.58	mmol/L	<3.64	
14	2018/01/19	2018/01/06	T-CHO	4.38	mmol/L	<5.18	
	2018/01/19	2018/01/06	TG	1.05	mmol/L	<1.7	
	2018/01/19	2018/01/06	HDL-C	1.02	mmol/L	>1.04	L
	2018/01/19	2018/01/06	LDL-C	3.1	mmol/L	<3.64	
	2018/01/19	2018/01/16	T-CHO	3.91	mmol/L	<5.18	

	2018/01/19	2018/01/16	TG	1.52	mmol/L	<1.7	
	2018/01/19	2018/01/16	HDL-C	0.84	mmol/L	>1.04	L
	2018/01/19	2018/01/16	LDL-C	2.61	mmol/L	<3.64	
	2018/01/19	2019/12/11	T-CHO	3.39	mmol/L	<5.18	
	2018/01/19	2019/12/11	TG	3.92	mmol/L	<1.7	H
	2018/01/19	2019/12/11	HDL-C	0.39	mmol/L	>1.04	L
	2018/01/19	2019/12/11	LDL-C	1.86	mmol/L	<3.64	
17	2018/01/27	2017/09/21	T-CHO	4.94	mmol/L	<5.18	
	2018/01/27	2017/09/21	TG	1.53	mmol/L	<1.7	
	2018/01/27	2017/09/21	HDL-C	1.73	mmol/L	>1.04	
	2018/01/27	2017/09/21	LDL-C	2.61	mmol/L	<3.64	
	2018/01/27	2018/01/27	T-CHO	3.77	mmol/L	<5.18	
	2018/01/27	2018/01/27	TG	0.71	mmol/L	<1.7	
	2018/01/27	2018/01/27	HDL-C	1.05	mmol/L	>1.04	
	2018/01/27	2018/01/27	LDL-C	2.56	mmol/L	<3.64	
	2018/01/27	2018/03/12	T-CHO	3.94	mmol/L	<5.18	
	2018/01/27	2018/03/12	TG	1.24	mmol/L	<1.7	
	2018/01/27	2018/03/12	HDL-C	1.03	mmol/L	>1.04	L
	2018/01/27	2018/03/12	LDL-C	2.71	mmol/L	<3.64	
23	2018/02/14	2017/09/12	T-CHO	4.06	mmol/L	<5.18	
	2018/02/14	2017/09/12	TG	0.85	mmol/L	<1.7	
	2018/02/14	2017/09/12	HDL-C	0.98	mmol/L	>1.04	L
	2018/02/14	2017/09/12	LDL-C	2.64	mmol/L	<3.64	
	2018/02/14	2018/02/14	T-CHO	3.02	mmol/L	<5.18	
	2018/02/14	2018/02/14	TG	0.9	mmol/L	<1.7	
	2018/02/14	2018/02/14	HDL-C	0.92	mmol/L	>1.04	L
	2018/02/14	2018/02/14	LDL-C	1.65	mmol/L	<3.64	
	2018/02/14	2018/03/05	T-CHO	3.05	mmol/L	<5.18	
	2018/02/14	2018/03/05	TG	0.7	mmol/L	<1.7	
	2018/02/14	2018/03/05	HDL-C	0.76	mmol/L	>1.04	L
	2018/02/14	2018/03/05	LDL-C	1.93	mmol/L	<3.64	
26	2018/03/12	2017/12/22	T-CHO	5.68	mmol/L	<5.18	H
	2018/03/12	2017/12/22	TG	1.49	mmol/L	<1.7	
	2018/03/12	2017/12/22	HDL-C	1.84	mmol/L	>1.04	
	2018/03/12	2017/12/22	LDL-C	2.99	mmol/L	<3.64	
	2018/03/12	2018/03/07	T-CHO	7.37	mmol/L	<5.18	H
	2018/03/12	2018/03/07	TG	1	mmol/L	<1.7	
	2018/03/12	2018/03/07	HDL-C	3.2	mmol/L	>1.04	
	2018/03/12	2018/03/07	LDL-C	3.37	mmol/L	<3.64	
	2018/03/12	2023/12/19	T-CHO	2.8	mmol/L	<5.18	
	2018/03/12	2023/12/19	TG	0.48	mmol/L	<1.7	
	2018/03/12	2023/12/19	HDL-C	1.11	mmol/L	≥1.04	
	2018/03/12	2023/12/19	LDL-C	1.56	mmol/L	<3.64	
29	2018/03/20	2018/03/19	T-CHO	5.87	mmol/L	<5.18	H
	2018/03/20	2018/03/19	TG	1.96	mmol/L	<1.7	H
	2018/03/20	2018/03/19	HDL-C	1.1	mmol/L	>1.04	
	2018/03/20	2018/03/19	LDL-C	4.03	mmol/L	<3.64	H
	2018/03/20	2018/03/23	T-CHO	5.58	mmol/L	<5.18	H
	2018/03/20	2018/03/23	TG	1.82	mmol/L	<1.7	H
	2018/03/20	2018/03/23	HDL-C	1.05	mmol/L	>1.04	
	2018/03/20	2018/03/23	LDL-C	4.23	mmol/L	<3.64	H
	2018/03/20	2018/11/19	T-CHO	3.25	mmol/L	<5.18	
	2018/03/20	2018/11/19	TG	1.35	mmol/L	<1.7	
	2018/03/20	2018/11/19	HDL-C	1.04	mmol/L	>1.04	
	2018/03/20	2018/11/19	LDL-C	1.79	mmol/L	<3.64	
31	2018/11/26	2018/10/30	T-CHO	3.45	mmol/L	<5.18	

	2018/11/26	2018/10/30	TG	2.83	mmol/L	<1.7	H
	2018/11/26	2018/10/30	HDL-C	0.68	mmol/L	>1.04	L
	2018/11/26	2018/10/30	LDL-C	1.93	mmol/L	<3.64	
	2018/11/26	2018/11/23	T-CHO	3.81	mmol/L	<5.18	
	2018/11/26	2018/11/23	TG	3.03	mmol/L	<1.7	H
	2018/11/26	2018/11/23	HDL-C	0.65	mmol/L	>1.04	L
	2018/11/26	2018/11/23	LDL-C	2.06	mmol/L	<3.64	
	2018/11/26	2018/12/10	T-CHO	3.23	mmol/L	<5.18	
	2018/11/26	2018/12/10	TG	1.89	mmol/L	<1.7	H
	2018/11/26	2018/12/10	HDL-C	0.66	mmol/L	>1.04	L
	2018/11/26	2018/12/10	LDL-C	2.02	mmol/L	<3.64	
32	2018/12/20	2018/10/10	T-CHO	5.16	mmol/L	<5.18	
	2018/12/20	2018/10/10	TG	3.38	mmol/L	<1.7	H
	2018/12/20	2018/10/10	HDL-C	0.75	mmol/L	>1.04	L
	2018/12/20	2018/10/10	LDL-C	3.27	mmol/L	<3.64	
	2018/12/20	2018/12/16	T-CHO	8.9	mmol/L	<5.18	H
	2018/12/20	2018/12/16	TG	5.63	mmol/L	<1.7	H
	2018/12/20	2018/12/16	HDL-C	1.1	mmol/L	>1.04	
	2018/12/20	2018/12/16	LDL-C	5.39	mmol/L	<3.64	H
	2018/12/20	2018/12/29	T-CHO	3.47	mmol/L	<5.18	
	2018/12/20	2018/12/29	TG	2.11	mmol/L	<1.7	H
	2018/12/20	2018/12/29	HDL-C	0.59	mmol/L	>1.04	L
	2018/12/20	2018/12/29	LDL-C	2.14	mmol/L	<3.64	
33	2018/12/27	2017/02/15	T-CHO	5.04	mmol/L	<5.18	
	2018/12/27	2017/02/15	TG	1.23	mmol/L	<1.7	
	2018/12/27	2017/02/15	HDL-C	1.21	mmol/L	>1.04	
	2018/12/27	2017/02/15	LDL-C	2.9	mmol/L	<3.64	
	2018/12/27	2018/12/28	T-CHO	2.86	mmol/L	<5.18	
	2018/12/27	2018/12/28	TG	1.44	mmol/L	<1.7	
	2018/12/27	2018/12/28	HDL-C	0.77	mmol/L	>1.04	L
	2018/12/27	2018/12/28	LDL-C	1.47	mmol/L	<3.64	
	2018/12/27	2019/03/19	T-CHO	3.1	mmol/L	<5.18	
	2018/12/27	2019/03/19	TG	2.11	mmol/L	<1.7	H
	2018/12/27	2019/03/19	HDL-C	0.74	mmol/L	>1.04	L
	2018/12/27	2019/03/19	LDL-C	1.87	mmol/L	<3.64	
38	2019/01/09	2018/11/13	T-CHO	3.22	mmol/L	<5.18	
	2019/01/09	2018/11/13	TG	1.23	mmol/L	<1.7	
	2019/01/09	2018/11/13	HDL-C	0.76	mmol/L	>1.04	L
	2019/01/09	2018/11/13	LDL-C	1.98	mmol/L	<3.64	
	2019/01/09	2019/01/05	T-CHO	3.16	mmol/L	<5.18	
	2019/01/09	2019/01/05	TG	1.24	mmol/L	<1.7	
	2019/01/09	2019/01/05	HDL-C	0.81	mmol/L	>1.04	L
	2019/01/09	2019/01/05	LDL-C	1.88	mmol/L	<3.64	
	2019/01/09	2019/04/26	T-CHO	4.58	mmol/L	<5.18	
	2019/01/09	2019/04/26	TG	1.42	mmol/L	<1.7	
	2019/01/09	2019/04/26	HDL-C	1.1	mmol/L	>1.04	
	2019/01/09	2019/04/26	LDL-C	2.79	mmol/L	<3.64	
44	2019/01/15	2018/12/28	T-CHO	3.88	mmol/L	<5.18	
	2019/01/15	2018/12/28	TG	1.14	mmol/L	<1.7	
	2019/01/15	2018/12/28	HDL-C	1.12	mmol/L	>1.04	
	2019/01/15	2018/12/28	LDL-C	2.46	mmol/L	<3.64	
	2019/01/15	2019/01/11	T-CHO	3.37	mmol/L	<5.18	
	2019/01/15	2019/01/11	TG	1.3	mmol/L	<1.7	
	2019/01/15	2019/01/11	HDL-C	1.03	mmol/L	>1.04	L
	2019/01/15	2019/01/11	LDL-C	2.03	mmol/L	<3.64	
	2019/01/15	2022/09/17	T-CHO	3.84	mmol/L	<5.18	

	2019/01/15	2022/09/17	TG	0.9	mmol/L	<1.7	
	2019/01/15	2022/09/17	HDL-C	1.09	mmol/L	>1.04	
	2019/01/15	2022/09/17	LDL-C	2.44	mmol/L	<3.64	
46	2019/01/15	2016/10/20	T-CHO	3.49	mmol/L	<5.18	
	2019/01/15	2016/10/20	TG	1.03	mmol/L	<1.7	
	2019/01/15	2016/10/20	HDL-C	1.23	mmol/L	>1.04	
	2019/01/15	2016/10/20	LDL-C	2.07	mmol/L	<3.64	
	2019/01/15	2019/01/10	T-CHO	3.58	mmol/L	<5.18	
	2019/01/15	2019/01/10	TG	0.78	mmol/L	<1.7	
	2019/01/15	2019/01/10	HDL-C	1.14	mmol/L	>1.04	
	2019/01/15	2019/01/10	LDL-C	2.3	mmol/L	<3.64	
	2019/01/15	2019/02/14	T-CHO	3.66	mmol/L	<5.18	
	2019/01/15	2019/02/14	TG	1.03	mmol/L	<1.7	
	2019/01/15	2019/02/14	HDL-C	1.36	mmol/L	>1.04	
	2019/01/15	2019/02/14	LDL-C	2.05	mmol/L	<3.64	
47	2019/01/15	2018/02/07	T-CHO	3.59	mmol/L	<5.18	
	2019/01/15	2018/02/07	TG	1.28	mmol/L	<1.7	
	2019/01/15	2018/02/07	HDL-C	1.2	mmol/L	>1.04	
	2019/01/15	2018/02/07	LDL-C	2.11	mmol/L	<3.64	
	2019/01/15	2019/01/10	T-CHO	3.32	mmol/L	<5.18	
	2019/01/15	2019/01/10	TG	0.78	mmol/L	<1.7	
	2019/01/15	2019/01/10	HDL-C	1.22	mmol/L	>1.04	
	2019/01/15	2019/01/10	LDL-C	1.81	mmol/L	<3.64	
	2019/01/15	2019/04/13	T-CHO	3.19	mmol/L	<5.18	
	2019/01/15	2019/04/13	TG	0.83	mmol/L	<1.7	
	2019/01/15	2019/04/13	HDL-C	1.17	mmol/L	>1.04	
	2019/01/15	2019/04/13	LDL-C	1.62	mmol/L	<3.64	
48	2019/01/16	2017/02/20	T-CHO	3.55	mmol/L	<5.18	
	2019/01/16	2017/02/20	TG	0.41	mmol/L	<1.7	L
	2019/01/16	2017/02/20	HDL-C	1.7	mmol/L	>1.04	
	2019/01/16	2017/02/20	LDL-C	1.27	mmol/L	<3.64	
	2019/01/16	2019/01/15	T-CHO	3.9	mmol/L	<5.18	
	2019/01/16	2019/01/15	TG	0.87	mmol/L	<1.7	
	2019/01/16	2019/01/15	HDL-C	1.98	mmol/L	>1.04	
	2019/01/16	2019/01/15	LDL-C	1.21	mmol/L	<3.64	
	2019/01/16	2023/01/31	T-CHO	3.09	mmol/L	<5.18	
	2019/01/16	2023/01/31	TG	0.44	mmol/L	<1.7	
	2019/01/16	2023/01/31	HDL-C	1.27	mmol/L	>1.04	
	2019/01/16	2023/01/31	LDL-C	1.53	mmol/L	<3.64	
53	2019/01/18	2016/08/31	T-CHO	5.27	mmol/L	<5.18	H
	2019/01/18	2016/08/31	TG	1.46	mmol/L	<1.7	
	2019/01/18	2016/08/31	HDL-C	1.32	mmol/L	>1.04	
	2019/01/18	2016/08/31	LDL-C	3.37	mmol/L	<3.64	
	2019/01/18	2019/01/19	T-CHO	3.92	mmol/L	<5.18	
	2019/01/18	2019/01/19	TG	1.02	mmol/L	<1.7	
	2019/01/18	2019/01/19	HDL-C	1.02	mmol/L	>1.04	L
	2019/01/18	2019/01/19	LDL-C	2.57	mmol/L	<3.64	
	2019/01/18	2020/03/13	T-CHO	4.48	mmol/L	<5.18	
	2019/01/18	2020/03/13	TG	1.13	mmol/L	<1.7	
	2019/01/18	2020/03/13	HDL-C	1.35	mmol/L	>1.04	
	2019/01/18	2020/03/13	LDL-C	2.86	mmol/L	<3.64	
57	2019/01/24	2018/08/08	T-CHO	4.51	mmol/L	<5.18	
	2019/01/24	2018/08/08	TG	1.06	mmol/L	<1.7	
	2019/01/24	2018/08/08	HDL-C	1.4	mmol/L	>1.04	
	2019/01/24	2018/08/08	LDL-C	2.7	mmol/L	<3.64	
	2019/01/24	2019/01/21	T-CHO	5.82	mmol/L	<5.18	H

	2019/01/24	2019/01/21	TG	0.99	mmol/L	<1.7	
	2019/01/24	2019/01/21	HDL-C	1.65	mmol/L	>1.04	
	2019/01/24	2019/01/21	LDL-C	3.67	mmol/L	<3.64	H
	2019/01/24	2019/05/05	T-CHO	4.18	mmol/L	<5.18	
	2019/01/24	2019/05/05	TG	1.06	mmol/L	<1.7	
	2019/01/24	2019/05/05	HDL-C	1.51	mmol/L	>1.04	
	2019/01/24	2019/05/05	LDL-C	2.08	mmol/L	<3.64	
59	2019/01/24	2018/06/12	T-CHO	4.93	mmol/L	<5.18	
	2019/01/24	2018/06/12	TG	1.14	mmol/L	<1.7	
	2019/01/24	2018/06/12	HDL-C	1.68	mmol/L	>1.04	
	2019/01/24	2018/06/12	LDL-C	2.76	mmol/L	<3.64	
	2019/01/24	2019/01/24	T-CHO	4.95	mmol/L	<5.18	
	2019/01/24	2019/01/24	TG	0.53	mmol/L	<1.7	
	2019/01/24	2019/01/24	HDL-C	2.11	mmol/L	>1.04	
	2019/01/24	2019/01/24	LDL-C	2.29	mmol/L	<3.64	
	2019/01/24	2019/01/25	T-CHO	4.63	mmol/L	<5.18	
	2019/01/24	2019/01/25	TG	0.56	mmol/L	<1.7	
	2019/01/24	2019/01/25	HDL-C	1.83	mmol/L	>1.04	
	2019/01/24	2019/01/25	LDL-C	2.25	mmol/L	<3.64	
60	2019/01/28	2017/11/03	T-CHO	3.72	mmol/L	<5.18	
	2019/01/28	2017/11/03	TG	5.86	mmol/L	<1.7	H
	2019/01/28	2017/11/03	HDL-C	0.94	mmol/L	>1.04	L
	2019/01/28	2017/11/03	LDL-C	1.44	mmol/L	<3.64	
	2019/01/28	2019/01/25	T-CHO	4.32	mmol/L	<5.18	
	2019/01/28	2019/01/25	TG	2.49	mmol/L	<1.7	H
	2019/01/28	2019/01/25	HDL-C	0.97	mmol/L	>1.04	L
	2019/01/28	2019/01/25	LDL-C	2.54	mmol/L	<3.64	
	2019/01/28	2019/07/13	T-CHO	4.76	mmol/L	<5.18	
	2019/01/28	2019/07/13	TG	2.43	mmol/L	<1.7	H
	2019/01/28	2019/07/13	HDL-C	1.09	mmol/L	>1.04	
	2019/01/28	2019/07/13	LDL-C	2.88	mmol/L	<3.64	
61	2019/01/28	2017/09/27	T-CHO	3.59	mmol/L	<5.18	
	2019/01/28	2017/09/27	TG	0.9	mmol/L	<1.7	
	2019/01/28	2017/09/27	HDL-C	1.93	mmol/L	>1.04	
	2019/01/28	2017/09/27	LDL-C	1.05	mmol/L	<3.64	
	2019/01/28	2019/01/25	T-CHO	2.86	mmol/L	<5.18	
	2019/01/28	2019/01/25	TG	0.57	mmol/L	<1.7	
	2019/01/28	2019/01/25	HDL-C	1.48	mmol/L	>1.04	
	2019/01/28	2019/01/25	LDL-C	1	mmol/L	<3.64	
	2019/01/28	2019/11/27	T-CHO	3.26	mmol/L	<5.18	
	2019/01/28	2019/11/27	TG	0.75	mmol/L	<1.7	
	2019/01/28	2019/11/27	HDL-C	1.62	mmol/L	>1.04	
	2019/01/28	2019/11/27	LDL-C	1.12	mmol/L	<3.64	
64	2019/02/19	2017/07/29	T-CHO	3.04	mmol/L	<5.18	
	2019/02/19	2017/07/29	TG	0.83	mmol/L	<1.7	
	2019/02/19	2017/07/29	HDL-C	1.3	mmol/L	>1.04	
	2019/02/19	2017/07/29	LDL-C	1.37	mmol/L	<3.64	
	2019/02/19	2019/02/19	T-CHO	3	mmol/L	<5.18	
	2019/02/19	2019/02/19	TG	0.96	mmol/L	<1.7	
	2019/02/19	2019/02/19	HDL-C	1.1	mmol/L	>1.04	
	2019/02/19	2019/02/19	LDL-C	1.57	mmol/L	<3.64	
	2019/02/19	2019/04/09	T-CHO	2.6	mmol/L	<5.18	
	2019/02/19	2019/04/09	TG	1.25	mmol/L	<1.7	
	2019/02/19	2019/04/09	HDL-C	0.83	mmol/L	>1.04	L
	2019/02/19	2019/04/09	LDL-C	1.25	mmol/L	<3.64	
72	2019/04/30	2019/04/25	T-CHO	2.32	mmol/L	<5.18	

	2019/04/30	2019/04/25	TG	1.24	mmol/L	<1.7	
	2019/04/30	2019/04/25	HDL-C	0.47	mmol/L	>1.04	L
	2019/04/30	2019/04/25	LDL-C	1.33	mmol/L	<3.64	
	2019/04/30	2019/04/30	T-CHO	3.41	mmol/L	<5.18	
	2019/04/30	2019/04/30	TG	1.51	mmol/L	<1.7	
	2019/04/30	2019/04/30	HDL-C	0.61	mmol/L	>1.04	L
	2019/04/30	2019/04/30	LDL-C	2.28	mmol/L	<3.64	
	2019/04/30	2019/05/09	TG	0.97	mmol/L	<1.7	
	2019/04/30	2019/06/25	T-CHO	4.23	mmol/L	<5.18	
	2019/04/30	2019/06/25	TG	2.58	mmol/L	<1.7	H
	2019/04/30	2019/06/25	HDL-C	1.36	mmol/L	>1.04	
73	2019/05/08	2016/09/09	T-CHO	3.04	mmol/L	<5.18	
	2019/05/08	2016/09/09	TG	1.18	mmol/L	<1.7	
	2019/05/08	2016/09/09	HDL-C	0.97	mmol/L	>1.04	L
	2019/05/08	2016/09/09	LDL-C	1.54	mmol/L	<3.64	
	2019/05/08	2019/05/03	T-CHO	3.76	mmol/L	<5.18	
	2019/05/08	2019/05/03	TG	1.48	mmol/L	<1.7	
	2019/05/08	2019/05/03	HDL-C	0.74	mmol/L	>1.04	L
	2019/05/08	2019/05/03	LDL-C	2.7	mmol/L	<3.64	
	2019/05/08	2019/11/05	T-CHO	2.74	mmol/L	<5.18	
	2019/05/08	2019/11/05	TG	0.84	mmol/L	<1.7	
	2019/05/08	2019/11/05	HDL-C	0.78	mmol/L	>1.04	L
	2019/05/08	2019/11/05	LDL-C	1.51	mmol/L	<3.64	
77	2019/11/26	2019/10/24	T-CHO	3.21	mmol/L	<5.18	
	2019/11/26	2019/10/24	TG	0.43	mmol/L	<1.7	
	2019/11/26	2019/10/24	HDL-C	1.18	mmol/L	>1.04	
	2019/11/26	2019/10/24	LDL-C	1.72	mmol/L	<3.64	
	2019/11/26	2019/11/26	T-CHO	2.87	mmol/L	<5.18	
	2019/11/26	2019/11/26	TG	0.42	mmol/L	<1.7	
	2019/11/26	2019/11/26	HDL-C	0.94	mmol/L	>1.04	L
	2019/11/26	2019/11/26	LDL-C	1.69	mmol/L	<3.64	
	2019/11/26	2020/06/21	T-CHO	5.2	mmol/L	<5.18	H
	2019/11/26	2020/06/21	TG	0.7	mmol/L	<1.7	
	2019/11/26	2020/06/21	HDL-C	1.01	mmol/L	>1.04	L
	2019/11/26	2020/06/21	LDL-C	3.71	mmol/L	<3.64	H
81	2019/12/26	2019/12/24	T-CHO	3.73	mmol/L	<5.18	
	2019/12/26	2019/12/24	TG	1.26	mmol/L	<1.7	
	2019/12/26	2019/12/24	HDL-C	0.92	mmol/L	>1.04	L
	2019/12/26	2019/12/24	LDL-C	2.32	mmol/L	<3.64	
	2019/12/26	2019/12/27	T-CHO	3.36	mmol/L	<5.18	
	2019/12/26	2019/12/27	TG	1.29	mmol/L	<1.7	
	2019/12/26	2019/12/27	HDL-C	0.85	mmol/L	>1.04	L
	2019/12/26	2019/12/27	LDL-C	2.06	mmol/L	<3.64	
	2019/12/26	2021/07/16	T-CHO	4.82	mmol/L	<5.18	
	2019/12/26	2021/07/16	TG	0.94	mmol/L	<1.7	
	2019/12/26	2021/07/16	HDL-C	1.71	mmol/L	>1.04	
	2019/12/26	2021/07/16	LDL-C	2.61	mmol/L	<3.64	
84	2019/12/30	2019/12/06	T-CHO	4	mmol/L	<5.18	
	2019/12/30	2019/12/06	TG	1.47	mmol/L	<1.7	
	2019/12/30	2019/12/06	HDL-C	1.04	mmol/L	>1.04	
	2019/12/30	2019/12/06	LDL-C	2.31	mmol/L	<3.64	
	2019/12/30	2019/12/26	T-CHO	3.24	mmol/L	<5.18	
	2019/12/30	2019/12/26	TG	1.19	mmol/L	<1.7	
	2019/12/30	2019/12/26	HDL-C	0.97	mmol/L	>1.04	L
	2019/12/30	2019/12/26	LDL-C	1.84	mmol/L	<3.64	
	2019/12/30	2021/08/31	T-CHO	3.73	mmol/L	<5.18	

	2019/12/30	2021/08/31	TG	1.81	mmol/L	<1.7	H
	2019/12/30	2021/08/31	HDL-C	1.1	mmol/L	>1.04	
	2019/12/30	2021/08/31	LDL-C	1.85	mmol/L	<3.64	
86	2020/01/02	2019/10/11	T-CHO	4.47	mmol/L	<5.18	
	2020/01/02	2019/10/11	TG	0.81	mmol/L	<1.7	
	2020/01/02	2019/10/11	HDL-C	1.36	mmol/L	>1.04	
	2020/01/02	2019/10/11	LDL-C	2.64	mmol/L	<3.64	
	2020/01/02	2019/12/31	T-CHO	4.79	mmol/L	<5.18	
	2020/01/02	2019/12/31	TG	0.7	mmol/L	<1.7	
	2020/01/02	2019/12/31	HDL-C	1.31	mmol/L	>1.04	
	2020/01/02	2019/12/31	LDL-C	3.02	mmol/L	<3.64	
	2020/01/02	2020/02/05	T-CHO	3.58	mmol/L	<5.18	
	2020/01/02	2020/02/05	TG	0.71	mmol/L	<1.7	
	2020/01/02	2020/02/05	HDL-C	1.38	mmol/L	>1.04	
	2020/01/02	2020/02/05	LDL-C	1.81	mmol/L	<3.64	
87	2020/01/03	2019/11/30	T-CHO	3.67	mmol/L	<5.18	
	2020/01/03	2019/11/30	TG	0.79	mmol/L	<1.7	
	2020/01/03	2019/11/30	HDL-C	1.12	mmol/L	>1.04	
	2020/01/03	2019/11/30	LDL-C	2.41	mmol/L	<3.64	
	2020/01/03	2020/01/04	T-CHO	2.93	mmol/L	<5.18	
	2020/01/03	2020/01/04	TG	0.6	mmol/L	<1.7	
	2020/01/03	2020/01/04	HDL-C	1.3	mmol/L	>1.04	
	2020/01/03	2020/01/04	LDL-C	1.52	mmol/L	<3.64	
	2020/01/03	2023/08/24	T-CHO	4.2	mmol/L	<5.18	
	2020/01/03	2023/08/24	TG	0.9	mmol/L	<1.7	
	2020/01/03	2023/08/24	HDL-C	1.2	mmol/L	≥1.04	
	2020/01/03	2023/08/24	LDL-C	2.59	mmol/L	<3.64	
89	2020/01/06	2018/12/18	T-CHO	3.82	mmol/L	<5.18	
	2020/01/06	2018/12/18	TG	0.9	mmol/L	<1.7	
	2020/01/06	2018/12/18	HDL-C	1.61	mmol/L	>1.04	
	2020/01/06	2018/12/18	LDL-C	1.82	mmol/L	<3.64	
	2020/01/06	2019/12/30	T-CHO	3.07	mmol/L	<5.18	
	2020/01/06	2019/12/30	TG	1.04	mmol/L	<1.7	
	2020/01/06	2019/12/30	HDL-C	1.24	mmol/L	>1.04	
	2020/01/06	2019/12/30	LDL-C	1.4	mmol/L	<3.64	
	2020/01/06	2021/08/03	T-CHO	2.92	mmol/L	<5.18	
	2020/01/06	2021/08/03	TG	0.85	mmol/L	<1.7	
	2020/01/06	2021/08/03	HDL-C	1.05	mmol/L	>1.04	
	2020/01/06	2021/08/03	LDL-C	1.49	mmol/L	<3.64	
92	2020/01/07	2019/09/07	T-CHO	2.67	mmol/L	<5.18	
	2020/01/07	2019/09/07	TG	1.14	mmol/L	<1.7	
	2020/01/07	2019/09/07	HDL-C	0.54	mmol/L	>1.04	L
	2020/01/07	2019/09/07	LDL-C	1.78	mmol/L	<3.64	
	2020/01/07	2020/01/07	T-CHO	1.24	mmol/L	<5.18	
	2020/01/07	2020/01/07	TG	0.46	mmol/L	<1.7	
	2020/01/07	2020/01/07	HDL-C	0.43	mmol/L	>1.04	L
	2020/01/07	2020/01/07	LDL-C	0.75	mmol/L	<3.64	
	2020/01/07	2020/06/10	T-CHO	2.84	mmol/L	<5.18	
	2020/01/07	2020/06/10	TG	1	mmol/L	<1.7	
	2020/01/07	2020/06/10	HDL-C	0.9	mmol/L	>1.04	L
	2020/01/07	2020/06/10	LDL-C	1.64	mmol/L	<3.64	
95	2020/01/14	2018/02/07	T-CHO	2.8	mmol/L	<5.18	L
	2020/01/14	2018/02/07	TG	1.02	mmol/L	<1.7	
	2020/01/14	2018/02/07	HDL-C	0.92	mmol/L	>1.04	L
	2020/01/14	2018/02/07	LDL-C	1.51	mmol/L	<3.64	
	2020/01/14	2020/01/09	T-CHO	2.42	mmol/L	<5.18	

	2020/01/14	2020/01/09	TG	1.29	mmol/L	<1.7	
	2020/01/14	2020/01/09	HDL-C	0.82	mmol/L	>1.04	L
	2020/01/14	2020/01/09	LDL-C	1.38	mmol/L	<3.64	
	2020/01/14	2020/03/23	T-CHO	2.61	mmol/L	<5.18	
	2020/01/14	2020/03/23	TG	0.99	mmol/L	<1.7	
	2020/01/14	2020/03/23	HDL-C	0.94	mmol/L	>1.04	L
	2020/01/14	2020/03/23	LDL-C	1.42	mmol/L	<3.64	
97	2020/01/15	2019/11/26	T-CHO	8.47	mmol/L	<5.18	H
	2020/01/15	2019/11/26	TG	3.78	mmol/L	<1.7	H
	2020/01/15	2019/11/26	HDL-C	1.61	mmol/L	>1.04	
	2020/01/15	2019/11/26	LDL-C	4.98	mmol/L	<3.64	H
	2020/01/15	2020/01/14	T-CHO	7.86	mmol/L	<5.18	H
	2020/01/15	2020/01/14	TG	3.87	mmol/L	<1.7	H
	2020/01/15	2020/01/14	HDL-C	1.5	mmol/L	>1.04	
	2020/01/15	2020/01/14	LDL-C	4.96	mmol/L	<3.64	H
	2020/01/15	2021/04/22	T-CHO	5.13	mmol/L	<5.18	
	2020/01/15	2021/04/22	TG	1.34	mmol/L	<1.7	
	2020/01/15	2021/04/22	HDL-C	1.04	mmol/L	>1.04	
	2020/01/15	2021/04/22	LDL-C	3.63	mmol/L	<3.64	
98	2020/01/16	2019/12/09	T-CHO	4.15	mmol/L	<5.18	
	2020/01/16	2019/12/09	TG	0.78	mmol/L	<1.7	
	2020/01/16	2019/12/09	HDL-C	1.41	mmol/L	>1.04	
	2020/01/16	2019/12/09	LDL-C	2.28	mmol/L	<3.64	
	2020/01/16	2020/01/15	T-CHO	3.14	mmol/L	<5.18	
	2020/01/16	2020/01/15	TG	1.02	mmol/L	<1.7	
	2020/01/16	2020/01/15	HDL-C	0.97	mmol/L	>1.04	L
	2020/01/16	2020/01/15	LDL-C	1.83	mmol/L	<3.64	
	2020/01/16	2022/04/02	T-CHO	4.39	mmol/L	<5.18	
	2020/01/16	2022/04/02	TG	0.72	mmol/L	<1.7	
	2020/01/16	2022/04/02	HDL-C	1.58	mmol/L	>1.04	
	2020/01/16	2022/04/02	LDL-C	2.13	mmol/L	<3.64	
116	2022/08/26	2021/11/12	T-CHO	4.44	mmol/L	<5.18	
	2022/08/26	2021/11/12	TG	1.02	mmol/L	<1.7	
	2022/08/26	2021/11/12	HDL-C	1.07	mmol/L	>1.04	
	2022/08/26	2021/11/12	LDL-C	2.72	mmol/L	<3.64	
	2022/08/26	2022/08/25	T-CHO	2.13	mmol/L	<5.18	
	2022/08/26	2022/08/25	TG	1.2	mmol/L	<1.7	
	2022/08/26	2022/08/25	HDL-C	0.69	mmol/L	>1.04	L
	2022/08/26	2022/08/25	LDL-C	1.05	mmol/L	<3.64	
	2022/08/26	2025/01/04	T-CHO	3.55	mmol/L	<5.18	
	2022/08/26	2025/01/04	TG	1.32	mmol/L	<1.7	
	2022/08/26	2025/01/04	HDL-C	1.06	mmol/L	≥1.04	
	2022/08/26	2025/01/04	LDL-C	1.94	mmol/L	0-3.4	
118	2022/07/06	2021/06/05	T-CHO	4.44	mmol/L	<5.18	
	2022/07/06	2021/06/05	TG	0.94	mmol/L	<1.7	
	2022/07/06	2021/06/05	HDL-C	1.22	mmol/L	>1.04	
	2022/07/06	2021/06/05	LDL-C	2.73	mmol/L	<3.64	
	2022/07/06	2022/07/04	T-CHO	3.73	mmol/L	<5.18	
	2022/07/06	2022/07/04	TG	0.65	mmol/L	<1.7	
	2022/07/06	2022/07/04	HDL-C	1.11	mmol/L	>1.04	
	2022/07/06	2022/07/04	LDL-C	2.22	mmol/L	<3.64	
	2022/07/06	2021/08/28	T-CHO	4.68	mmol/L	<5.18	
	2022/07/06	2021/08/28	TG	0.87	mmol/L	<1.7	
	2022/07/06	2021/08/28	HDL-C	1.17	mmol/L	>1.04	
	2022/07/06	2021/08/28	LDL-C	2.84	mmol/L	<3.64	
120	2022/02/15	2022/01/19	T-CHO	5.44	mmol/L	<5.18	H

	2022/02/15	2022/01/19	TG	1.8	mmol/L	<1.7	H
	2022/02/15	2022/01/19	HDL-C	1.81	mmol/L	>1.04	
	2022/02/15	2022/01/19	LDL-C	2.31	mmol/L	<3.64	
	2022/02/15	2022/02/09	T-CHO	4.11	mmol/L	<5.18	
	2022/02/15	2022/02/09	TG	1.29	mmol/L	<1.7	
	2022/02/15	2022/02/09	HDL-C	1.16	mmol/L	>1.04	
	2022/02/15	2022/02/09	LDL-C	2.01	mmol/L	<3.64	
	2022/02/15	2022/04/04	T-CHO	5.53	mmol/L	<5.18	H
	2022/02/15	2022/04/04	TG	2.37	mmol/L	<1.7	H
	2022/02/15	2022/04/04	HDL-C	1.08	mmol/L	>1.04	
	2022/02/15	2022/04/04	LDL-C	3.15	mmol/L	<3.64	
126	2022/01/12	2018/12/26	T-CHO	4.15	mmol/L	<5.18	
	2022/01/12	2018/12/26	TG	1.06	mmol/L	<1.7	
	2022/01/12	2018/12/26	HDL-C	1.32	mmol/L	>1.04	
	2022/01/12	2018/12/26	LDL-C	2.22	mmol/L	<3.64	
	2022/01/12	2022/01/06	T-CHO	3.99	mmol/L	<5.18	
	2022/01/12	2022/01/06	TG	0.41	mmol/L	<1.7	
	2022/01/12	2022/01/06	HDL-C	1.6	mmol/L	>1.04	
	2022/01/12	2022/01/06	LDL-C	1.83	mmol/L	<3.64	
	2022/01/12	2023/01/07	T-CHO	3.12	mmol/L	<5.18	
	2022/01/12	2023/01/07	TG	1.15	mmol/L	<1.7	
	2022/01/12	2023/01/07	HDL-C	0.9	mmol/L	>1.04	L
	2022/01/12	2023/01/07	LDL-C	1.87	mmol/L	<3.64	
130	2023/06/10	2023/06/02	T-CHO	3.58	mmol/L	<5.18	
	2023/06/10	2023/06/02	TG	1.76	mmol/L	<1.7	H
	2023/06/10	2023/06/02	HDL-C	0.68	mmol/L	≥1.04	L
	2023/06/10	2023/06/02	LDL-C	2.31	mmol/L	<3.64	
	2023/06/10	2023/06/09	T-CHO	2.35	mmol/L	<5.18	
	2023/06/10	2023/06/09	TG	1	mmol/L	<1.7	
	2023/06/10	2023/06/09	HDL-C	0.71	mmol/L	≥1.04	L
	2023/06/10	2023/06/09	LDL-C	1.4	mmol/L	<3.64	
	2023/06/10	2024/06/14	T-CHO	3.31	mmol/L	<5.18	
	2023/06/10	2024/06/14	TG	1.13	mmol/L	<1.7	
	2023/06/10	2024/06/14	HDL-C	0.83	mmol/L	≥1.04	L
	2023/06/10	2024/06/14	LDL-C	1.87	mmol/L	<3.64	
133	2023/02/23	2021/11/08	T-CHO	4.92	mmol/L	<5.18	
	2023/02/23	2021/11/08	TG	1.18	mmol/L	<1.7	
	2023/02/23	2021/11/08	HDL-C	1.03	mmol/L	>1.04	L
	2023/02/23	2021/11/08	LDL-C	2.92	mmol/L	<3.64	
	2023/02/23	2023/02/19	T-CHO	5.19	mmol/L	<5.18	H
	2023/02/23	2023/02/19	TG	1.4	mmol/L	<1.7	
	2023/02/23	2023/02/19	HDL-C	1.36	mmol/L	≥1.04	
	2023/02/23	2023/02/19	LDL-C	3.02	mmol/L	<3.64	
	2023/02/23	2023/12/12	T-CHO	7.35	mmol/L	<5.18	H
	2023/02/23	2023/12/12	TG	2.18	mmol/L	<1.7	H
	2023/02/23	2023/12/12	HDL-C	1.74	mmol/L	≥1.04	
	2023/02/23	2023/12/12	LDL-C	4.29	mmol/L	<3.64	H
135	2023/02/16	2023/02/09	T-CHO	3.51	mmol/L	<5.18	
	2023/02/16	2023/02/09	TG	1.58	mmol/L	<1.7	
	2023/02/16	2023/02/09	HDL-C	0.69	mmol/L	>1.04	L
	2023/02/16	2023/02/09	LDL-C	2	mmol/L	<3.64	
	2023/02/16	2023/02/10	T-CHO	3.4	mmol/L	<5.18	
	2023/02/16	2023/02/10	TG	1.86	mmol/L	<1.7	H
	2023/02/16	2023/02/10	HDL-C	0.63	mmol/L	>1.04	L
	2023/02/16	2023/02/10	LDL-C	2.11	mmol/L	<3.64	
	2023/02/16	2023/09/25	T-CHO	4.78	mmol/L	<5.18	

	2023/02/16	2023/09/25	TG	4.46	mmol/L	<1.7	H
	2023/02/16	2023/09/25	HDL-C	0.84	mmol/L	≥1.04	L
	2023/02/16	2023/09/25	LDL-C	2.5	mmol/L	<3.64	
136	2023/03/24	2023/03/09	T-CHO	3.05	mmol/L	<5.18	
	2023/03/24	2023/03/09	TG	0.82	mmol/L	<1.7	
	2023/03/24	2023/03/09	HDL-C	0.42	mmol/L	≥1.04	L
	2023/03/24	2023/03/09	LDL-C	1.61	mmol/L	<3.64	
	2023/03/24	2023/03/24	T-CHO	2.3	mmol/L	<5.18	
	2023/03/24	2023/03/24	TG	0.83	mmol/L	<1.7	
	2023/03/24	2023/03/24	HDL-C	0.56	mmol/L	≥1.04	L
	2023/03/24	2023/03/24	LDL-C	1.53	mmol/L	<3.64	
	2023/03/24	2023/03/27	T-CHO	1.71	mmol/L	<5.18	
	2023/03/24	2023/03/27	TG	0.44	mmol/L	<1.7	
	2023/03/24	2023/03/27	HDL-C	0.58	mmol/L	≥1.04	L
	2023/03/24	2023/03/27	LDL-C	0.95	mmol/L	<3.64	
142	2023/06/03	2023/05/31	T-CHO	2.58	mmol/L	<5.18	
	2023/06/03	2023/05/31	TG	0.52	mmol/L	<1.7	
	2023/06/03	2023/05/31	HDL-C	0.6	mmol/L	≥1.04	L
	2023/06/03	2023/05/31	LDL-C	1.75	mmol/L	<3.64	
	2023/06/03	2023/06/06	T-CHO	1.92	mmol/L	<5.18	
	2023/06/03	2023/06/06	TG	0.82	mmol/L	<1.7	
	2023/06/03	2023/06/06	HDL-C	0.39	mmol/L	≥1.04	L
	2023/06/03	2023/06/06	LDL-C	1.29	mmol/L	<3.64	
	2023/06/03	2023/10/25	T-CHO	3.2	mmol/L	<5.18	
	2023/06/03	2023/10/25	TG	0.69	mmol/L	<1.7	
	2023/06/03	2023/10/25	HDL-C	0.91	mmol/L	≥1.04	L
	2023/06/03	2023/10/25	LDL-C	1.89	mmol/L	<3.64	
145	2023/06/15	2023/05/09	T-CHO	2.75	mmol/L	<5.18	
	2023/06/15	2023/05/09	TG	0.87	mmol/L	<1.7	
	2023/06/15	2023/05/09	HDL-C	1.21	mmol/L	≥1.04	
	2023/06/15	2023/05/09	LDL-C	1.2	mmol/L	<3.64	
	2023/06/15	2023/06/08	T-CHO	3.14	mmol/L	<5.18	
	2023/06/15	2023/06/08	TG	1.31	mmol/L	<1.7	
	2023/06/15	2023/06/08	HDL-C	1.02	mmol/L	≥1.04	L
	2023/06/15	2023/06/08	LDL-C	1.33	mmol/L	<3.64	
	2023/06/15	2023/06/27	T-CHO	3.21	mmol/L	<5.18	
	2023/06/15	2023/06/27	TG	1.23	mmol/L	<1.7	
	2023/06/15	2023/06/27	HDL-C	1.09	mmol/L	≥1.04	
	2023/06/15	2023/06/27	LDL-C	1.35	mmol/L	<3.64	
147	2023/04/11	2023/01/28	T-CHO	4.21	mmol/L	<5.18	
	2023/04/11	2023/01/28	TG	1.47	mmol/L	<1.7	
	2023/04/11	2023/01/28	HDL-C	0.65	mmol/L	>1.04	L
	2023/04/11	2023/01/28	LDL-C	2.87	mmol/L	<3.64	
	2023/04/11	2023/04/11	T-CHO	3.12	mmol/L	<5.18	
	2023/04/11	2023/04/11	TG	1.8	mmol/L	<1.7	H
	2023/04/11	2023/04/11	HDL-C	0.58	mmol/L	≥1.04	L
	2023/04/11	2023/04/11	LDL-C	1.84	mmol/L	<3.64	
	2023/04/11	2023/05/10	T-CHO	3.16	mmol/L	<5.18	
	2023/04/11	2023/05/10	TG	1.29	mmol/L	<1.7	
	2023/04/11	2023/05/10	HDL-C	0.53	mmol/L	≥1.04	L
	2023/04/11	2023/05/10	LDL-C	2.12	mmol/L	<3.64	
159	2024/03/18	2024/02/13	T-CHO	4.04	mmol/L	<5.18	
	2024/03/18	2024/02/13	TG	1.66	mmol/L	<1.7	
	2024/03/18	2024/02/13	HDL-C	1.17	mmol/L	≥1.04	
	2024/03/18	2024/02/13	LDL-C	2.25	mmol/L	<3.64	
	2024/03/18	2024/03/17	T-CHO	3.51	mmol/L	<5.18	

	2024/03/18	2024/03/17	TG	2.16	mmol/L	<1.7	H
	2024/03/18	2024/03/17	HDL-C	1.02	mmol/L	≥1.04	L
	2024/03/18	2024/03/17	LDL-C	1.74	mmol/L	<3.64	
	2024/03/18	2024/05/22	T-CHO	4.66	mmol/L	<5.18	
	2024/03/18	2024/05/22	TG	3.02	mmol/L	<1.7	H
	2024/03/18	2024/05/22	HDL-C	1	mmol/L	≥1.04	L
	2024/03/18	2024/05/22	LDL-C	2.53	mmol/L	<3.64	
160	2024/02/23	2024/01/22	T-CHO	4.65	mmol/L	<5.18	
	2024/02/23	2024/01/22	TG	3.89	mmol/L	<1.7	H
	2024/02/23	2024/01/22	HDL-C	0.7	mmol/L	≥1.04	L
	2024/02/23	2024/01/22	LDL-C	2.26	mmol/L	<3.64	
	2024/02/23	2024/02/19	T-CHO	4.11	mmol/L	<5.18	
	2024/02/23	2024/02/19	TG	2.86	mmol/L	<1.7	H
	2024/02/23	2024/02/19	HDL-C	0.69	mmol/L	≥1.04	L
	2024/02/23	2024/02/19	LDL-C	2.13	mmol/L	<3.64	
	2024/02/23	2024/03/21	T-CHO	4.46	mmol/L	<5.18	
	2024/02/23	2024/03/21	TG	2.75	mmol/L	<1.7	H
	2024/02/23	2024/03/21	HDL-C	0.84	mmol/L	≥1.04	L
	2024/02/23	2024/03/21	LDL-C	2.5	mmol/L	<3.64	
161	2024/04/16	2022/08/11	T-CHO	5.23	mmol/L	<5.18	H
	2024/04/16	2022/08/11	TG	1.56	mmol/L	<1.7	
	2024/04/16	2022/08/11	HDL-C	0.89	mmol/L	>1.04	L
	2024/04/16	2022/08/11	LDL-C	3.48	mmol/L	<3.64	
	2024/04/16	2024/04/09	T-CHO	7.14	mmol/L	<5.18	H
	2024/04/16	2024/04/09	TG	4.97	mmol/L	<1.7	H
	2024/04/16	2024/04/09	HDL-C	1.17	mmol/L	≥1.04	
	2024/04/16	2024/04/09	LDL-C	4.14	mmol/L	<3.64	H
	2024/04/16	2024/04/30	T-CHO	3.79	mmol/L	<5.18	
	2024/04/16	2024/04/30	TG	1.05	mmol/L	<1.7	
	2024/04/16	2024/04/30	HDL-C	1.25	mmol/L	≥1.04	
	2024/04/16	2024/04/30	LDL-C	1.97	mmol/L	<3.64	
162	2024/06/20	2016/07/04	T-CHO	2.9	mmol/L	<5.18	
	2024/06/20	2016/07/04	TG	1.76	mmol/L	<1.7	H
	2024/06/20	2016/07/04	HDL-C	1.16	mmol/L	>1.04	
	2024/06/20	2016/07/04	LDL-C	1.42	mmol/L	<3.64	
	2024/06/20	2024/06/19	T-CHO	4.43	mmol/L	<5.18	
	2024/06/20	2024/06/19	TG	1.89	mmol/L	<1.7	H
	2024/06/20	2024/06/19	HDL-C	0.91	mmol/L	≥1.04	L
	2024/06/20	2024/06/19	LDL-C	3.19	mmol/L	<3.64	
	2024/06/20	2024/06/26	T-CHO	3.99	mmol/L	<5.18	
	2024/06/20	2024/06/26	TG	1.52	mmol/L	<1.7	
	2024/06/20	2024/06/26	HDL-C	0.9	mmol/L	≥1.04	L
	2024/06/20	2024/06/26	LDL-C	2.64	mmol/L	<3.64	

Table S3. Primers of housekeeping gene and other targeted genes

Target gene	Primer pairs (5'→3')
Homo-GAPDH	F: 5’ - GGGTGTGAACCATGAGAAGTATGA- 3’<br>R: 5’ - GGTGCAGGAGGCATTGCT- 3’
Homo-HMGCR	F: 5’ - GCAGGACCCCTTTGCTTAGA- 3’<br>R: 5’ - GCACCTCCACCAAGACCTAT-3’
Homo-HMGCS	F: 5’ - CCTGCCAAGAAAGTACCAAGA- 3’<br>R: 5’ - GTCTTGCACCTCACAGAGTATC-3’
Homo-TNF-α	F: 5’ - CCAACTGTCACTCATTGCTGA - 3’<br>R: 5’ - TTCCAAGAAGGAGACCATGTTT -3’
Homo-IL-6	F: 5’ - AGACAGCCACTCACCTCTTCAG - 3’<br>R: 5’ - TTCTGCCAGTGCCTCTTTGCTG -3’
Mouse-β-actin	F: 5’ - GAGACCTTCAACACCCCAGC - 3’<br>R: 5’ - ATGTCACGCACGATTTCCC - 3’
Mouse-HMGCR	F: 5’ - AGCTTGCCCGAATTGTATGTG - 3’<br>R: 5’ - TCTGTTGTGAACCATGTGACTTC - 3’
Mouse-HMGCS	F: 5’ - TGTGGTTCAGAAACTGATGG - 3’<br>R: 5’ - TGTCTCCTGCAACTACCAGA -3’
Mouse-IL-6	F: 5’ - CCAAGAGGTGAGTGCTTCCC - 3’<br>R: 5’ - CTGTTGTTCAGACTCTCTCCCT -3’
Mouse-TNF-α	F: 5’ - GGCCCAGACCCTCACACTCAGATCAT - 3’<br>R: 5’ - GCAAACCACCAAGTGGAGGA -3’
H5N1-HA	F: 5’- CGCAGTATTCAGAAGAAGCAAGAC-3’<br>R: 5’- TCCATAAGGATAGACCAGCTACCA-3’
